# TMB as a predictive biomarker for ICI response in TNBC: current evidence and future directions for augmented anti-tumor responses

**DOI:** 10.1007/s10238-025-01892-9

**Published:** 2025-11-18

**Authors:** Rajdeep Das, Sneha Deb, P. K. Suresh

**Affiliations:** Department of Bio-Medical Sciences, School of Bio Sciences and Technology, Vellore Institute of Technology, Vellore, India

**Keywords:** TNBC, TMB, ICI response, ICI therapy, Precision therapy

## Abstract

The cardinal features of triple-negative breast cancer (TNBC) are that it is aggressive and does not express estrogen receptor, progesterone receptor, and human epidermal growth factor receptor 2, presenting significant therapeutic challenges. Recent advances have identified immune checkpoint inhibitors (ICIs), especially anti-PD-1/PD-L1 agents, as promising treatments for TNBC. Tumor mutational burden (TMB) has been implicated as a promising approach for predicting ICI responses. This review examines the current evidence in favor of TMB as a biomarker, its relationship with ICI efficacy, and future directions in TNBC therapy. We explore the mechanisms linking high TMB to enhanced neoantigen presentation, which leads to improved immune responses and better patient outcomes with ICIs. Additionally, we discuss the role of combination therapies involving ICIs with chemotherapy or poly(ADP-ribose) polymerase inhibitors, and how TMB can guide treatment personalization in TNBC. While TMB shows promise, further research is needed to establish standardized measurement techniques and cutoffs, enhancing its clinical utility in predicting ICI response. Future studies focusing on tumor microenvironment interactions and resistance mechanisms are crucial for advancing precision oncology in TNBC.

**Graphical abstract:**

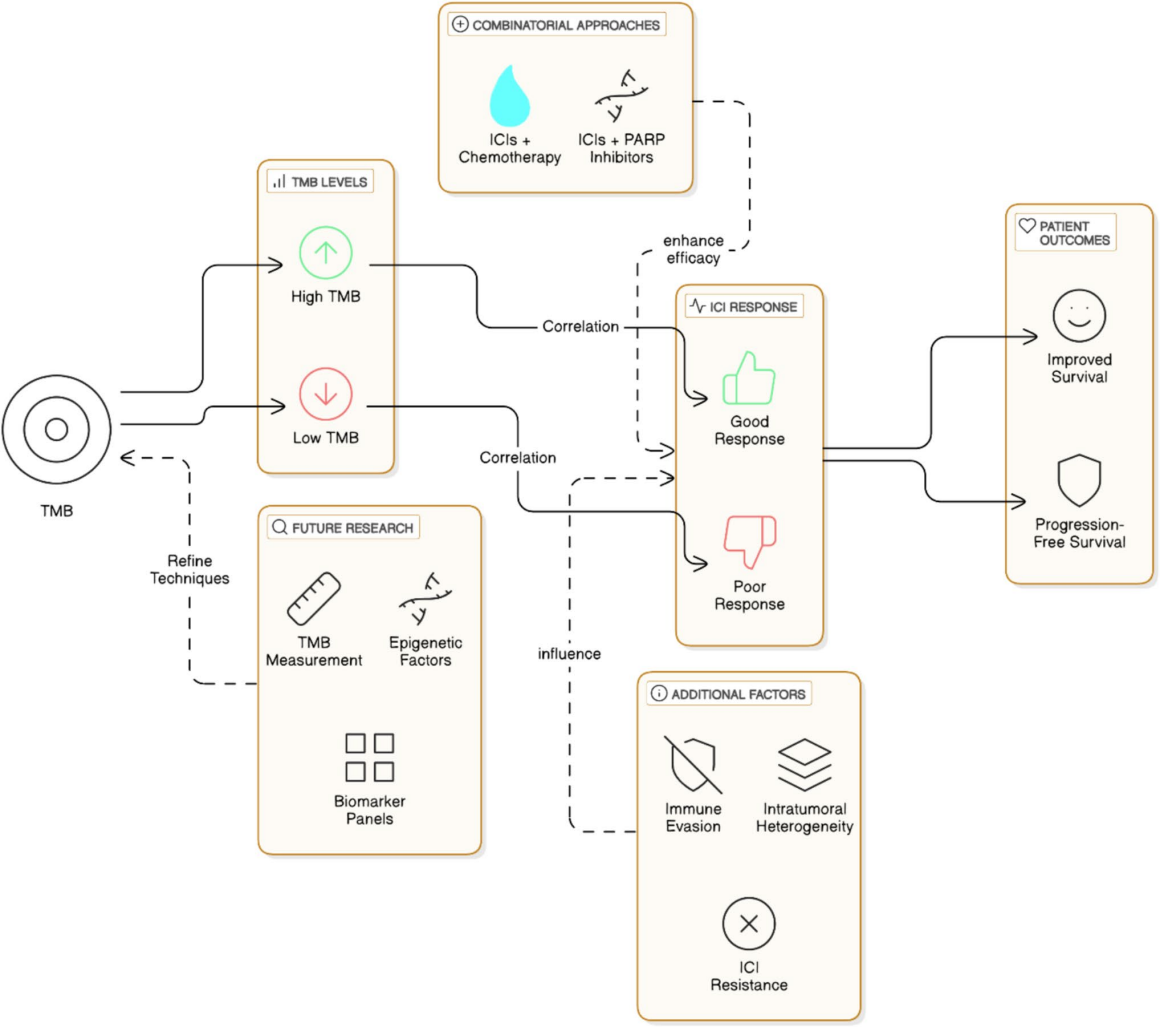

**Supplementary Information:**

The online version contains supplementary material available at 10.1007/s10238-025-01892-9.

## Background

### Background on triple-negative breast cancer

TNBC, a subtype of breast cancer, does not express ER, PR, and HER2 expression. Approximately 15% of all breast cancers belong to this subtype [1]. TNBC is known for its aggressive nature, high rates of recurrence, and poor prognosis compared to other breast cancer subtypes [2]. Studies have demonstrated that TNBC is more prevalent in certain high-risk groups, such as younger women and Black women [1]. In the USA, TNBC accounted for 12% of breast cancers diagnosed between 2012 and 2016, with a 5-year survival rate 8% to 16%, which is lower than hormone receptor-positive disease [1]. A systematic review estimated the pooled prevalence of TNBC in India at 25.04%, higher than in Western countries [3].

The treatment landscape for TNBC poses tremendous challenges due to the lack of targeted therapies like those available for hormone receptor-positive or HER2-positive breast cancers [4]. TNBC is associated with preferential relapses in visceral organs, e.g., the central nervous system, making it particularly difficult to treat [5]. Neoadjuvant chemotherapy has shown higher response rates in TNBC in comparison with other subtypes of breast cancer, a phenomenon known as the TNBC paradox [6]. However, despite this initial responsiveness to chemotherapy, TNBC remains aggressive and is linked to an overall poorer response to therapy, leading to inferior clinical outcomes [4].

Research has highlighted various factors associated with TNBC, including genetic predisposition. Women with TNBC have been found to have a relatively higher prevalence of pathogenic variants, with 23% of women with triple-negative tumors showing these variants [7].

Additionally, TNBC is more common in individuals with a family history of breast cancer, particularly those carrying deleterious germline mutations in genes like BRCA1. In this regard, it has been shown that approximately 57–80% of BRCA1-associated breast cancers are triple-negative in terms of their phenotype [8].

Furthermore, metabolic factors like obesity and metabolic syndrome have been linked to TNBC. Metabolically activated macrophages in obese mammary adipose tissue have been identified as a source of interleukin-6 (IL-6), which promotes TNBC stemness and tumorigenesis through GP130 signaling [9]. The association between metabolic syndrome and TNBC has been noted, with TNBC exhibiting a significantly higher histological grade and a relative absence of ductal carcinoma in situ (DCIS) [10].

TNBC is also characterized by its molecular features, with studies identifying differential gene expressions associated with this subtype. For instance, among other genes, the differential expression of genes like histone clusters spindle and kinetochore associated complex subunit 3 has been linked to TNBC, providing insights into the transcriptional features of this aggressive breast cancer subtype. Additionally, the expression of certain genes like TOMM22 and T-cell receptor genes has been linked with survival in TNBC, suggesting their potential as biomarkers for better predicting the etiology and progression of the disease.

### Immune checkpoint inhibitors in TNBC

ICIs have emerged as a promising treatment for TNBC, a particularly aggressive subtype with limited treatment options [11, 12]. ICIs, such as anti-PD-1/PD-L1 agents, work by enhancing anticancer immune responses and have shown efficacy in both metastatic and early-stage TNBC [13]. Clinical trials, including KEYNOTE-522 and KEYNOTE-355, have led to the FDA approval of pembrolizumab, in combination with chemotherapy, for early-stage and metastatic TNBC [14]. While ICI monotherapy has shown limited responses, combination therapies with chemicals and targeted agents, and radiation have demonstrated improved outcomes [12, 13]. However, challenges remain, including optimizing patient selection, reducing resistance, and managing immune-related adverse events [15]. Ongoing research focuses on identifying biomarkers of response and exploring novel combination strategies to enhance the efficacy of ICIs in TNBC treatment [14].

**Fig. 1:**
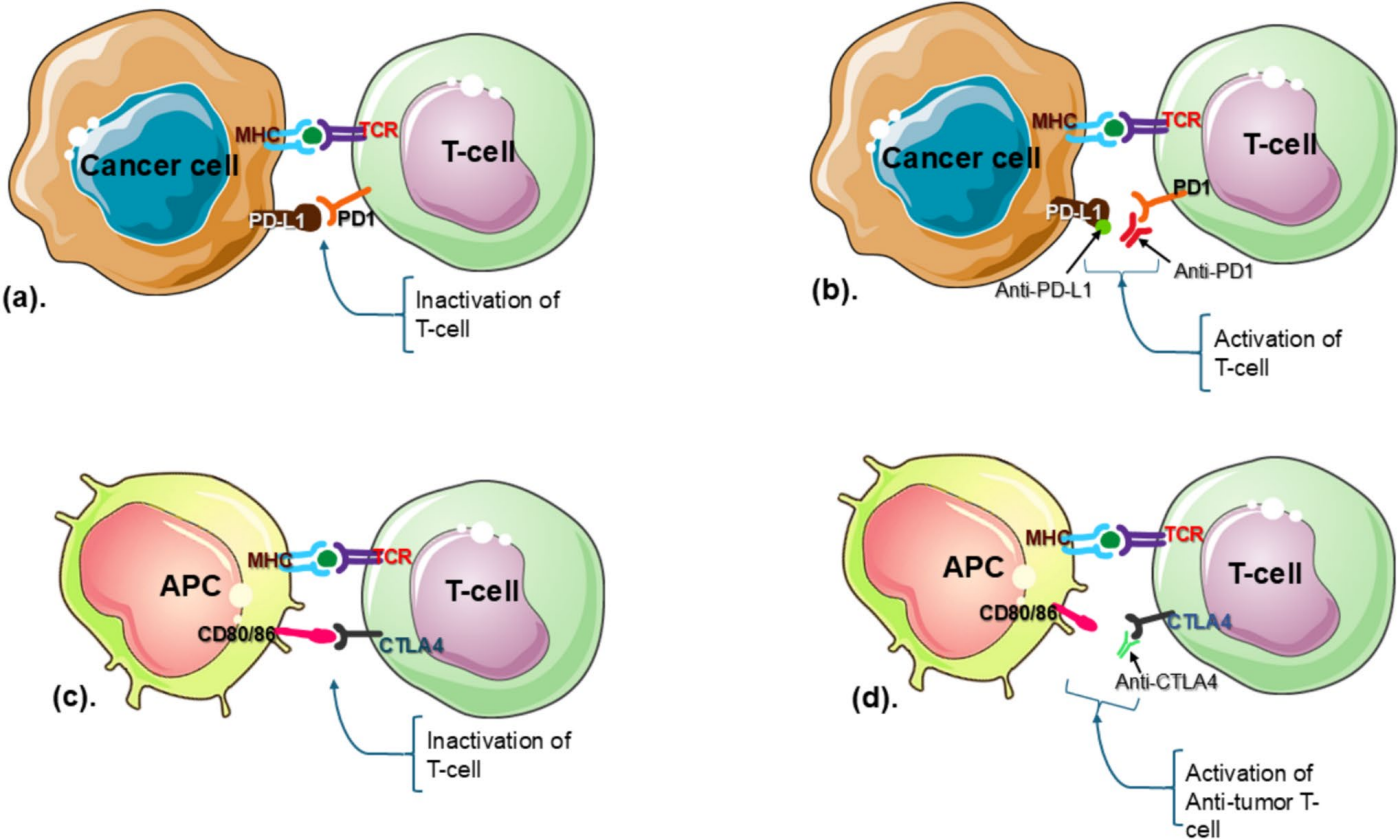
ICI mechanism in cancer immunotherapy; **a** cancer cells express PD-L1, which binds to PD-1 on T cells, leading to T cell inactivation; **b** immune checkpoint blockade using anti-PD-1 or anti-PD-L1 antibodies prevent PD-1/PD-L1 interaction, restoring T cell activation; **c** antigen-presenting cells (APCs) express CD80/86, which interact with CTLA-4 on T cells, leading to T cell inactivation; **d** anti-CTLA-4 antibodies block the inhibitory CTLA-4 interaction, promoting anti-tumor T cell activation

Immune checkpoints are the negative regulatory components of the immune system that play a vital role in self-tolerance [16, 17]. In case of clearance of “faulty” self-cells, these components must be turned off in order to activate our immune system. ICIs are small oligopeptides that act upon the components of immune checkpoint and transmit an array of feedback and feed-forward signals to turn off the immune regulatory checkpoint and help in activating the immune response. From this point of view, the researchers suggested that this part of the immune system might be useful in the therapeutic uses against various kinds of cancers. Allison and Honjo (1990s) mainly worked on this field of improving the immune system’s ability to recognize and eliminate cancer cells by blocking inhibitory molecules on immune cells or tumor cells. They discovered that some proteins present on the surface of the T-cell, such as CTLA-4 and PD-1 mainly act as “brakes” in the case of T-cell activation (the negative regulation of the immune system). Applying these negative regulators can contribute to an inhibition of the clearance of the pathogens (as illustrated in Fig. 1a, c). In an effort to facilitate to block the function of these CTLA-4 and PD-1, they exploited monoclonal antibodies raised against CTLA-4 and PD-1, i.e., “anti-CTLA-4 antibody” and “anti-PD-1 antibody” [18] (as illustrated in Fig. 1b, d).

ICIs, particularly programmed cell death protein 1 (PD-1) and programmed death ligand 1 (PD-L1) inhibitors, have shown promise as therapeutic agents in TNBC [19]. These inhibitors work by inhibiting immune checkpoint proteins, reactivating T immune responses against tumors, and achieving anti-tumor effects [20]. The development of ICIs has initiated the era of immunotherapy for TNBC, offering substantial improvement in terms of the overall prognosis for TNBC patients [21]. Clinical trials have shown that dual application of immune checkpoints inhibitors, such as the blockade of CTLA-4 and PD-1/PD-L1 pathways, is a promising approach in improving the treatment response in TNBC patients [22].

While ICIs are considered a promising strategy for TNBC patients due to their effectiveness for highly immunogenic cancers, results from initial clinical trials have shown benefits in only a subset of patients [23]. However, there has been an improvement in the outcome in a subset of patients treated with ICIs, in both early-stage and metastatic settings [24]. Myc-mediated inhibition of immune cells may compromise the efficacy of ICI therapy in TNBC and BRCA-mutated breast cancer, thereby highlighting the role of the complex tumor microenvironment in influencing treatment outcomes.

Despite these inherent challenges and Tumor microenvironment (TME)-related complexities, the use of ICIs in TNBC treatment has shown considerable potential for improving patient responses, due to the TNBCs being more immunogenic, compared to other types of breast cancer [25]. Additionally, the combination of CDK4/6 inhibitors with other targeted therapies like sitravatinib has been shown to modify the immune landscape of TNBC to favor immune checkpoint inhibitor blockade, indicating the potential for synergistic effects with combination therapies [26]. Recent studies have identified immunotherapy targets in TNBC, such as *Lgals2*, suggesting new avenues for enhancing the efficacy of immune-based treatments in this subtype of breast cancer [27].

The role of immune checkpoint inhibitors in TNBC has expanded into neoadjuvant settings. In Phase III trials, drugs like pembrolizumab and atezolizumab have shown progression-free survival benefits [28]. The identification of HLA class I-restricted immunogenic neoantigens in TNBC has provided insights into potentially new targets for immunotherapy, further supporting the use of possible vaccination strategies to facilitate faster tumor responses to ICI therapy in this breast cancer subtype [29]. Clinical data point to the potential of PD-1/PD-L1-based ICIs in treating TNBC, despite challenges posed by factors like tumor hypoxia, low PD-L1 expression, and immunosuppressive cells [30].

### Tumor mutation burden

TMB, a crucial biomarker in cancer, is defined as the number of somatic mutations per megabase of genomic sequence and is a biomarker with considerable potential for predicting therapeutic response to ICIs across various cancers [31–33]. TMB can be reliably estimated using next-generation sequencing assays [NGS), making it accessible for clinical use [31, 34]. In TNBC, higher TMB is linked with increased benefit from ICIs, both in combination with chemotherapy and monotherapy [35]. A meta-analysis of 26 studies involving 5,712 patients demonstrated that high-TMB groups showed better overall survival and progression-free survival compared to low-TMB groups when treated with ICIs [36]. However, standardization of TMB calculation methods and cutoff values is crucial for its successful implementation as a clinical biomarker [32, 35].

TMB is significant in cancer prognosis and therapeutic response, particularly in the context of immunotherapy using ICIs [37]. High TMB levels have been correlated with a better response to ICIs across various cancer types [38]. The rationale behind this lies in the fact that tumors with greater TMB tend to have more neoantigens, which can be recognized by the immune system, leading to enhanced anti-tumor immune responses [39].

Among the various types of TMB measurement methodologies, panel sequencing-based estimates of TMB (psTMB) have gained prominence as a predictive biomarker for ICI therapy [40]. Panel sequencing allows for the estimation of TMB by analyzing a subset of genes rather than the entire exome, providing a more practical and cost-effective approach [40]. The accuracy and reliability of such TMB measurements are crucial for its clinical utility, as evidenced by studies that have highlighted the impact of TMB levels on treatment outcomes [41].

With reference to TNBC, like in other cancers, the assessment of TMB has shown promise in predicting responses to ICI drugs. Being an aggressive tumor subtype, TNBC with limited treatment options makes the identification of predictive biomarkers like TMB crucial for personalized therapy [42]. Studies have indicated that, like in other cancers, TNBC patients with high TMB levels may exhibit improved responses to ICIs, suggesting that TMB assessment could aid in treatment decision-making for this challenging subtype of breast cancer [42].

Moreover, the influence of factors such as age and sex on the predictive value of TMB in immunotherapy response has been explored, highlighting the need for a complete analysis of the interplay between TMB and patient characteristics [43, 44]. These considerations are essential for optimizing the use of TMB as a predictive biomarker and ensuring its clinical relevance across diverse patient populations [43, 44].

## Materials and methods

### Literature search and selection criteria

This narrative review was conducted by systematically searching various academic databases including EMBASE, PubMed, Directory of Open Access Journals (DOAJ), Scopus, and ScienceDirect without temporal limitations from the inception of the investigation timeline in September 2023. Two investigators (RD and SD) performed comprehensive database searches to ensure methodological rigor and minimize selection bias. The search strategy employed the following Boolean combination of key terms: ("triple negative breast cancer" OR TNBC) AND (("tumor mutational burden" OR (TMB OR ("mutational load" OR ("somatic mutations" OR neoantigens)))) AND (("immune checkpoint inhibitor" OR (PD-1 OR (PD-L1 OR (CTLA-4 OR (pembrolizumab OR (nivolumab OR (atezolizumab OR immunotherapy))))))) AND ("predictive biomarker" OR ("treatment response" OR ("clinical response" OR (survival OR prognosis))))). During the formal investigation process, manual searches were conducted through examination of reference lists and related citations from retrieved articles to identify additional relevant studies.

The retrieved studies were systematically filtered and selected based on inclusion criteria (mentioned below) to ensure relevance and methodological quality. Studies were included if they involved TNBC patients or subjects, provided explicit definition of tumor mutational burden (TMB), measured or compared TMB with study outcomes, applied immune checkpoint inhibitor (ICI)-mediated therapy either as monotherapy or in combination regimens, reported empirically measured study outcomes, and were published as peer-reviewed articles without errata. Any discrepancies were addressed and resolved through consultation with a third/corresponding author (SPK).

Pooled hazard ratios and survival outcomes from published meta-analyses included in the review were extracted directly from the conclusions and summary statistics reported in the respective cited sources, ensuring that meta-analytic findings presented in the discussion are fully based on authoritative sources in the literature.

#### Stratification of data based on confidence levels

##### Evidence from meta-analyses and systematic reviews (highest confidence level)

Large-scale meta-analyses provide the most robust support for TMB's predictive value. A comprehensive meta-analysis of 32 studies encompassing 6131 participants across multiple cancer types demonstrated that high-TMB groups receiving ICIs showed significantly improved overall survival (HR 0.61, 95% CI 0.53–0.71; *P* < 0.01) and progression-free survival (HR 0.51, 95% CI 0.44–0.60; *P* < 0.01) compared to low-TMB groups [45]. However, breast cancer-specific systematic reviews remain limited, with only 5% of all breast cancers demonstrating TMB ≥ 10 mut/Mb, indicating a smaller potentially responsive population compared to other malignancies [46].

##### Evidence from randomized clinical trials (moderate to high confidence level)

Prospective randomized controlled trials provide mixed but encouraging results. The KEYNOTE-355 trial demonstrated improved progression-free survival in PD-L1-positive (CPS ≥ 10) metastatic TNBC patients receiving pembrolizumab plus chemotherapy, though TMB stratification was not a primary endpoint [47]. The KEYNOTE-522 trial showed increased pathological complete response rates (64.8% vs. 51.2%) with neoadjuvant pembrolizumab, with benefits observed regardless of PD-L1 status, suggesting potential TMB-independent mechanisms [48].

The IMpassion130 trial demonstrated PFS benefit with atezolizumab plus nab-paclitaxel in PD-L1-positive TNBC, while the contradictory IMpassion131 results highlight the complexity of biomarker–therapy interactions and potential confounding factors including chemotherapy backbone selection [47].

##### Evidence from cohort studies and retrospective analyses (moderate confidence level)

Large retrospective cohort studies provide valuable real-world evidence but with inherent limitations. A recent pancancer analysis of 1662 patients showed high-TMB cancers had significantly longer overall survival than low-TMB cancers when treated with FDA-approved ICIs, though TNBC-specific subgroup analysis revealed smaller effect sizes compared to lung cancer and melanoma. In TNBC-specific cohorts, high TMB correlated with improved outcomes in metastatic settings, with hazard ratios ranging from 0.65 to 0.69 for overall survival, though confidence intervals often crossed unity due to small sample sizes.

##### Evidence from preclinical studies (lower confidence level)

Preclinical studies using TNBC cell lines, organoids, and xenograft models consistently demonstrate enhanced ICI efficacy in high-TMB contexts, with increased neoantigen presentation and T-cell infiltration. However, these mechanistic insights must be interpreted cautiously given known limitations in translating preclinical findings to clinical practice, particularly regarding tumor microenvironment complexity and immune cell interactions.

## The relationship between TMB and ICI response in TNBC

### Evidence linking TMB to ICI efficacy

Recent studies have highlighted TMB as a promising biomarker for predicting response to ICIs across various cancer types, including TNBC. Meta-analyses have shown that higher TMB levels correlate with improved overall survival and progression-free survival in patients receiving ICIs [36, 49]. Furthermore, the combination of TMB with other factors, such as immune gene expression profiles, has been suggested further to enhance the prediction of treatment response in TNBC patients [13]. However, the relationship between TMB and ICI-related toxicity remains unclear [49]. Currently available data and the inferred TMB-based, patient-related outcomes are given in Table 1.

**Table 1:** Impact of TMB on patient outcomes in TNBC

Range of TMB	PFS	OS	Patient response rate	Associated biomarkers	Therapeutic implementations	Clinical trial/references
< 10 mut/Mb	Shorter PFS (median: 5.6 months with chemotherapy alone)	Poorer OS, HR: > 0.65 compared to high TMB	Lower likelihood of durable response to ICIs	Lower PD-L1 expression, fewer immune cell infiltrates	Limited benefit from ICIs; may require alternative strategies	KEYNOTE-355 [45]
	Median: 3.7 months (*P* = 0.04)	14.2 months (*P* = 0.06)	ORR (30%) (Result did not reach Statistical significance, i.e., *P* = 0.09)	Higher prevalence of PTEN alterations, associated with lower ORR (6% vs. 48%; *P* = 0.01), shorter PFS (2.3 months vs. 6.1 months; *P* = 0.01), and shorter OS (9.7 months vs. 20.5 months; *P* = 0.02)	PTEN alterations were linked to worse clinical outcomes, indicating that patients with these alterations had poorer responses to anti-PD-1/L1 therapies	[50]
≥ 10 Mut/Mb	Improved PFS (median: 9.7 months with pembrolizumab + chemotherapy)	Better OS, HR: 0.65 indicating substantial survival benefit	Higher likelihood of durable response to ICIs	TP53 mutations, higher PD-L1 expression, CD8 + T cell infiltrates	Potential eligibility for ICIs and combination therapies; integration with chemotherapy enhances efficacy	KEYNOTE-355 trial, meta-analysis [45]
	Median: 12.5 months (*P* = 0.04)	29.2 months (*P* = 0.06)	ORR (58%) (Result did not reach Statistical significance, i.e., *P* = 0.09)	High-TMB tumors had a 30% PD-L1 positivity rate, while non-high-TMB tumors had a 41% rate, showing no significant correlation (*P* = 0.7)	mTNBCs treated with ICIs either monotherapy or in combination (with such as, eribulin, nab-paclitaxel, cabozantinib, niraparib) overall patient outcome was better than the low-TMB group	[50]
	7.3 months (Low bTMB) versus 4.1 months (high bTMB) (*P* = 0.012)	–	Low bTMB (< 6.7 mut/Mb) showed better ORR (50%) than high bTMB (≥ 6.7 mut/Mb) *P* = 0.015	bTMB and MSAF as potential predictive biomarkers, ow bTMB and low MSAF demonstrated significantly better ORR and PFS	Combined therapy: anlotinib + benmelstobart (TQB2450)	[51]

*bTMB* Blood-based TMB, *MSAF* Maximum Somatic Allele Frequency, *PFS* Progression-Free Survival, *ORR* Objective Response Rate, *OS* Overall Survival

In TNBC specifically, high TMB levels have been observed in a subset of patients, and TMB has been evaluated as a potential biomarker to predict response to ICIs [52]. Additionally, studies have highlighted the importance of immune-related factors, such as PD-L1 expression and immune cell infiltration, in conjunction with TMB levels in determining ICI treatment outcomes in TNBC [53, 54].

Moreover, as stated earlier, the predictive value of TMB in guiding immunotherapy response has been demonstrated in various cancer types, including lung cancer, colorectal cancer, and melanoma [55–57]. TMB has been associated with a stronger immune response to ICIs, potentially due to the increased likelihood of tumor cells with higher TMB being recognized by the immune system [58]. Notably, TMB quantification has been associated with ICI response and has been practically feasible for clinical assessment [59].

TNBC exhibits a distinctive mutational profile and high immunogenicity compared to other breast cancer subtypes [60–62]. TNBC is characterized by elevated tumor mutational burden, increased immune cell infiltrates, and higher expression of immune-related genes, including those associated with immune checkpoints and regulatory T cells [60]. The immunogenic nature of TNBC has significant implications for immunotherapy, which has shown promising results in both early and metastatic stages of the disease [63, 64]. TP53 mutations in TNBC are correlated with increased expression of immune checkpoint and cancer testis gene sets [60]. Moreover, specific mutations within the PIK3CA gene have been shown to influence ICI efficacy. PIK3CA mutations can lead to altered signaling pathways that affect tumor microenvironment characteristics, such as immune cell infiltration [65, 66]. While PD-L1 expression is considered predictive of drug efficacy in response to checkpoint inhibitor therapy, challenges remain in its use as a biomarker [63]. The complex interplay between immune infiltration and certain widely reported and accepted mutational signatures in TNBC warrants further investigation to optimize immunotherapeutic strategies [64].

Recent studies utilizing high-throughput genomic data analysis have suggested that different TMB value subsets of a TNBC patient cohort may exhibit differences in immune cell infiltration [67]. However, more comprehensive studies across large TNBC patient cohorts are necessary to definitively ascertain whether TMB levels significantly differ between subtypes, thereby impacting prognosis or treatment responses. Such investigations would ultimately aid physicians in developing more effective treatment regimes. Lehmann et al. have reported that TNBC can be further categorized into seven distinct subsets based on their gene expression profiling. These subsets comprise two basal-like subtypes, namely BL1 and BL2, an immunomodulatory subtype (IM), a mesenchymal subtype (M), a mesenchymal stem-like subtype (MSL), a luminal androgen receptor subtype (LAR), and an unstable subtype (UNS) [68, 69]. The classification system for TNBC has undergone revisions, resulting in a reduction from to four subtypes (TNBC type-4: BL1, BL2, M and LAR). These changes were implemented in order to increase the precision and fidelity of the subtyping system, thereby ensuring its alignment with the authentic molecular attributes of TNBC tumors [70]. Consequently, this update furnishes more accurate information for the purposes of diagnosis, prognosis, and treatment selection. In order to elaborate upon what was stated earlier, TMB can be quantified through a variety of methods (discussed in detail in 4.2), including NGS of DNA extracted from formalin-fixed, paraffin-embedded (FFPE) tissue samples or the use of targeted panels such as the MSK-IMPACT panel or the Oncomine™ Tumor Mutational Load (OTML) assay. However, it is important to select an appropriate cutoff value for the tumor type being evaluated in order to obtain a reliable and clinically meaningful TMB score that can be used to accurately define high TMB in order to demarcate those that do not exhibit a relatively high value.

Neoantigens specific to tumors have been linked with the presence of a high number of non-synonymous mutations, activating robust anti-tumor immune responses that can be leveraged by immunotherapeutic agents like ICIs [71]. TNBC exhibits a higher mutational burden compared to other breast cancer subtypes, rendering it the most immunogenic subtype [72]. The high expression levels of tumor-infiltrating lymphocytes (TILs), programmed death ligand protein 1 (PD-L1), coupled with an increased TMB; microsatellite instability (MSI), and mismatch repair deficiency (MMR) in TNBC make it a potential candidate for immunotherapy [73, 74].

The heterogeneity of TNBC poses challenges in the appropriate selection of patients for immunotherapies, emphasizing the need for personalized treatment approaches [75]. The immune microenvironment of TNBC plays a crucial role in determining patient response to immunotherapy, with immune-favorable subtypes, wherein the subjects with a low TME-related gene score exhibited a better response to immune treatments [76]. TNBC's high immunogenicity and the potential for eliciting a strong immune response make it a suitable candidate for immunotherapy [20].

### Tumor immune phenotype and their impact on TMB-ICI response relation

The tumor immune microenvironment (TiME) significantly influences ICI efficacy beyond TMB alone, with three distinct immunological phenotypes determining therapeutic outcomes in TNBC.

Immune-inflamed ("hot") tumors exhibit enhanced IFN-γ responsive gene expression, high PD-L1 expression, and substantial tumor-infiltrating lymphocytes (TILs) within the tumor core [77]. These tumors typically demonstrate high TMB and microsatellite instability, providing unique neoantigen profiles that facilitate robust anti-tumor immunity.

Immune-excluded ("cold") tumors are characterized by immune cells confined to tumor stroma without infiltrating the tumor parenchyma, rendering them unresponsive to ICIs despite adequate immune cell presence. This phenotype utilizes immunosuppressive mechanisms including increased regulatory T cells, M2-polarized macrophages, elevated myeloid-derived suppressor cells, and enhanced TGF-β signaling [78]. Notably, TGF-β inhibition can convert this phenotype to an inflamed state, sensitizing tumors to PD-L1 inhibitors.

Immune desert tumors show minimal TIL presence, reduced MHC class I expression, and typically low TMB, and demonstrate poor ICI monotherapy response [79]. Reduced chemokine gradients through WNT/β-catenin signaling may impair dendritic cell recruitment which is essential for T cell priming [80].

Recent work using spatial immunophenotyping in TNBC demonstrates that “inflamed,” “excluded,” and “desert” patterns can shift over time or after PD-1 blockade, reflecting dynamic stromal remodeling and T cell invasion paths [81]. Longitudinal profiling through serial biopsies has revealed treatment-induced transitions from immune‐cold to immune‐hot microenvironments, with neoantigen load and emerging resistance clones driving these shifts [82]. Spatial transcriptomics further refines this view by deconvoluting epithelial from stromal compartments—either via primer‐based capture panels or laser‐capture microdissection—to minimize stromal contamination and enable high‐resolution mapping of TMB–immune correlations [83, 84]. Complementary single‐cell RNA sequencing analyses have linked elevated TMB to the expansion of exhausted CD8^+^ T cells and M2 macrophages following ICI therapy, highlighting the importance of cell‐type-specific dynamics in immunogenomic cross talk. While each of these approaches offers unparalleled insight into the temporal and spatial heterogeneity of the tumor immune landscape, they also present practical challenges: serial sampling can be invasive and limited by tissue access, spatial assays incur high cost and computational complexity, and scRNA‐seq requires careful interpretation to account for sampling bias. Together, these methods emphasize that any static biomarker interpretation—TMB included—must be contextualized within the evolving immune milieu of TNBC.

The relationship between TMB and these phenotypes is complex—while high TMB generally correlates with better ICI responses, the immune contexture ultimately determines outcomes. High-TMB tumors with favorable immune profiles, such as immune-inflamed phenotype, show improved responses [31, 77], whereas immunosuppressive microenvironments can negate TMB benefits. Furthermore, the incorporation of multi-omic analyses—including single-cell sequencing, proteomics, and spatial transcriptomics—could significantly deepen our understanding of the interplay between mutational burden and immune phenotypes (Table 2).

**Table 2:** Comparative performance of major TMB measurement platforms

Platform/methods	Coverage	Sensitivity	Specificity	Reproducibility	Major limitations	
WES	~ 30–50 Mb (all protein-coding exons)	H for moderate/high TMB; M in low tumor purity	H with robust pipelines and matched normals	M–H (laboratory/pipeline-dependent)	Costly, longer TAT, requires high input DNA and often matched normal, batch effects, inter-pipeline variability	[85–89]
Targeted Tissue NGS Panels (e.g., 0.67–1.5 Mb, FoundationOne CDx/MSK-IMPACT class)	~ 0.5–1.5 Mb (curated cancer genes)	~ 0.5–1.5 Mb (curated cancer genes)	M–H (depends on filtering and panel composition)	M–H within assay; lower between assay	Panel design bias, smaller territory can misclassify low-TMB tumors, cross-vendor cutoff discordance, reliance on calibration	[85–88, 90]
bTMB	~ 0.5–1.0 + Mb (panel-dependent) from plasma cfDNA	M (limited by tumor fraction, good in high-shed tumors)	M with stringent filtering	L-M (affected by pre-analytics and tumor fraction)	Low tumor fraction, clonal hematopoiesis confounding, pre-analytical variability, may not reflect spatial heterogeneity fully	[85, 86]
psTMB	Varies (depends on base panel)	M–H (improves alignment to WES vs. raw panel counts)	M–H (depends on germline/pathogenic filtering)	M–H within a given laboratory	Requires accurate modeling of panel dimensions and background, still panel-dependent, needs validation	[85, 87, 88]
ecTMB	Computational (applies to WES or panel inputs)	H vs. simple counting (better correlation to WES)	H (model-based background mutation estimation)	H once parameters are fixed	Requires model training/validation, assumptions may vary across tumor types, still dependent on input data quality	[91]
Targeted Panels with Enhanced Filters (e.g., COSMIC-guided, inclusion of synonymous mutations)	Same as “Targeted Tissue NGS Panels”	H (reduces overestimation vs. naïve counts)	H (improved false positive control)	M–H (if filters standardized)	Requires curated databases, pipeline updates can shift estimates, residual panel bias	[90]

Coverage = genomic territory

Sensitivity/Specificity/Reproducibility reflects typical performance across studies/platforms

*H* High, *M* Moderate, *L* Low

### Toward multimodal biomarker strategies: beyond TMB

TMB alone does not suffice as a biomarker for guiding therapy in TNBC, mainly due to the intricate interactions among mutational load, immune cell infiltration, and tumor microenvironment heterogeneity. TILs robustly predict treatment response and prognosis, indicating the extent of endogenous anti-tumor immunity [92, 93]. Immune gene expression signatures, particularly those related to IFN-γ and cytolytic activity, reflect the functional status of the tumor microenvironment, thereby enhancing patient stratification alongside TMB [46, 67]. Although MSI is rare in TNBC, its presence marks a subset of tumors with heightened immune response potential [94, 95]. Furthermore, the role of epigenetic regulators, such as microRNAs, is increasingly acknowledged in modulating immune escape mechanisms and ICI efficacy [96]. The integration of these biomarkers into composite predictive models is encouraged to refine immunotherapy selection for TNBC, aiming to tackle the inherent clinical heterogeneity and improve patient outcomes [46, 97].

### Mechanism of action

Higher TMB is associated with increased neoantigen production, enhancing T cell recognition and activation [98–101]. Research demonstrates a strong link between TMB and the clinical efficacy of PD-1/L1 monotherapy and combination ICIs with PD-1/L1 plus CTLA-4, with high-TMB tumors showing the greatest benefit [49]. However, TMB cannot predict ICI responses accurately, due to intra-tumor heterogeneity, which can hinder efficient neoantigen recognition by T cells [102]. Patients with high TMB exhibited improved overall survival and progression-free survival when treated with ICIs compared to chemotherapy alone, while low-TMB patients did not show significant benefit [36]. Future studies should aim to develop composite predictors incorporating additional variables such as MHC and T cell receptor repertoire, to better predict survival [98].

TMB plays a significant role in modulating immune responses and enhancing the effectiveness of ICIs. Studies have demonstrated that tumors with elevated TMB exhibit a higher response rate to ICIs, as they tend to harbor a greater abundance of tumor-infiltrating lymphocytes, including CD8^+^ T cells, which are crucial for effective immunotherapy [43, 103, 104]. The correlation between TMB and immune cell infiltration underscores its potential as a predictive marker for ICI efficacy, suggesting that higher TMB may enhance the immunogenicity of tumors and improve patient outcomes in immunotherapy [98, 105].

Neoantigens, which arise from tumor-specific mutations such as single-nucleotide variants (SNVs), insertion or deletions (INDELs), or gene fusions, play a crucial role in T cell activation and the immune response against cancer. Paradoxically, while somatic mutations drive oncogenesis, they simultaneously generate immunogenic neoantigens that render tumors susceptible to immune surveillance. These mutations generate aberrant proteins that are processed intracellularly into short peptides via proteasomal degradation. The resulting neoantigenic peptides are transported to the endoplasmic reticulum, where they bind to MHC class I molecules for presentation on the cell surface, enabling recognition by CD8^+^ cytotoxic T lymphocytes. Alternatively, extracellular antigens captured by APCs are processed into longer peptides associated with MHC class II molecules, activating CD4^+^ T helper cells. The immunogenicity of neoantigens depends on their structural divergence from self-peptides and strong MHC binding affinity, factors leveraged in bioinformatics pipelines to prioritize targets for immunotherapy. Neoantigens, presented by APCs in complex with MHC molecules, can be recognized by T cells and serve as promising targets for immunotherapy [106].

Although tumors with high TMB are theoretically expected to generate a greater repertoire of neoantigens, this mechanistic link is not unequivocally established in TNBC. Many somatic mutations fail to yield peptides that bind major histocompatibility complex molecules, and among those that are presented, only a subset elicits detectable T cell responses [107, 108]. Furthermore, TNBC cells frequently downregulate antigen‐processing and presentation machinery, enabling immune escape despite high mutational loads [109].

Mass spectrometry (MS)-based MHC immunopeptidome profiling offers a direct approach to validate neoantigen presentation by isolating peptide–MHC complexes from tumor tissues and sequencing the bound peptides. This methodology has demonstrated its translational value by identifying clinically actionable neoepitopes in multiple cancer types and correlating neoantigen burden with patient survival [108]. Its strengths include the ability to capture the naturally processed peptidome without reliance on in silico prediction and to discover non-canonical and post‐translationally modified epitopes. However, its limitations—such as the requirement for large quantities of fresh or flash‐frozen tissue, high instrument sensitivity, and complex bioinformatic pipelines—pose significant challenges for routine clinical deployment. Detection sensitivity may miss low‐abundance peptides, and sample heterogeneity can confound interpretation.

To substantiate TMB as a predictive biomarker in TNBC, future translational studies must integrate MHC immunopeptidomics with T cell reactivity assays in patient cohorts stratified by TMB status. Direct epitope identification combined with functional validation will be essential to move beyond correlative analyses and to establish the true immunogenic potential of mutational landscapes in TNBC [110].

Dendritic cells (DCs) are particularly adept at capturing tumor-derived antigens through mechanisms such as phagocytosis, receptor-mediated endocytosis, and autophagy. Once internalized, these antigens are processed into peptides that can be presented on MHC class I and II molecules. This dual pathway is crucial for activating both CD8^+^ cytotoxic T cells and CD4^+^ T helper cells, respectively. DCs uniquely facilitate cross-presentation of extracellular antigens, enhancing migration to lymph nodes and T cell priming [111, 112]. B cells participate by presenting neoantigens in tertiary lymphoid structures, promoting local immune activation and producing antibodies that opsonize tumor cells. Myeloid-derived suppressor cells (MDSCs) also contribute by engulfing tumor debris and presenting neoantigens via MHC class II to CD4^+^ T cells. The tumor microenvironment influences their function; while some MDSCs adopt an immunosuppressive phenotype, activated MDSCs can enhance antigen presentation [111].

**Fig. 2:**
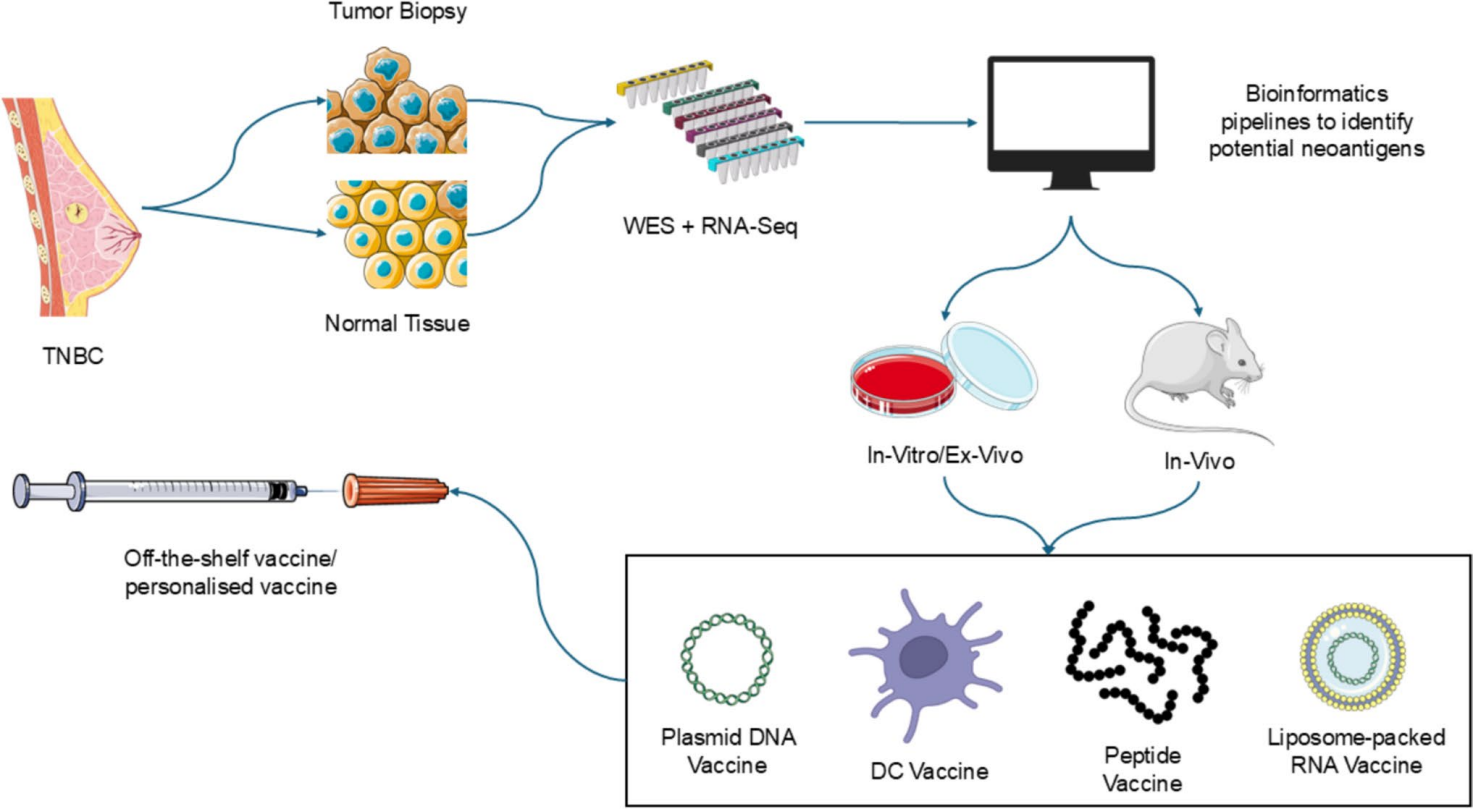
Neoantigen vaccine preparation; tumor biopsy and normal tissue collection, WES and RNA sequencing, using bioinformatics pipelines to identify and predict potential neoantigens, in vivo/ex vivo and in vitro validation of neoantigens, preparation of plasmid vaccine, DC vaccine, peptide vaccine, liposome-packed RNA vaccines

High-TMB tumors often harbor clonal neoantigens shared across patients (e.g., frameshift peptides in MMR-deficient cancers) [113], enabling off-the-shelf vaccines. Subclonal neoantigens require personalized approaches using sequencing and machine learning algorithms (e.g., HLA transgenic mouse models) [114, 115]. Refer Fig. 2 for an overview on neoantigen vaccine preparation.

The generation of neoantigens can be influenced by various factors, including the downregulation of specific proteins, which can enhance T cell responses [116]. Studies have shown that the purified and characterized neoantigens loaded onto dendritic cell vaccines can evoke robust CD4^+^ and CD8^+^ T cell responses, indicating their potential in cancer treatment [117]. Furthermore, the presence of a higher neoantigen load correlates with increased tumor-infiltrating lymphocytes and improved responses to immune checkpoint blockade therapies [118]. Overall, neoantigens are pivotal in shaping the adaptive immune response, providing a sound mechanistic basis for developing personalized cancer vaccines and immunotherapies [119].

### Use in precision therapy

In order to unveil a novel, high-precision therapeutic strategy, it is a mandatory prerequisite to meticulously scrutinize the presently employed targeted therapeutic modalities for TNBC, with the ultimate objective of ascertaining their potential limitations and shortcomings. Supplementary Table 1 (Therapeutic avenues for TNBC [Refer Additional File 1]) presents an assemblage of the therapeutic approaches utilized in the management of triple-negative breast cancer. In Precision medicine, suitable biomarkers are employed to improve the predictive value of targeted therapy in specific categories of patients. In breast cancer therapy, several identified druggable mutations may be utilized for designing a therapeutic regimen. Precision treatment strategies for breast cancer entail the refinement of our comprehension of breast subtypes and treatment biomarkers through the amalgamation of knowledge derived from various data types and evidence-based clinical trials [120]. In the context of TNBC, the identification of distinct subclasses grounded on intrinsic signals and the tumor microenvironment can facilitate prognostication of response to established therapies and pinpoint nascent therapeutic targets [121]. Furthermore, atypical genetic alterations have been investigated as possible indicators for the detection, prognosis, monitoring of treatment effectiveness, and prediction of breast cancer risk. This has led to the possibility of implementing epigenetic modulatory dietary bioactives for precision medicine [122]. The utilization of Dempster–Shafer decision theory (DST) in conjunction with gene expression data has shown promise in augmenting diagnostic precision for breast cancer [123]. Through the integration of pivotal data acquired from gene expression and conventional diagnostics, the DST process epitomizes an elevated standard of diagnostic precision, ultimately culminating in the potential to amplify the efficacy of precision therapy for breast cancer patients.

While the focus of this review is to provide an updated review of TMB and the correlated response to ICIs, other approaches can augment this therapeutic modality and hence need to be highlighted and discussed to further fine-tune and improve the currently available precision strategies for TNBC. In this regard, the current strategies for TNBC involve targeting the DNA damage response (DDR) pathways and cancer stem cells (CSCs) [124]. Usage of PARP inhibitors and immune checkpoint inhibitors has been authorized for TNBC treatment [125]. Inhibitors of the ATR-CHK1-WEE1 pathway and PARP inhibitors have shown potential in preclinical and clinical studies [126]. Next-generation panel sequencing, particularly of somatic exomes, offers hopes for individualized treatment plans [127]. Advanced therapy using genes and nanocarriers, such as liposomes, nanoparticles, and dendrimers, shows promise for the efficient delivery of siRNA and chemotherapeutics [128]. Further evidence points toward checkpoint inhibitors and PARP inhibitors as potential therapies for metastatic TNBC [129]. Different subclasses of TNBC can be identified based on intrinsic signals and microenvironment, which can help in predicting responses to therapies and identifying novel targets [121]. The existing knowledge base with respect to precision medicine and next-generation sequencing in terms of certain molecular markers is also being implemented in clinical practice and trial design [121]. Furthermore, multi-omic approaches have revealed actionable targets such as the PI3K-Akt-mTOR and epidermal growth factor receptor signaling pathways, which may modulate chemosensitivity and the immune system. Combinatorial trials with chemotherapy or targeted agents, as well as immunotherapy, are being researched into to improve outcomes for TNBC patients.

The identification of biomarkers such as epidermal growth factor receptor (EGFR), c-MET, and BRCA1/2 mutations has significantly advanced our understanding with regards to the development of targeted therapies for TNBC [130]. These therapeutic agents are designed to exploit specific vulnerabilities present in TNBC cells, potentially leading to more effective treatments with minimized off-target effects. Notably, novel antibody–drug conjugates (ADCs), such as sacituzumab govitecan, which targets Trop-2, have demonstrated considerable efficacy in patients with heavily pre-treated TNBC [131].

Despite these advancements, several intrinsic limitations persist. The efficacy of these therapies as monotherapies is often limited, necessitating combination approaches for enhanced effectiveness. Additionally, challenges related to the validation of biomarkers remain, as does the emergence of resistance mechanisms, including the overexpression of drug efflux pumps, apart from immune suppression and evasion strategies within the TME to hinder therapeutic outcomes. Adverse events associated with these treatments, such as immune-related adverse events (irAEs), also pose significant clinical challenges that must be addressed in ongoing research and clinical practice.

These strategies aim to optimize treatment efficacy and overcome the heterogeneity and treatment resistance observed in TNBC. Despite chemotherapy being the treatment of choice for TNBC, for the last several decades, it has limited efficacy and can cause severe side effects [132]. Tumor-related variability and subclonal evolution in primary and metastatic TNBC pose challenges for designing adaptive precision medicine-based treatment plans [127]. TNBC has a high relapse rate and metastasis, which makes it difficult to treat [133, 134]. Currently, there is an absence of targeted therapies for TNBC, which makes it challenging to develop effective treatment strategies.

### Resistance mechanism to ICIs in TNBC

One of the intrinsic mechanisms of resistance involves alterations in key signaling pathways that regulate immune responses. For instance, mutations in the phosphatase and tensin homolog (PTEN) gene, which are prevalent in TNBC, can lead to the activation of the phosphoinositide 3-kinase (PI3K) pathway, promoting tumor growth and survival, while simultaneously suppressing anti-tumor immunity [135]. This aberrant signaling can diminish the efficacy of ICIs by creating an immunosuppressive microenvironment that hampers T-cell activation and function [136]. Additionally, the loss of PTEN has been associated with a PTEN-null phenotype (common downstream effect irrespective of the upstream genetic changes) that is resistant to PI3K-alpha inhibition, in tumoral cells [135].

Resistance mechanisms in TNBC have now been dissected in tumor-specific models rather than inferred solely from other malignancies. For example, conditional PTEN knockout together with Myc overexpression in patient-derived organoids recapitulated both epithelial heterogeneity and stromal immune exclusion observed in aggressive TNBC, whereas clinical specimens confirmed frequent PTEN loss and correlated genomic alterations with reduced CD8^+^ infiltration [137, 138]. PI3K/AKT/mTOR pathway hyperactivation has similarly been validated in TNBC cell lines and xenografts, where selective PI3K inhibitors restored antigen presentation and enhanced ICI efficacy, underscoring a tumor intrinsic driver amenable to targeted therapy [139].

Tumor-associated macrophages (TAMs) have been directly implicated in TNBC progression: High CD68^+^ macrophage density in primary tumors correlates with mesenchymal markers, EMT gene signatures, and inferior survival in TNBC cohorts [140], and coculture of TNBC spheroids with M2-polarized macrophages promotes invasive growth and angiogenesis both in vitro and in orthotopic mouse models [141]. Hypoxia-induced metabolic reprogramming further compounds immune evasion in TNBC. Hypoxia markers (e.g., CA9) inversely correlate with TIL density in patient samples, and HIF-dependent glycolytic shifts create an acidic microenvironment that suppresses effector T cells in ex vivo TNBC explants [142].

Conversely, theoretical mechanisms—such as amino acid depletion via IDO1 or arginase in myeloid cells—remain to be rigorously tested in TNBC. Although broad profiling studies suggest that tryptophan catabolism may drive immunosuppression across cancers, functional validation in TNBC organotypic cultures and scRNA-seq of matched primary and metastatic lesions is still limited [143]. Finally, SNAI1-driven CD73 upregulation has been shown to increase adenosine-mediated suppression in TNBC cell lines, but orthogonal confirmation in patient-derived organoids and correlation with response to CD73 blockade in early-phase trials is ongoing [144].

Extrinsic factors contributing to ICI resistance include the tumor microenvironment, which is often characterized by a high presence of immunosuppressive cells such as TAMs and MDSCs. These cells can inhibit T-cell activity and promote tumor progression by secreting immunosuppressive cytokines [145, 146].

TAMs comprise pro-inflammatory M1 and immunosuppressive M2 subsets. A higher M1/M2 ratio correlates with improved ICI responses, as M1 macrophages may enhance T-cell activation, while M2 polarization can contribute to the resistant phenotype [147, 148]. MDSCs, including monocytic (M-MDSC) and granulocytic (G-MDSC) subsets, suppress T-cell activity and accumulate in tumors with high TMB, dampening neoantigen-driven anti-tumor immunity [149, 150].

In TNBC, TAMs predominantly exhibit an M2-like phenotype, which facilitates tumor growth and metastasis while contributing to immune evasion. For instance, high densities of CD163^+^ TIM-3^+^ TAMs have been associated with poor prognosis, as these cells can endocytose anti-PD-L1 antibodies, neutralizing the therapeutic effects of ICIs [151]. Additionally, the stoichiometric relationship between TAMs and CD8^+^ T cells is critical; a TAM-to-CD8^+^ T cell ratio greater than 2:1 has been shown to predict poor responses to ICIs [152, 153]. Trabectedin, a TAM-depleting agent, reduces this ratio and synergizes with anti-PD-1 therapy in preclinical models [153].

TMB alone is insufficient to predict ICI efficacy; the immune contexture is critical. High TMB with favorable immune profiles (elevated M1/M2 ratio, low MDSC infiltration) improves outcomes, whereas immunosuppressive TMEs negate TMB benefits [154]. Metabolic reprogramming further modulates immunity: Tumor glycolytic flux (Warburg effect) generates lactate, suppressing T-cell function, while TAM/MDSC metabolic adaptations enhance immunosuppression [155, 156].

The presence of TAMs has been shown to correlate with poor prognosis in TNBC, as they can facilitate tumor growth and metastasis while also contributing to immune evasion by endocytosing anti-PD-L1 antibodies, thereby neutralizing the therapeutic effects of ICIs [145, 146]. Moreover, high tumor purity has been associated with resistance to ICI therapy, indicating a weaker anticancer immune response, which is further supported by the inverse correlation between tumor purity and immune infiltration [157].

Another critical aspect of resistance mechanisms in TNBC is the role of TMB. However, the relationship between TMB and ICI efficacy is not straightforward in TNBC. While some studies suggest that a greater TMB correlates with better responses to ICIs, other research indicates that the presence of specific mutations may not always translate to improved immunogenicity or therapeutic outcomes [158, 159]. For instance, mutations in genes involved in DNA repair mechanisms, such as BRCA1/2, can lead to increased TMB but may also result in unique immune evasion strategies that complicate treatment responses [160]. A comprehensive meta-analysis by Chen et al. examining 46,870 breast cancer patients across 16 studies demonstrated a strong association between BRCA1 mutations and TNBC, with BRCA1 mutation carriers showing 8.889-fold increased odds of developing TNBC compared to non-carriers (95% CI 6.925–11.410) and 3.292-fold increased odds compared to BRCA2 mutation carriers (95% CI 2.773–3.909). This meta-analysis further confirmed that BRCA1 mutations are associated with more aggressive tumor characteristics, including higher nuclear grade and larger tumor burden [161].

Moreover, the immune landscape of TNBC is further influenced by the immune checkpoint proteins, such as PD-L1. The upregulation of PD-L1 on tumor cells can inhibit T-cell activation and promote immune tolerance, thereby contributing to resistance against ICIs [162–164]. The expression of PD-L1 is often used as a biomarker to predict response to ICIs; however, its presence does not guarantee therapeutic efficacy, as the tumor microenvironment's overall immunogenicity plays a crucial role in determining outcomes [162–164]. PD-L1 is implicated in various mechanisms of cancer cell-related behavior (cell proliferation, colony formation, migration, and invasion). These aberrations at the cellular level were reported to be independent of its interaction with PD-1. Evidence indicates that the knockout of PD-L1 in TNBC cells results in marked reductions in cell proliferation, colony formation, and tumor growth in CAM assay, as well as diminished migration and invasion capabilities [165]. Additionally, CD8^+^ T cells within the TME have been associated with improved responses to ICIs, highlighting the importance of T-cell infiltration in overcoming resistance [166, 167].

The metabolic state of the tumor microenvironment can also affect immune responses, with altered metabolic pathways potentially leading to immune suppression and resistance to therapy, such as lactate and acidosis (Warburg effect as discussed earlier), hypoxia (upregulating PD-L1 in cancer cells and MSDCs, fostering immune evasion and resistance to ICIs), nutrient competition (depleted glucose and amino acids, such as glutamine) [168–170].

In conclusion, the resistance mechanisms to ICIs in TNBC are complex and multifactorial, involving intrinsic tumor characteristics, extrinsic immune factors, and the interplay with TMB. Understanding these mechanisms is necessary for developing more effective therapeutic strategies and improving patient outcomes in this challenging subtype of breast cancer. Future research should place a greater emphasis on elucidating the specific pathways and interactions that contribute to resistance, as well as exploring combination therapies that can enhance the efficacy of ICIs in TNBC patients. By distinguishing mechanisms validated in TNBC models from those requiring further tumor-specific confirmation, this review aims to ground clinical strategies in empirically supported biology rather than oncologic generalizations.

## Other factors that influence the relation between TMB and ICI response in TNBC

### Epigenetic influences on TNBC and ICI response in TNBC

Epigenetic modifications, including DNA methylation and histone modifications, are critical mechanisms that regulate gene expression without altering the underlying DNA sequence. These modifications play a significant role in various biological processes, including development, cellular differentiation, and disease progression, particularly in cancer.

DNA methylation involves adding a methyl group to cytosine residues in CpG dinucleotides, typically leading to transcriptional downregulation of associated genes. Abnormal DNA methylation patterns can inhibit tumor suppressor gene expression and activate oncogenes, thereby contributing to tumorigenesis [171]. In TNBC, specific gene promoter methylation—such as that of the BRCA1 gene—has been linked to a more aggressive disease phenotype and poor prognosis [172].

There are various histone modifications (e.g., acetylation, methylation, and phosphorylation), also play a pivotal role in regulating chromatin structure and gene expression. For instance, histone acetylation is generally associated with transcriptional activation, while methylation demonstrates a more versatile regulatory role, as its effects on gene expression—whether stimulatory or inhibitory—are contingent upon factors such as the specific lysine residues undergoing modification and the broader biochemical milieu in which these changes occur [173]. In TNBC, altered patterns of histone modifications have been observed, which may contribute to the aggressive nature of the disease and its resistance to conventional therapies [174]. The interplay between DNA methylation and histone modifications can create a complex regulatory environment that influences TMB and the efficacy of ICIs in TNBC patients.

Recent studies have begun to explore the relationship between epigenetic modifications and the response to immunotherapy in TNBC. For example, research has indicated that specific histone modifications can affect the expression of immune-related genes, thereby influencing the tumor microenvironment and immune cell infiltration [175]. Histone deacetylase inhibitors (HDACi) have been reported to increase PD-L1 and HLA-DR expression on TNBC cells, improving the ICI response. HDACi also decreases regulatory T cells (*T*_regs_), augment T cell effector function (CD8^+^ T cell cytotoxicity) in the TME, favoring an improved immune response [176, 177]. The inhibition of histone lysine-specific demethylase 1 (LSD1) has been shown to increase H3K4me2 levels at promoter regions of chemokines, enhancing CD8^+^ T cell migration and infiltration in TNBC [178]. This suggests that epigenetic alterations may serve as potential biomarkers for predicting responses to ICIs. Moreover, the presence of TILs, which are critical for effective anti-tumor immunity, has been shown to correlate with specific epigenetic signatures in TNBC [179]. Genes such as IFNG, CTLA-4, FAS, CXCR6, and JUN are overexpressed in CD4^+^ TILs, contributing to lymphocyte exhaustion and affecting chemotaxis, which may influence TNBC progression [180]. A four-gene signature comprising HLF, CXCL13, SULT1E1, and GBP1 has been identified to forecast high levels of TILs after neoadjuvant chemotherapy, correlating with better survival outcomes in TNBC patients [181]. These findings highlight the potential for integrating epigenetic profiling with TMB assessment to enhance predictive accuracy for ICI responses.

In terms of TNBC-specific studies, several investigations have focused on the role of microRNAs (miRNAs) as epigenetic regulators that can modulate gene expression and influence treatment outcomes. For instance, miR-214 has been identified as an oncomiR that promotes metastasis in TNBC by targeting tumor suppressor genes [182]. The expression levels of certain miRNAs have been correlated with TMB and the response to chemotherapy and immunotherapy, suggesting their utility as biomarkers for treatment stratification [183]. Additionally, the epigenetic landscape of TNBC has shown to be influenced by environmental factors, such as diet and inflammation, which can further modulate the correlation between TMB and ICI response [184].

The potential for epigenetic biomarkers to complement TMB in predicting ICI response is an area of active research. Epigenetic modifications are reversible and can be influenced by various factors, including lifestyle and therapeutic interventions, underscoring their potential as dynamic biomarkers [185]. For example, the identification of specific DNA methylation patterns or histone modification profiles associated with favorable ICI responses could guide personalized treatment approaches in TNBC patients. Furthermore, integrating epigenetic data with genomic and transcriptomic information are expected to provide a more complete understanding of tumor biology and therapeutic resistance mechanisms [186].

The interplay between epigenetic modifications, TMB, and ICI response in TNBC is intricate and rapidly evolving. Unraveling how these epigenetic changes shape tumor behavior and immune responses may pave the way for novel biomarkers and therapeutic strategies, potentially boosting immunotherapy's efficacy in this aggressive breast cancer subtype. Future studies should focus on identifying specific epigenetic changes that are associated with TMB and ICI response. Additionally, scientists should explore therapeutic interventions targeting these epigenetic modifications—a promising avenue for improving patient outcomes.

### Ethnic variations in TMB and ICI responses in TNBC

The exploration of ethnic variations in TMB and ICI therapy response in TNBC is crucial for understanding disparities in cancer outcomes and treatment efficacy. However, variations in TMB across different ethnic groups may influence these responses and necessitate tailored treatment approaches.

Recent studies have indicated that TMB levels can vary significantly among different ethnic populations. For instance, research has shown that African-American women with TNBC tend to have higher TMB compared to their Caucasian counterparts [187]. This finding suggests that genetic predispositions and environmental factors may contribute to these disparities. Furthermore, the prevalence of BRCA mutations, which are associated with increased TMB, also varies by ethnicity, with higher rates observed in certain populations, such as Ashkenazi Jews and individuals of North African descent [188, 189]. These genetic variations could play a role in the observed differences in TMB and subsequent ICI responses.

In terms of ICI response rates, studies have revealed that ethnic disparities exist in the efficacy of these therapies. For example, African-American patients with TNBC have been reported to exhibit lower response rates to ICIs compared to Caucasian patients, despite having higher TMB [190]. This discrepancy may be due to a variety of factors, including differences in tumor microenvironments, immune system responses, and the occurrence of specific genetic mutations that may affect treatment efficacy [191]. The tumor microenvironment plays a crucial role in the efficacy of ICIs. Research indicates that the immune landscape of TNBC can vary significantly between ethnic groups, with African-American patients often exhibiting a more immunosuppressive tumor microenvironment [192]. For instance, the presence of TILs has been correlated with better responses to ICIs in various cancers, yet the levels and functional activity of TILs can differ based on racial and ethnic backgrounds [193, 194]. In TNBC, higher levels of TILs are generally associated with improved outcomes; however, the immune evasion mechanisms employed by tumors in African-American patients may diminish the effectiveness of ICIs [53, 194].

Overall response rates to ICIs in TNBC patients remain low, ranging from 8 to 20% [195]. Certain subgroups, particularly those with high TMB, may benefit more from ICI therapies [196]. However, African-American patients with TNBC experience lower overall survival and progression-free survival rates compared to Caucasian patients, indicating that higher TMB does not necessarily translate to improved therapeutic outcomes [197, 198]. This disparity may be influenced by distinct biological characteristics of TNBC across different racial groups, as African-American women are more likely to present with aggressive disease subtypes associated with poorer prognoses [199, 200].

The observed disparities in TMB levels and ICI responses across ethnic populations in TNBC may be significantly confounded by technical biases inherent in current sequencing methodologies rather than reflecting genuine biological differences. Sequencing platforms commonly used for TMB assessment are susceptible to systematic errors due to underrepresentation of non-European genomes in reference panels, leading to misclassification of germline variants as somatic mutations and artificially inflating TMB estimates in certain populations. Studies demonstrate that tumor-only sequencing approaches overestimate TMB by 2.2-fold in non-European populations compared to 1.5-fold in European populations, with African-American patients experiencing the highest rates of false high-TMB classification (43.6%) compared to Europeans (21%) [201]. This technical artifact results from the predominance of European-derived reference databases; for example, the gnomAD v2.1 reference panel contains 56,885 European exomes but only 8,128 African exomes, creating insufficient filtering capabilities for non-European germline variants [201].

To mitigate these ancestry-related technical biases in TMB estimation, several critical interventions are required: development and implementation of ancestry-specific reference panels with adequate representation of diverse populations, utilization of ancestry-adjusted TMB calibration methods that account for population-specific germline variant frequencies, adoption of population-specific genotype imputation approaches to accurately infer missing genotypes and haplotypes in underrepresented populations [202], deliberate inclusion of diverse cohorts in biomarker validation studies with stratified analyses by genetic ancestry rather than self-reported race.

Additionally, the expression of immune-related biomarkers, such as PD-L1, has been shown to differ across ethnic groups, further complicating the landscape of ICI response [203]. In cohorts from the USA for non-small cell lung cancer (NSCLC) patients, high PD-L1 expression (≥ 50%) was observed in 34% to 55% of tumors with alterations such as ALK and BRAF V600E, whereas lower rates were found in classic EGFR and KRAS mutant tumors [204]. A comprehensive tissue microarray study involving 11,838 tumor samples revealed that PD-L1 positivity was present in 72% of various tumor types globally, but specific ethnic variations were noted within subsets of these cancers [205]. Such discrepancies highlight the significance of considering ethnic backgrounds when evaluating PD-L1 as a predictive biomarker for ICI therapy.

Several genetic and environmental factors may contribute to the observed differences in TMB and ICI response among ethnic groups. Genetic variations, such as single-nucleotide polymorphisms (SNPs) in genes related to DNA repair and immune response, can influence both TMB and the effectiveness of immunotherapy [206]. For instance, certain SNPs, such as rs1024611, rs3740085 have been associated with increased susceptibility to breast cancer and may also affect TMB levels [207, 208]. Environmental factors, including lifestyle, diet, and exposure to carcinogens, can also play an important role in shaping the mutational landscape of tumors [209]. Moreover, socioeconomic factors may impact access to health care and the availability of novel therapies, further exacerbating disparities in treatment outcomes [210].

The implications of these findings for clinical trial design and treatment decisions are profound. It is essential to consider ethnic diversity when designing clinical trials for TNBC treatments, including ICIs. Ensuring that trials are inclusive of various ethnic groups can help elucidate the relative contributions of the genetic versus the environmental factors on treatment responses [211]. Moreover, stratifying patients based on ethnicity and TMB levels may allow for more personalized treatment approaches, optimizing therapeutic efficacy while minimizing adverse effects [212]. Additionally, the development of targeted therapies that address the unique genetic profiles of different ethnic populations could enhance treatment outcomes for TNBC patients [172].

Emerging evidence reveals significant ethnic and population-based disparities in TMB levels, immune microenvironment characteristics, and ICI responses in TNBC that extend beyond socioeconomic factors. Patients of African ancestry demonstrate significantly higher median TMB levels (8.75 muts/Mb) compared to those of Asian ancestry (3.75 muts/Mb), while population-specific patterns show distinct TNBC subtype distributions and immune cell infiltration characteristics across ethnic groups [213]. These biological differences are compounded by significant underrepresentation in clinical research, with approximately 80% of cancer trial participants being white, limiting understanding of how genetic variability affects treatment response and perpetuating health disparities [214]. Furthermore, technical biases in tumor-only sequencing can lead to racially disparate TMB inflation due to underrepresentation in reference databases, potentially resulting in inappropriate treatment selection [39]. The variations extend to differences in neoantigen presentation, HLA allele frequencies, and immune checkpoint protein expression that collectively impact immunotherapy efficacy across populations.

To address these disparities, future clinical trials must prioritize ethnically diverse enrollment and develop ancestry-specific biomarker validation approaches. Such inclusivity is essential not only for health equity but also for scientific rigor, ensuring that TMB-driven prediction models and biomarker thresholds are accurate across all ethnic groups. Enhanced trial design incorporating diverse populations will improve TMB prediction accuracy, optimize personalized therapy selection, and help mitigate systemic disparities while advancing precision oncology for all TNBC patients.

## Data availability and processing methods

### Data sources

#### Description of databases and resources for TMB quantification

Key databases such as The Cancer Genome Atlas (TCGA), the European Genome-phenome Archive (EGA), and the Catalogue of Somatic Mutations in Cancer (COSMIC) provide comprehensive genomic data that are essential for TMB quantification. TCGA, for instance, offers a wealth of genomic and clinical data across various cancer types, including TNBC, allowing researchers to assess TMB relating treatment outcomes [54]. The EGA serves as a repository for high-throughput sequencing data, which can be utilized to derive TMB metrics from patient samples [215]. COSMIC, on the other hand, catalogs somatic mutations and their associated clinical outcomes, serving as a key reference for picturing the mutational landscape of tumors and its implications for immunotherapy [216].

These databases enable researchers to perform large-scale analyses, integrating genomic data with clinical outcomes to identify patterns that may predict ICI efficacy.

#### Importance of large patient cohorts for robust analysis

The analysis of large patient cohorts is critical for validating TMB’s effectiveness as a biomarker for ICI response. Larger sample sizes enhance the statistical power of studies, allowing for more reliable conclusions regarding the relationship between TMB and treatment outcomes. For instance, a systematic review and meta-analysis indicated that elevated TMB correlates with improved efficacy of ICIs (high TMB correlated with significantly enhanced PFS (pooled HR 0.69, 95% CI 0.50–0.95) and OS (pooled HR 0.66, 95% CI 0.47–0.92)) across various cancer types, underscoring the need for robust datasets to substantiate these findings [36].

Moreover, the heterogeneity of tumor responses necessitates the inclusion of diverse patient populations to identify subgroups that may benefit from ICI therapy. In TNBC, where the mutational landscape can vary significantly, large cohorts facilitate the identification of specific genetic alterations that may interact with TMB to influence treatment efficacy [196]. This is particularly relevant as ongoing research aims to refine patient selection criteria for ICIs, potentially leading to more personalized treatment approaches.

In summary, the integration of comprehensive genomic databases and the analysis of large patient cohorts are pivotal in elucidating the role of TMB in predicting ICI responses in TNBC patients. These efforts not only enhance our comprehension of tumor biology but also pave the way for more effective treatment approaches in the management of this challenging variant(s).

### Measuring the TMB value

There is an imperative need to develop a standardized, robust analytical method for measuring TMB values and defining related factors. Proper clinical validation is essential to elevate TMB from a promising biomarker to a clinically validated one.

Measurement methods of TMB include psTMB [217], blood-based TMB (bTMB) obtained from circulating cfDNA [218], targeted NGS panels focused on specific cancer genes [219], and the ecTMB statistical framework [91]. psTMB accounts for potential confounders such as panel size, inherited variants, and technical variability [85]. Targeted NGS panels correlate with WES-based TMB, while ecTMB employs background mutation models to classify tumors into high-TMB, low-TMB, and a novel extreme TMB subtype with prognostic relevance [91]. A summary of available methods is provided in Supplementary Table 2 (Methods for TMB value estimation [Additional File 1]).

**Fig. 3:**
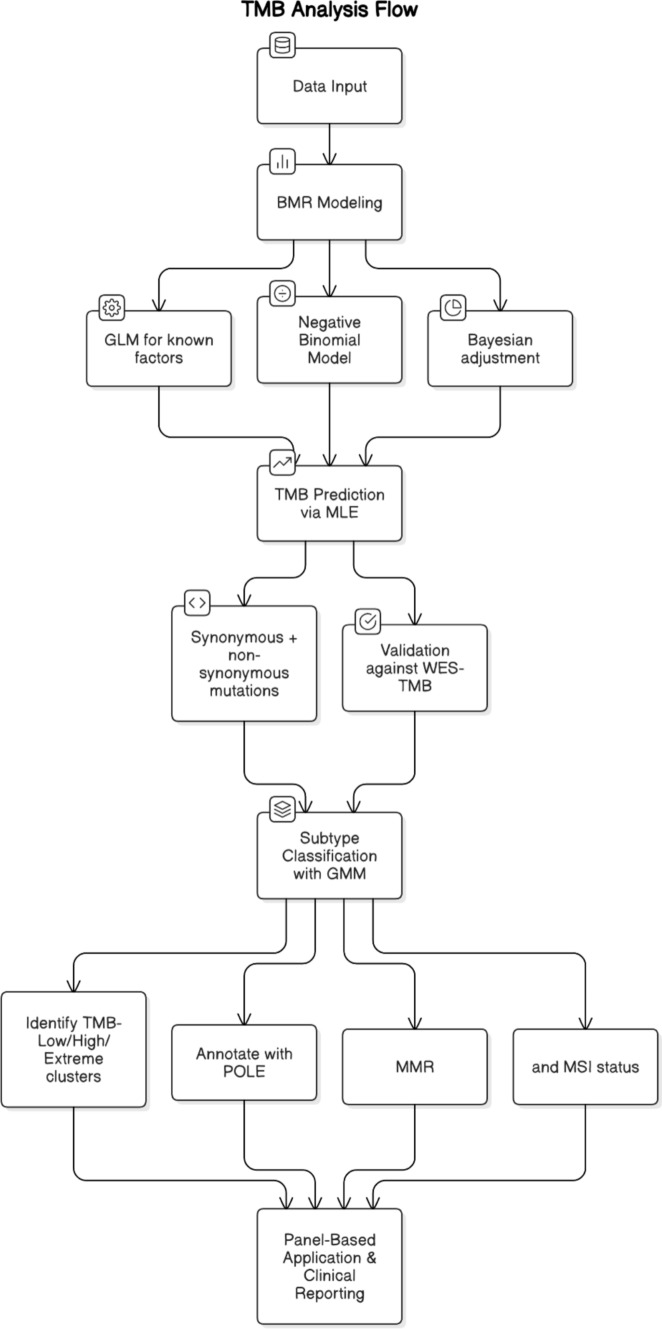
TMB analysis flow. *BMR* Background Mutation Rate, *MLE* Maximum Likelihood Estimation, *GMM *Gaussian Mixture Model, *POLE* DNA Polymerase Epsilon, *MMR* Mismatch Repair, *MSI* Microsatellite Instability

Current approaches include WES and targeted commercial panels [85–89], but a lack of standardization in gene coverage, mutation types, bioinformatics pipelines, and TMB cutoffs limits comparability across studies. WES sequences all protein-coding genes, allowing comprehensive detection of somatic variants such as SNVs and INDELs, and TMB is calculated as non-synonymous mutations per megabase [85]. TMB measurement relies on NGS workflows: DNA extraction from tumor biopsies, library preparation, sequencing, alignment to a reference genome, and variant calling to identify SNVs and INDELs. Targeted panel sequencing may overestimate TMB due to panel design biases. Filtering strategies—such as using COSMIC or incorporating synonymous mutations—can improve alignment with WES-based estimates [90]. Refer Fig. 3 for the TMB analysis flow.

Defining TMB cutoffs remains a major challenge, with thresholds ranging from 10 to 20 mutations/Mb or top decile quantiles. These have predictive value but lack consistency across tumor types and platforms. To address this, Yao et al. proposed the ecTMB framework [91], which uses background mutation models and Gaussian mixture classification to assign tumors into high-TMB, low-TMB, or extreme TMB categories. Compared with simple counting methods, ecTMB improves correlation with WES, reduces errors, and enhances clinical interpretability.

#### Whole-exome sequencing: the gold standard's critical vulnerabilities

*Stromal contamination and low tumor purity* represent the most significant confounders, with studies demonstrating that tumor purity below 30% renders wTMB unreliable for predicting immunotherapy response (log-rank *p* = 0.874 vs. *p* = 0.020 for panel-based approaches) [220]. *DNA input quality* critically affects mutation detection, with improper formalin fixation and poor DNA quality leading to TMB overestimation through artifact detection, while residual germline variants after filtration further compound measurement errors. Additionally, the *cost and turnaround time* of WES (typically > $1000 per sample with 2–3-week processing times) preclude routine clinical use in most healthcare settings.

#### Targeted panel sequencing: systematic biases and design limitations

Panel-based TMB estimation demonstrates systematic biases that inflate or deflate values depending on gene set composition and panel size, with variability increasing proportionally with true WES TMB values. Panel size effects are particularly pronounced, with computational modeling of over 40,000 synthetic panels revealing that panels < 1.04 Mb and < 389 genes fail to achieve 95% discrete accuracy, while panels smaller than 667 kb demonstrate inadequate positive and negative percent agreement across clinically relevant TMB cutoffs [221]. Certain cancer types (uterine, bladder, colon) exhibit two–threefold greater variability compared to lung and head and neck cancers, indicating tumor-type-specific panel performance limitations. *Gene content bias* creates systematic errors, as panels enriched for oncogenes may overestimate TMB due to higher detection rates of pathogenic driver mutations compared to background mutation rates. *Bioinformatics pipeline variations* contribute substantially to measurement discrepancies, with failure to filter pathogenic variants resulting in consistent TMB overestimation across all assays, while optimal germline variant filtering requires balanced precision and recall values (≤ 0.179 reciprocal gap) rather than pursuing "zero false positive" approaches that create measurement imbalances [221].

#### Blood-based TMB: clonal hematopoiesis and detection sensitivity barriers

Blood-based TMB faces unique challenges that severely compromise clinical utility beyond simple convenience. *Clonal hematopoiesis* represents a critical confounder, as age-related clonal mutations in hematopoietic stem cells can be misidentified as tumor-derived variants, artificially inflating bTMB scores particularly in commonly mutated cancer genes such as TP53, KRAS, and JAK2 with prevalence rates of 3–10% in solid tumors [222]. *Circulating DNA fragment quality and detection sensitivity* present fundamental limitations, with contrived reference materials using lung tumor cell lines blended into donor-matched lymphoblastoid cell lines demonstrating that tumor content below 2% yields unreliable TMB estimates using current platforms (PredicineATLAS and GuardantOMNI) [223]. *Maximum somatic allele frequency (MSAF) thresholds* further limit bTMB applicability, with reliable detection requiring MSAF ≥ 1%, effectively excluding up to 30% of cancer patients with lower circulating tumor DNA levels. Correlation between tissue and blood-based TMB improves from *r* = 0.48 to *r* = 0.54 when restricted to samples with MSAF ≥ 1% but remains suboptimal for clinical decision-making [224].

#### Cross-platform harmonization: the greatest barrier to clinical implementation

The lack of standardized measurement protocols represents one of the greatest barriers to clinical TMB adoption, with harmonization challenges extending far beyond simple calibration. Multi-laboratory studies reveal that while 87.7% of pairwise panel comparisons achieve correlation coefficients > 0.6, this seemingly acceptable correlation masks clinically significant misclassification rates that would result in inappropriate treatment decisions [225]. *Bridging factor complexity* demonstrates the depth of harmonization challenges, with wTMB cutpoints of 199 mutations projecting to panel-specific cutpoints ranging from 7.8 to 12.6 mutations/Mb, achieving only 74.9% classification agreement even after mathematical adjustment [225]. Comparative analyses consistently demonstrate that TMB values are systematically higher in panel cohorts compared to WES cohorts, with average mutation rates per gene showing high concordance across methodologies (Pearson's correlation coefficients ranging from 0.842 to 0.866) [59]. *Statistical calibration approaches*, while developed to achieve more consistent results across panels, require extensive validation datasets and complex computational frameworks that most clinical laboratories cannot implement independently. The *Z-score conversion methods* represent promising approaches for harmonizing TMB values across platforms, but their clinical implementation requires standardized reference datasets that currently do not exist across diverse tumor types and populations.

#### Quality assessment and external validation limitations

Current harmonization initiatives, while commendable, remain fundamentally insufficient for reliable clinical implementation across diverse healthcare systems. The International Quality Network for Pathology survey revealed that 127 laboratories employ multiple different panels with variable mutation counting criteria, creating a landscape of measurement heterogeneity that undermines result interpretation [87]. *External quality assessment programs* are in early developmental stages, with the Friends of Cancer Research TMB Harmonization Project and Quality in Pathology initiatives providing frameworks but not yet achieving the standardization necessary for regulatory approval across diverse healthcare systems. *Variant allele frequency thresholds* vary substantially across platforms (1–10%), with optimal thresholds being tumor purity-dependent (5% VAF suitable only for samples with ≥ 20% tumor purity), further complicating standardization efforts [221]. Batch effects, arising from differences in sample preparation, sequencing reagents, or instrumentation, present another source of technical variability and can hinder meaningful comparisons, especially in multi-institutional studies. Tumor purity and DNA extraction techniques are also important confounders—samples with lower tumor content or degraded DNA are prone to underestimation of the true mutational burden, regardless of sequencing depth or strategy.

These technical limitations collectively demonstrate that TMB measurement represents not merely an analytical challenge but a fundamental barrier to precision medicine implementation that requires sustained, coordinated international efforts to achieve clinical reliability. The coefficient of variation in panel-based TMB decreases at a rate inversely proportional to the square root of both panel size and TMB level, indicating that precision is tumor-specific and cannot be universally optimized. Inter- and intra-tumor heterogeneity compounds these challenges, as TMB from metastatic sites can differ significantly from primary sites due to varying clonal evolution, yet this disparity may not correlate with immunotherapy benefit. Most critically, no universal cutoff exists to define "high" versus "low" TMB, with studies using thresholds of 10–20 muts/Mb that vary by tumor type, population, and platform, fundamentally undermining clinical interpretability across healthcare systems.

### Functional analyses establishing the link between TMB and ICI treatment prediction

Experimental designs in cancer research have evolved significantly, particularly with the advent of three-dimensional (3D) coculture models. These models are crucial for mimicking the TME more accurately than traditional two-dimensional (2D) cultures. The intrinsic complexity of tumors necessitates the use of homotypic and/or heterotypic coculture systems that incorporate a wide array of cellular types, including tumorigenic cells, stromal cells, and immune cells, to accurately model the interactions and dynamics present in vivo [226, 227].

One notable advancement is the development of 3D coculture spheroidal models, which enhance the representation of tumor complexity and allow for twofold analysis of tumor growth and angiogenesis [228]. These models facilitate the study of T cell penetration and the efficacy of anti-tumoral compounds, providing insights into the immune response to cancer therapies [229]. The integration of immune cells, such as T lymphocytes, into these models is particularly relevant for assessing ICIs, which are designed to enhance the immune system's capacity to target and eliminate tumorgenic cells [227, 230].

Experimental techniques for assessing ICI-mediated treatment responses in 3D coculture models include immunohistochemistry (IHC) and single-cell RNA sequencing. IHC allows for the visualization of specific immune cell populations and their interactions with tumor cells within the 3D architecture, while single-cell RNA sequencing may provide a better understanding of the heterogeneity contributing to variations in the molecular landscape, revealing how TMB correlates with immune cell activation and response to therapy [227, 231].

Moreover, the use of organoid models has emerged as an approach, with tremendous potential, for studying the TME and evaluating treatment responses [232]. Organoids can be derived from patient tumors and maintained in culture, allowing for personalized assessments of how specific mutations influence ICI efficacy [233, 234]. This personalized approach is particularly valuable in TNBC, where heterogeneity in TMB and immune landscape can significantly affect treatment outcomes.

The integration of 3D coculture models and cutting-edge immune response assessment techniques offers a powerful framework for unraveling the complex interplay between TMB and ICI therapy in TNBC. These sophisticated models not only bolster the predictive value of preclinical studies but also blaze a trail toward more tailored, effective cancer therapies. By closely mimicking the tumor microenvironment, they provide invaluable insights that bridge the gap between laboratory findings and clinical applications, potentially revolutionizing personalized treatment strategies for TNBC patients.

## Future?

### Limitations of current research

TMB is emerging as a promising biomarker for immunotherapy response, notwithstanding several challenges affecting its clinical implementation. Variability in TMB measurement methods across assays and centers necessitates standardization [235]. While WES is the gold standard, targeted gene panels are more practical for clinical use [236]. However, TMB quantification based on panel-based measurements is influenced by factors such as panel size, composition, and bioinformatic parameters [237]. Pre-analytical factors, including DNA quality and tumor purity, can affect TMB estimation [238]. The lack of harmonization in panel-based TMB quantification and robust predictive cutoffs limits its adoption in clinical practice [236]. Integration with other biomarkers may enhance TMB's predictive capabilities [237]. Ongoing efforts by organizations like Friends of Cancer Research and Quality Assurance Initiative Pathology aim to establish consistent TMB assessment methods across platforms [237].

The relationship between TMB and the ICI responses has garnered significant attention in recent years. However, current research reveals several limitations in accurately assessing this relationship, particularly concerning the variability in TMB measurement and the challenges in standardizing TMB cutoff values for patient stratification.

One of the primary limitations of TMB research is the variability in measurement techniques. Different NGS panels can yield divergent TMB results, complicating the interpretation of TMB as a surrogate measure for ICI response. For instance, a study highlighted that the assessment of TMB using various targeted sequencing panels demonstrated significant discrepancies, indicating that correlation alone is insufficient for comparing these panels [239]. This variability can lead to inconsistent clinical decisions, as the same tumor may be classified differently depending on the sequencing method employed [86]. Furthermore, the harmonization of TMB assessment across different platforms remains a challenge, as evidenced by the Friends of Cancer Research TMB Harmonization Project, which aimed to establish guidelines for TMB quantification but revealed substantial variation in results across diagnostic platforms [46].

Current research indicates that the predictive value of TMB as a biomarker for ICI response is still under investigation, with no universally accepted cutoff values for patient stratification [240]. Studies have suggested that higher TMB cutoffs may better identify patients who are exceptional responders to ICIs, yet the reproducibility of these cutoffs across different cancer types has not been thoroughly validated [241, 242]. The continuous nature of TMB as a numeric variable further complicates the establishment of discrete cutoff values, as it necessitates a nuanced understanding of how varying levels of TMB correlate with treatment outcomes [243].

The clinical implications of TMB cutoff values are profound, directly influencing patient eligibility for ICI therapies. The lack of consensus on optimal thresholds can lead to either overtreatment or under-treatment. Some patients with lower TMB may respond favorably to ICIs, while others with high TMB may not benefit [244]. This inconsistency highlights the urgent need for large-scale studies to validate TMB thresholds and enhance the robustness of risk stratification systems [245].

### Future research directions

Recent research underscores both the promise and challenges of adopting TMB as a biomarker for immunotherapeutic response in TNBC. While TMB correlates with neoantigen burden and T cell infiltration in many solid tumors, its role in breast cancer remains ambiguous [100]. Standardizing TMB assessment across tumor types and platforms is vital for consistency [244]. Previous research has established robust concordance between panel sequencing-derived and WES-derived TMB measurements, highlighting their complementary clinical utility and potential to enhance experimental reproducibility. Combining TMB with other biomarkers, such as PD-L1 expression on activated immune cells—specifically DCs and effector CD4^+^ T cells (TEFFs)—alongside the TIL quantification with functional markers (e.g., 4-1BB, PD-1), could boost its predictive power [246]. Future studies should prioritize establishing optimal TMB cutoffs and developing robust genomic tools for its determination [100, 247–249]. The interplay between TMB and other genomic features, such as mutational profiles in other genes (PD-L1 expression and TILs), has been shown to have a bearing on the overall survival and response to ICI therapy [53, 246, 250]. Studies reveal that PD-1 is predominantly expressed on exhausted CD8^+^ and CD4^+^ TILs marked by co-expression of inhibitory receptors (e.g., CD39, TIM-3) and reduced cytokine production.

In head and neck cancers, PD-1^hi^ CD39^+^ CD4^+^ TILs exhibit terminal exhaustion but retain tumor antigen specificity and can be reactivated by PD-1 blockade [251]. In NSCLC, MANA-specific CD8^+^ TILs show high PD-1 expression alongside other checkpoints (e.g., CTLA-4, TIGIT), correlating with incomplete cytolytic activation and resistance to ICIs [252].

This heterogeneity suggests that selectively targeting exhausted TIL subsets through PD-1 editing may reverse their dysfunction. Although there are presently no published clinical trials for TNBC utilizing this approach, existing mechanistic studies support its feasibility [253]. Isolating PD-1^+^ TILs via flow cytometry or magnetic sorting could enrich tumor-reactive clones [254]. Established ex vivo expansion protocols, such as those utilizing IL-2 and IL-15 stimulation, could be adapted for TNBC [255, 256]. The use of CRISPR-Cas9 or TALEN technology has shown over 80% efficiency in disrupting PD-1 in TILs [253]. A study, performed recently, involving ROR1 CAR-T cells with inducible anti-PD-1 single-chain variable fragments (scFv) in TNBC models demonstrated enhanced tumor control, indicating a potential synergistic effect between PD-1 blockade and engineered T cells [253].

Moreover, precision medicine approaches, including molecular subtyping and genomic profiling, show promise for TNBC treatment but face hurdles due to tumor heterogeneity and clonal evolution [127]. Ongoing global collaborative efforts in research and clinical validation are crucial to fully harness TMB's potential as a biomarker in TNBC [244].

Future research directions regarding TMB in TNBC should focus on several key areas to elucidate its role as a biomarker that has potential that is predictive of immunotherapy. First, prospective clinical trials are essential to validate TMB as a reliable biomarker in TNBC, particularly in the context of ICIs. These studies should aim to establish standardized TMB cutoff values specific to TNBC, as current thresholds are often derived from other cancer types and may not be applicable [100, 244].

Additionally, integrating TMB into clinical trials alongside other biomarkers, such as PD-L1 expression, could enhance patient stratification and treatment personalization. Research has shown that combining TMB with other genomic features can improve predictive accuracy for ICI response [257]. Furthermore, the exploration of TMB in conjunction with other molecular characteristics, such as copy number alterations (CNA), may provide deeper insights into the TME and its influence on treatment outcomes [258].

Skeletal metastases of unknown primary (SMUP) illustrate a parallel to TNBC in that both clinical contexts demand integration of tumor‐intrinsic biomarkers with microenvironmental cues rather than reliance on single modalities alone. In SMUP, bisphosphonates and bone turnover markers are employed not only to support bone health but also to modulate the osteoblast–osteoclast equilibrium within the bone niche, thereby influencing metastatic outgrowth and patient outcomes [259]. Bone turnover markers such as serum C‐terminal telopeptide (CTX) and procollagen type I N‐terminal propeptide (PINP) serve as dynamic indicators of this microenvironmental balance and guide therapeutic timing and intensity. Analogously, in TNBC, treatment efficacy hinges on modulating the immune microenvironment through biomarkers like TMB, PD‐L1 expression, and TIL density, which reflect the equilibrium between effector and suppressor cell populations. Both scenarios underscore the necessity of combining molecular biomarkers—whether TMB or bone‐specific markers, with context‐specific microenvironmental signals to design therapies tailored to the dynamic interplay between cancer cells and their surrounding stroma. This integrative approach, demonstrated in SMUP management, provides a valuable framework for advancing patient‐centered immunotherapeutic strategies in TNBC.

Finally, the development of robust bioinformatics tools and harmonization of TMB assessment methods across different platforms will be crucial for the widespread clinical application of TMB as a biomarker in precision medicine [238, 260]. This will ensure that TMB can be effectively utilized in clinical settings, ultimately improving therapeutic strategies for patients with TNBC.

#### TMB-guided integration with novel therapeutic modalities

The predictive value of TMB extends beyond traditional ICI selection to guide integration with emerging therapeutic strategies that can enhance immune activation and overcome resistance mechanisms. *Biomimetic drug delivery systems*, such as leukosomes—leukocyte membrane-coated nanoparticles—represent a promising approach to improve ICI delivery specifically to the tumor microenvironment [261]. In TNBC patients with high TMB, leukosomes could potentially enhance anti-PD-L1 antibody accumulation within immunogenic tumors, maximizing therapeutic efficacy while minimizing systemic toxicity. The natural tropism of leukocytes for inflamed tissues makes this delivery strategy particularly relevant for high-TMB tumors that typically exhibit robust immune infiltration.

*MicroRNA-based gene therapy* presents a complementary strategy to TMB-guided immunotherapy. Specific microRNAs play a crucial role in modulating immune resistance mechanisms, thereby enhancing the sensitivity of tumors to ICIs. By influencing the immunogenicity of tumor cells and the function of immune cells, certain microRNAs can facilitate a more potent immune response. For instance, targeting immunosuppressive microRNAs in high-TMB TNBC could amplify the benefits of neoantigen presentation, synergizing with checkpoint blockade [262, 263]. Conversely, in low-TMB tumors, microRNA therapeutics might enhance immunogenicity, preparing the tumor microenvironment for subsequent ICI therapy [262, 264].

Emerging studies highlight the potential of microRNAs to modulate immune responses and their implications for treatment specificity across various tumor types [264, 265]. A thorough exploration and integration of microRNA therapies into current immunotherapy frameworks stand to improve patient outcomes, particularly in those with diverse tumor immunogenic profiles [265, 266]. This approach underscores the importance of targeted research into microRNA interactions within different tumor microenvironments to develop effective treatment strategies that address current limitations [262, 267].

The integration of reproducible, globally accepted TMB assessment methods with these novel cutting-edge omics-based modalities represents a paradigmatic shift toward precision immunotherapy, where biomarker-guided stratification may aid in better patient selection thereby optimizing drug(s)-delivery strategy combinations. Future clinical trials should investigate TMB thresholds for optimal biomimetic carrier utilization and microRNA therapeutic targeting to maximize the translational impact of precision oncology approaches in TNBC.

### Combination therapies and TMB in TNBC

The exploration of combination therapies in the treatment of TNBC has gained significant attention, particularly with the advent of ICIs and their potential synergistic effects when combined with other therapeutic modalities such as chemotherapy and PARP inhibitors (Refer the Table 3). TNBCs present unique challenges in treatment due to its aggressive nature and higher relapse rates compared to other subtypes of breast cancer [275]. The integration of ICIs into treatment regimens aims to increase the anti-tumorigenic immune response, thereby improving patient outcomes.

**Table 3:** Combination therapies with ICI

Therapy type	Combination	Mechanism	Trial phase/model	Key outcomes	References
Vaccination + ICI	HPV-16 peptide vaccine + nivolumab	Enhances antigen-specific CD8^+^ T-cell infiltration	Phase II	33% ORR in HPV16^+^ cancers	[268–270]
	CpG oligodeoxynucleotides (TLR9 agonist) + PD-1i	Activates plasmacytoid DCs → IFN-α/β → T-cell recruitment	Preclinical (TNBC)	Superior tumor regression in TNBC models	[270–272]
	Neoantigen vaccines + ICI	Boosts T-cell responses in low-mutational burden tumors	Preclinical	Enhanced tumor control in melanoma and NSCLC	[270, 273]
Epigenetic Modulators + ICI	HDAC inhibitors (e.g., entinostat) + anti-PD-1	↑ PD-L1 expression, T-cell infiltration	Phase I/II (TNBC)	Improved ORR in PD-L1^+^ TNBC	[270, 272, 273]
	DNMT inhibitors (azacitidine) + anti-PD-1	Demethylates DNA → reactivates silenced immune genes	Phase II	Enhanced response in "cold" tumors	[270, 273]
	EZH2 inhibitors + CTLA-4/PD-1 blockade	↓ T_regs_, ↑ effector T-cell activity	Preclinical	Synergy in TNBC and melanoma models	[272, 273]
	SETDB1 inhibitors + ICIs	Block SETDB1 (H3K9 methyltransferase) → ↑ MHC-I antigen presentation via TE-derived antigens → ↑ CD8^+^ T-cell infiltration	Preclinical	Enhanced tumor control in ICB-treated models; induces TE-encoded MHC-I peptides	[273]
Natural Molecules + ICI (TNBC)	Ginsenoside Rg3 + paclitaxel + PD-1i	Inhibits TGF-β/NF-κB → chemosensitization	Preclinical	↑ Cytotoxicity, ↓ metastasis	[271, 274]
	Anacardic acid (6SA) + PD-1i	Induces caspase-8 apoptosis; ↑ IFN-γ/TNF-α	Preclinical (TNBC)	Improved immune microenvironment	[271, 274]
	Gut microbiota metabolites (TMAO) + ICI	Enriches *Clostridium* → ↑ CD8^+^ T cells	Phase I (TNBC)	Correlation with improved ICI response; trials testing choline supplementation	[271, 272, 274]
Signal Transduction + ICI	TIGIT inhibitor (domvanalimab) + PD-1i	Blocks T-cell exhaustion	Phase II (ARC-7)	↑ PFS in PD-L1-high NSCLC	[270, 272]
	TGF-β trap (PD-L1/TGF-β bispecific) + ICI	Remodels TME → ↑ CD8^+^ T cells	Phase I/II (TNBC)	Tumor regression in TNBC and urothelial carcinoma	[272]
	OX40 agonist (GSK3174998) + pembrolizumab	Activates T cells → tumor regression	Phase I	Partial responses in solid tumors	[270]
	4-1BB (CD137) agonists + PD-1/PD-L1 inhibitors	Activates 4-1BB costimulatory signaling → ↑ cytotoxic T-cell proliferation	Early-phase trials	Enhanced T-cell activation and tumor regression in preclinical models; trials ongoing in solid tumors	[272]
Radiotherapy/Procedural + ICI	Cryoablation + ipilimumab/nivolumab	Releases tumor antigens → enhances T-cell priming	Phase II (TNBC) (NCT03546686)	Early efficacy signals in early-stage TNBC	[272]
	Pembrolizumab + radiotherapy	↑ Immunogenic cell death → synergizes with PD-1 blockade	Phase II (NCT04443348)	Ongoing trial in TNBC	[272]
Microbiome Modulation	FMT/Probiotics + ICI	Enriches *Akkermansia* → ↑ ICI efficacy	Phase II	Improved response rates in melanoma and TNBC	[270, 274]

ORR, Objective Response Rate; PFS, Progression-Free Survival; ↑, Heightened/high; ↓, Low/suppressed

Recent studies have shown that combining ICIs with chemotherapy can lead to improved efficacy in various malignancies, including TNBC. For instance, PD-1/PD-L1 inhibitor combinations with chemotherapy have been shown to increase survival outcomes (OS and PFS) in patients with advanced NSCLC [276, 277]. The association between TMB and treatment outcomes extends beyond TNBC to other cancer types, including those involving the colorectal, lung, and gastric regions [278–280].

This approach is particularly relevant for TNBC, where traditional chemotherapy continues to be a primary modality. The synergistic effect of chemotherapy may enhance the immunogenicity of tumor cells, thereby making them more susceptible to ICI therapy [188]. Furthermore, the inclusion of ICIs to chemotherapy regimens has been associated with a favorable safety profile, although the appearance of irAEs can increase [281, 282].

PARP inhibitors have also emerged as a hopeful therapeutic option for TNBC, especially in patients with BRCA1/2 mutations or homologous recombination deficiency (HRD). The mechanism of action of PARP inhibitors involves the induction of synthetic lethality in cancer cells with defective DNA repair pathways [283]. When combined with ICIs, PARP inhibitors may augment the immune response by modulating the TME and promoting T cell infiltration into tumors. This combination strategy is supported by evidence suggesting that PARP inhibitors can increase PD-L1 expression on tumor cells, potentially enhancing the efficacy of ICIs [284].

The utility of TMB as a predictive biomarker in the context of combination therapies is gaining traction. TMB, which quantifies the number of mutations within a tumor, has been associated with improved therapeutic outcomes to ICIs across various cancer types, including lung cancer and melanoma [160, 285]. In TNBC, a higher TMB may correlate with better outcomes when treated with ICIs, particularly in combination with chemotherapy or PARP inhibitors [160]. This relationship underscores the importance of TMB as a potential stratification tool for selecting patients who are most likely to benefit from combination therapies.

Moreover, the interplay between TMB and the efficacy of combination therapies raises intriguing possibilities for personalized medicine in TNBC. Patients with high TMB may experience enhanced benefits from ICIs due to the increased likelihood of neoantigen formation, which can elicit a robust immune response [160]. Conversely, those with low TMB may require alternative strategies or additional therapeutic agents to achieve similar outcomes. This necessitates further research to elucidate the optimal combinations and treatment sequences based on TMB and other molecular characteristics. Also, since TMB measurements need not necessarily correlate with ICI response in all TNBC patients, further model system-based studies are warranted to dissect and better understand the mechanisms involved, before this approach can be adopted in routine clinical practice as a viable approach for personalizing anti-tumor therapy.

The safety profile of combination therapies involving ICIs, chemotherapy, and PARP inhibitors is an essential consideration in clinical practice. While the combination of ICIs with chemotherapy has shown improved efficacy, it is also associated with an increased risk of mild to severe irAEs [188, 282]. The management of these adverse events is crucial to ensure that patients can continue their treatment without significant interruptions. Additionally, PARP inhibitors and ICI combinations may also present unique safety challenges, necessitating careful monitoring and management strategies [286].

## Perspectives: toward integrated biomarker-driven precision medicine in TNBC

The ultimate goal in TNBC management extends beyond refining individual biomarkers to developing integrated models of personalized medicine that synthesize multiple layers of tumor and host information. The future of TNBC treatment lies in creating comprehensive patient stratification frameworks that combine TMB quantification with complementary biomarkers and innovative therapeutic delivery systems.

### Multi-dimensional patient stratification

An integrated approach to TNBC precision medicine should incorporate three critical dimensions: *1. PD-L1 expression profiling* to assess baseline immune activation status, *2. TMB quantification* to evaluate neoantigen load and immunogenic potential, and *3. Immune phenotyping at the single-cell level* to characterize the heterogeneous tumor microenvironment architecture. This tripartite classification system would enable clinicians to identify distinct TNBC subgroups: immunologically "hot" tumors with high PD-L1, high TMB, and robust immune infiltration; "cold" tumors with low expression across all parameters; and intermediate phenotypes providing opportunities for tailored therapeutic approaches.

### Integration with novel delivery and targeting strategies

The clinical implementation of this integrated model should incorporate next-generation therapeutic modalities. Leukosomes and other biomimetic carriers can enhance drug delivery efficiency in immunologically active, high-TMB tumors, while epigenetic modulators targeting microRNAs can reprogram immune resistance mechanisms in low-TMB cases. This biomarker-guided approach to delivery system selection represents a fundamental advance toward truly personalized cancer therapy.

### Toward biologically rational combination therapies

The convergence of biomarker science and therapeutic innovation enables the development of combination therapies that are both biologically rational and clinically feasible. For example, patients with high TMB and robust immune infiltration might benefit from enhanced ICI delivery via biomimetic carriers, while those with low TMB could receive microRNA-based immune priming followed by conventional immunotherapy. Such stratified combination approaches promise to expand the population of TNBC patients who benefit from immunotherapy while optimizing treatment sequencing and minimizing unnecessary toxicity.

This vision of integrated precision medicine represents the next frontier in TNBC management, where biomarker assessment drives not only therapeutic selection but also delivery optimization and combination strategy design.

## Ethical considerations and clinical implementation challenges

While TMB has generated enthusiasm as a predictive biomarker, its clinical application remains constrained by methodological limitations and ethical concerns. Evidence from cancer biomarker studies demonstrates a concerning pattern of overinterpretation and premature clinical implementation, which can expose patients to ineffective or potentially harmful therapies.

The substantial inter-assay variability in TMB measurement presents significant ethical concerns when translated to clinical practice. Different NGS platforms yield divergent TMB values for identical samples, with correlations between targeted panels and WES ranging from 0.68 to 0.81 [19]. This technical variability could result in misclassification of patients, potentially denying effective therapy to those who might benefit or subjecting others to ineffective treatments with associated toxicities. The lack of standardized, TNBC-specific cutoff values further compounds these risks, as current thresholds are largely derived from other cancer types and may not translate appropriately to the unique biology of TNBC.

High TMB does not consistently predict ICI response, as outcomes are influenced by immune evasion, tumor microenvironment, and patient-specific factors. This highlights the insufficiency of single biomarkers in guiding therapy. Ethical implementation demands prospective validation in diverse TNBC cohorts, harmonized measurement protocols, TNBC-specific thresholds, and integration with complementary biomarkers. Clinical translation should prioritize patient safety, informed consent, and rigorous trial safeguards. Multidisciplinary collaboration is essential to ensure both scientific validity and ethical integrity in TMB-guided strategies. In acknowledging the inherent uncertainties of science—particularly in the emerging field of biomarkers—Kern [287] emphasizes that recognizing past failures is not merely cautionary but constructive. Such recognition highlights the biological, technical, and conceptual complexities underlying biomarker development and may foster more judicious allocation of limited research resources.

## Conclusions

The measurement of TMB is a continuous variable that exhibits variability both across different cancer types and within specific malignancies. Certain cancers, such as lung, and head and neck cancers, display relatively consistent TMB levels. These biomarkers have been documented to be important for predicting immunotherapy outcomes in certain other cancers as well (e.g., melanoma) [288–290]. However, other cancer types demonstrate significant fluctuations in TMB. Although TMB holds promise as a predictive biomarker for immunotherapy response, further validation across diverse cancer types (temporal and/or spatial variation) and treatment regimens are essential to establish its clinical utility in guiding therapeutic decisions.

While TMB represents a promising investigational tool for understanding immunotherapy responses in TNBC, the current evidence base contains substantial limitations that preclude its immediate adoption as a clinical decision-making biomarker. The methodological heterogeneity in TMB measurement, lack of standardized TNBC-specific cutoffs, and limited validation in diverse populations necessitate continued research within carefully controlled trial settings. Future studies must prioritize rigorous large-scale, prospective clinical trials with composite biomarker panels including harmonized cross-platform validation approaches, multi-omic integration (better comprehension of the mechanisms involved in TMB-ICI responses), and ethical considerations. This combinatorial approach will ensure that biomarker development and a thorough analysis of their interactions serve patient welfare rather than perpetuating premature clinical translation. As a final concluding statement, TMB should be considered as part of a comprehensive biomarker panel rather than a singular predictive tool, with clinical implementation contingent upon a continual, robust, corroborative, iterative validation through systematic reviews (existing and new data) employing advanced statistical methodologies and meta-analytic approaches that account for the substantial heterogeneity observed across assays, populations, and therapeutic regimens in TNBC immunotherapy trials. The path forward requires scientific humility, acknowledging the complexity of TNBC biology while maintaining commitment to evidence-based medicine that prioritizes patient safety and equitable access to validated therapeutics.

## Supplementary Information

Below is the link to the electronic supplementary material.

Supplementary file 1 (DOCX 65 kb)

## Data Availability

All data that underpin the findings of this study can be found within the main text of the paper and its Supplementary Information.

## Abbreviations

TNBC: Triple-Negative Breast Cancer
ER: Estrogen Receptor
PR: Progesterone Receptor
HER2: Human Epidermal Growth Factor Receptor 2
ICI: Immune Checkpoint Inhibitor
TMB: Tumor Mutational Burden
PARP: Poly(ADP-ribose) Polymerase
BRCA1/2: Breast Cancer gene 1 and 2
DCIS: Ductal Carcinoma in situ
PD-1: Programmed Cell Death Protein 1
PD-L1: Programmed Cell Death Ligand 1
CTLA-4: Cytotoxic T-Lymphocyte Antigen 4
TME: Tumor Microenvironment
NGS: Next-Generation Sequencing
psTMB: Panel Sequencing-based Tumor Mutational Burden
IM TNBC: Immune Modulated Triple-Negative Breast Cancer
M-TNBC: Mesenchymal Subtype TNBC
MSL-TNBC: Mesenchymal Stem-Like TNBC
LAR-TNBC: Luminal Androgen Receptor TNBC
UNS-TNBC: Unstable Subtype TNBC
FFPE: Formalin-Fixed Paraffin-Embedded
OTML: Oncotype DX Test for Multigene Assay
TIL: Tumor-Infiltrating Lymphocytes
MSI: Microsatellite Instability
MMR: Mismatch Repair
MHC: Major Histocompatibility Complex
DC: Dendritic Cell
DST: Drug Sensitivity Testing
DDR: DNA Damage Response
EGFR: Epidermal Growth Factor Receptor
ADC: Antibody Drug Conjugate
irAE: Immune-related Adverse Events
PTEN: Phosphatase and Tensin Homolog
PI3K: Phosphoinositide 3-Kinase
TAM: Tumor Associated Macrophage
MDSC: Myeloid-Derived Suppressor Cell
M-MDSC: Monocytic Myeloid-Derived Suppressor Cell
G-MDSC: Granulocytic Myeloid-Derived Suppressor Cell
CAM: Chick Embryo Chorioallantoic Membrane Assay
HDACi: Histone Deacetylase Inhibitor
NSCLC: Non-Small Cell Lung Cancer
SNP: Single-Nucleotide Polymorphism
TCGA: The Cancer Genome Atlas
EGA: European Genome Archive
COSMIC: Catalogue of Somatic Mutations in Cancer
bTMB: Blood-based Tumor Mutational Burden
ecTMB: Estimation and Classification of TMB
WES: Whole-Exome Sequencing
SNV: Single-Nucleotide Variant
INDEL: Insertion and Deletion
IHC: Immunohistochemistry
TEFF: Effector CD4^+^ T cells
HRD: Homologous Recombination Deficiency

## References

[1] Howard FM, Olopade OI. Epidemiology of triple-negative breast cancer. Cancer J. 2021;27(1):8–16. 10.1097/PPO.0000000000000500. 33475288 10.1097/PPO.0000000000000500 PMC12050094

[2] Verma R, Lal Jakhar S, Sharma N, Kumar HS, Beniwal S. Epidemiological profile and clinicopathological correlates of triple negative breast cancer patients at regional cancer centre. Asian Pac J Cancer Care. 2021;6(4):457–60.

[3] Suvobrata S, Murtaza A. Triple negative breast cancer prevalence in indian patients over a decade: a systematic review. Int J Clin Biostat Biom. 2022;8(1):045.

[4] Du X. Racial disparities in health insurance, triple-negative breast cancer diagnosis, tumor stage, treatment and survival in a large nationwide SEER cohort in the United States. Mol Clin Oncol. 2022;16(4):95. 35368847 10.3892/mco.2022.2528 PMC8943535

[5] Anders CK, Carey LA. Biology, metastatic patterns, and treatment of patients with triple-negative breast cancer. Clin Breast Cancer. 2009;9:S73-81. 19596646 10.3816/CBC.2009.s.008 PMC2919761

[6] Caglevic C, Anabalón J, Soza C, Milla E, Gaete F, Carrasco AM, et al. Triple-negative breast cancer: the reality in Chile and in Latin America. Ecancermedicalscience. 2019. 10.3332/ecancer.2019.893. 30792810 10.3332/ecancer.2019.893 PMC6372297

[7] Metcalfe KA, Narod SA, Eisen A, Poll A, Zamani N, McCready D, et al. Genetic testing women with newly diagnosed breast cancer: what criteria are the most predictive of a positive test? Cancer Med. 2023;12(6):7580–7. 10.1002/cam4.5515. 36544278 10.1002/cam4.5515 PMC10067031

[8] Phipps AI, Buist DSM, Malone KE, Barlow WE, Porter PL, Kerlikowske K, et al. Family history of breast cancer in first-degree relatives and triple-negative breast cancer risk. Breast Cancer Res Treat. 2011;126(3):671–8. 10.1007/s10549-010-1148-9. 20814817 10.1007/s10549-010-1148-9 PMC3059326

[9] Tiwari P, Blank A, Cui C, Schoenfelt KQ, Zhou G, Xu Y, et al. Metabolically activated adipose tissue macrophages link obesity to triple-negative breast cancer. J Exp Med. 2019;216(6):1345–58. 31053611 10.1084/jem.20181616 PMC6547867

[10] Maiti B, Kundranda MN, Spiro TP, Daw HA. The association of metabolic syndrome with triple-negative breast cancer. Breast Cancer Res Treat. 2010;121(2):479–83. 10.1007/s10549-009-0591-y. 19851862 10.1007/s10549-009-0591-y

[11] Farshbafnadi M, Pastaki Khoshbin A, Rezaei N. Immune checkpoint inhibitors for triple-negative breast cancer: from immunological mechanisms to clinical evidence. Int Immunopharmacol. 2021;98:107876. 34146865 10.1016/j.intimp.2021.107876

[12] Steiner M, Tan AR. The evolving role of immune checkpoint inhibitors in the treatment of triple-negative breast cancer. Clin Adv Hematol Oncol. 2021;19(5):305–15. 33989278

[13] Zhu S, Wu Y, Song B, Yi M, Yan Y, Mei Q, et al. Recent advances in targeted strategies for triple-negative breast cancer. J Hematol Oncol. 2023;16(1):100. 10.1186/s13045-023-01497-3. 37641116 10.1186/s13045-023-01497-3 PMC10464091

[14] Tarekegn K, Keskinkilic M, Kristoff TJ, Evans ST, Kalinsky K. The role of immune checkpoint inhibition in triple negative breast cancer. Expert Rev Anticancer Ther. 2023;23(10):1095–106. 10.1080/14737140.2023.2265059. 37771270 10.1080/14737140.2023.2265059

[15] Zhao Z, Zhang S, Jiang N, Zhu W, Song D, Liu S, et al. Patient-derived immunocompetent tumor organoids: a platform for chemotherapy evaluation in the context of t-cell recognition. Angew Chemie. 2024. 10.1002/ange.202317613. 10.1002/anie.202317613 38195970

[16] Funes SC, de Manrique Lara A, Altamirano-Lagos MJ, Mackern-Oberti JP, Escobar-Vera J, Kalergis AM. Immune checkpoints and the regulation of tolerogenicity in dendritic cells: implications for autoimmunity and immunotherapy. Autoimmun Rev. 2019;18(4):359–68. 30738957 10.1016/j.autrev.2019.02.006

[17] Shiravand Y, Khodadadi F, Kashani SMA, Hosseini-Fard SR, Hosseini S, Sadeghirad H, et al. Immune checkpoint inhibitors in cancer therapy. Curr Oncol. 2022;29(5):3044–60. 35621637 10.3390/curroncol29050247 PMC9139602

[18] Guo ZS. The 2018 Nobel Prize in medicine goes to cancer immunotherapy (editorial for BMC cancer). BMC Cancer. 2018;18:1086. 30415640 10.1186/s12885-018-5020-3 PMC6231274

[19] Xiong N, Wu H, Yu Z. Advancements and challenges in triple-negative breast cancer: a comprehensive review of therapeutic and diagnostic strategies. Front Oncol. 2024. 10.3389/fonc.2024.1405491/full. 38863622 10.3389/fonc.2024.1405491 PMC11165151

[20] Wu Q, Li Z, Zhou X, Wei Z, Ramadan S, Xu Y, et al. Photothermal ferrotherapy-induced immunogenic cell death via iron-based ternary chalcogenide nanoparticles against triple-negative breast cancer. Small. 2024;20(20):2306766. 10.1002/smll.202306766 38095479

[21] Xu B, Sun H, Liu S, Liao L, Song X, Wu Y, et al. IFI35 limits antitumor immunity in triple-negative breast cancer via CCL2 secretion. Oncogene. 2024;43(10):693–702. 38216673 10.1038/s41388-023-02934-w PMC10907302

[22] Li Z, Qiu Y, Lu W, Jiang Y, Wang J. Immunotherapeutic interventions of triple negative breast cancer. J Transl Med. 2018;16(1):147. 10.1186/s12967-018-1514-7. 29848327 10.1186/s12967-018-1514-7 PMC5977468

[23] Bourgeois-Daigneault MC, Roy DG, Aitken AS, El Sayes N, Martin NT, Varette O, et al. Neoadjuvant oncolytic virotherapy before surgery sensitizes triple-negative breast cancer to immune checkpoint therapy. Sci Transl Med. 2018. 10.1126/scitranslmed.aao1641. 29298865 10.1126/scitranslmed.aao1641

[24] Schreier A, Zappasodi R, Serganova I, Brown KA, Demaria S, Andreopoulou E. Facts and perspectives: implications of tumor glycolysis on immunotherapy response in triple negative breast cancer. Front Oncol. 2023. 10.3389/fonc.2022.1061789/full. 36703796 10.3389/fonc.2022.1061789 PMC9872136

[25] Fermaintt CS, Peramuna T, Cai S, Takahashi-Ruiz L, Essif JN, Grant CV, et al. Yuanhuacine is a potent and selective inhibitor of the basal-like 2 subtype of triple negative breast cancer with immunogenic potential. Cancers (Basel). 2021;13(11):2834. 34200174 10.3390/cancers13112834 PMC8201195

[26] Demirsoy S, Tran H, Liu J, Li Y, Yang S, Aregawi D, et al. Targeting Tyro3, Axl, and MerTK receptor tyrosine kinases significantly sensitizes triple-negative breast cancer to CDK4/6 inhibition. Cancers (Basel). 2024;16(12):2253. 38927958 10.3390/cancers16122253 PMC11202171

[27] Ji P, Gong Y, Jin M, Wu H, Guo LW, Pei YC, et al. In vivo multidimensional CRISPR screens identify Lgals2 as an immunotherapy target in triple-negative breast cancer. Sci Adv. 2022. 10.1126/sciadv.abl8247. 35767614 10.1126/sciadv.abl8247 PMC9242595

[28] Lee J. Current treatment landscape for early triple-negative breast cancer (TNBC). J Clin Med. 2023;12(4):1524. 36836059 10.3390/jcm12041524 PMC9962369

[29] Aparicio B, Repáraz D, Ruiz M, Llopiz D, Silva L, Vercher E, et al. Identification of HLA class I-restricted immunogenic neoantigens in triple negative breast cancer. Front Immunol. 2022. 10.3389/fimmu.2022.985886/full. 36405725 10.3389/fimmu.2022.985886 PMC9666480

[30] Feng X, Zhang J, Wu L, Lin W, Liu Z, Zhou X, et al. Drug self-delivery nanocubes enhance O_2_-economized photodynamic-immunotherapy of triple-negative breast cancer by downregulating Wnt/ β -catenin signaling. Adv Healthc Mater. 2023. 10.1002/adhm.202203019. 37104840 10.1002/adhm.202203019

[31] Klempner SJ, Fabrizio D, Bane S, Reinhart M, Peoples T, Ali SM, et al. Tumor mutational burden as a predictive biomarker for response to immune checkpoint inhibitors: a review of current evidence. Oncologist. 2020;25(1):e147–59. 31578273 10.1634/theoncologist.2019-0244 PMC6964127

[32] Sha D, Jin Z, Budczies J, Kluck K, Stenzinger A, Sinicrope FA. Tumor mutational burden as a predictive biomarker in solid tumors. Cancer Discov. 2020;10(12):1808–25. 33139244 10.1158/2159-8290.CD-20-0522 PMC7710563

[33] Parikh K, Huether R, White K, Hoskinson D, Beaubier N, Dong H, et al. Tumor mutational burden from tumor-only sequencing compared with germline subtraction from paired tumor and normal specimens. JAMA Netw open. 2020;3(2):e200202. 32108894 10.1001/jamanetworkopen.2020.0202 PMC7049088

[34] Ikeda T, Oi H, Yoh K, Matsumoto S, Kato T, Nishino K, et al. Optimal next-generation sequencing (NGS) panel for estimating tumor mutation burden (TMB) and its clinical implication for non-small cell lung cancer (NSCLC). J Clin Oncol. 2021;39(15_suppl):9097–9097. 10.1200/JCO.2021.39.15_suppl.9097.

[35] O’Meara TA, Tolaney SM. Tumor mutational burden as a predictor of immunotherapy response in breast cancer. Oncotarget. 2021;12(5):394–400. 33747355 10.18632/oncotarget.27877 PMC7939529

[36] Kim JY, Kronbichler A, Eisenhut M, Hong SH, van der Vliet HJ, Kang J, et al. Tumor mutational burden and efficacy of immune checkpoint inhibitors: a systematic review and meta-analysis. Cancers (Basel). 2019. 10.3390/cancers11111798. 31731749 10.3390/cancers11111798 PMC6895916

[37] Wu HX, Wang ZX, Zhao Q, Chen DL, He MM, Yang LP, et al. Tumor mutational and indel burden: a systematic pan-cancer evaluation as prognostic biomarkers. Ann Transl Med. 2019;7(22):640–640. 31930041 10.21037/atm.2019.10.116 PMC6944566

[38] Cao D, Xu H, Xu X, Guo T, Ge W. High tumor mutation burden predicts better efficacy of immunotherapy: a pooled analysis of 103078 cancer patients. Oncoimmunology. 2019;8(9):e1629258. 10.1080/2162402X.2019.1629258. 31428527 10.1080/2162402X.2019.1629258 PMC6685508

[39] Asmann YW, Parikh K, Bergsagel PL, Dong H, Adjei AA, Borad MJ, et al. Inflation of tumor mutation burden by tumor-only sequencing in under-represented groups. NPJ Precis Oncol. 2021;5(1):22. 33742076 10.1038/s41698-021-00164-5 PMC7979755

[40] Budczies J, Allgäuer M, Litchfield K, Rempel E, Christopoulos P, Kazdal D, et al. Optimizing panel-based tumor mutational burden (TMB) measurement. Ann Oncol. 2019;30(9):1496–506. 31268125 10.1093/annonc/mdz205

[41] Ota N, Yoshimoto Y, Darwis NDM, Sato H, Ando K, Oike T, et al. High tumor mutational burden predicts worse prognosis for cervical cancer treated with radiotherapy. Jpn J Radiol. 2022;40(5):534–41. 10.1007/s11604-021-01230-5. 34860358 10.1007/s11604-021-01230-5 PMC9068645

[42] Ravaioli S, Limarzi F, Tumedei MM, Palleschi M, Maltoni R, Bravaccini S. Are we ready to use TMB in breast cancer clinical practice? Cancer Immunol Immunother. 2020;69(10):1943–5. 10.1007/s00262-020-02682-w. 32725361 10.1007/s00262-020-02682-w PMC11027700

[43] Wu Y, Xu J, Xu J, Wang Y, Wang L, Lv W, et al. The predictive value of tumor mutation burden for immune checkpoint inhibitors therapy in non-small cell lung cancer is affected by patients’ age. Biomark Res. 2020;8(1):9. 10.1186/s40364-020-00188-2. 32308981 10.1186/s40364-020-00188-2 PMC7146978

[44] Wang S, Zhang J, He Z, Wu K, Liu X. The predictive power of tumor mutational burden in lung cancer immunotherapy response is influenced by patients’ sex. Int J Cancer. 2019;145(10):2840–9. 10.1002/ijc.32327. 30972745 10.1002/ijc.32327

[45] Huang T, Chen X, Zhang H, Liang Y, Li L, Wei H, et al. Prognostic role of tumor mutational burden in cancer patients treated with immune checkpoint inhibitors: a systematic review and meta-analysis. Front Oncol. 2021. 10.3389/fonc.2021.706652/full. 34395281 10.3389/fonc.2021.706652 PMC8358612

[46] Barroso-Sousa R. Tumor mutational burden in breast cancer: current evidence, challenges, and opportunities. Cancers (Basel). 2023;15(15):3997. 37568813 10.3390/cancers15153997 PMC10417019

[47] Dixon-Douglas J, Loibl S, Denkert C, Telli M, Loi S. Integrating immunotherapy into the treatment landscape for patients with triple-negative breast cancer. Am Soc Clin Oncol Educ B. 2022;42:47–59. 10.1200/EDBK_351186. 10.1200/EDBK_351186 35649211

[48] Kwapisz D. Pembrolizumab and atezolizumab in triple-negative breast cancer. Cancer Immunol Immunother. 2021;70(3):607–17. 10.1007/s00262-020-02736-z. 33015734 10.1007/s00262-020-02736-z PMC10992894

[49] Osipov A, Lim SJ, Popovic A, Azad NS, Laheru DA, Zheng L, et al. Tumor mutational burden, toxicity, and response of immune checkpoint inhibitors targeting PD(L)1, CTLA-4, and combination: a meta-regression analysis. Clin Cancer Res. 2020;26(18):4842–51. 32586938 10.1158/1078-0432.CCR-20-0458 PMC7501151

[50] Barroso-Sousa R, Keenan TE, Pernas S, Exman P, Jain E, Garrido-Castro AC, et al. Tumor mutational burden and PTEN alterations as molecular correlates of response to PD-1/L1 blockade in metastatic triple-negative breast cancer. Clin Cancer Res. 2020;26(11):2565–72. 10.1158/1078-0432.CCR-19-3507. 32019858 10.1158/1078-0432.CCR-19-3507 PMC7269810

[51] Han Y, Wang J, Sun T, Ouyang Q, Li J, Yuan J, et al. Predictive biomarkers of response and survival following immunotherapy with a PD-L1 inhibitor benmelstobart (TQB2450) and antiangiogenic therapy with a VEGFR inhibitor anlotinib for pretreated advanced triple negative breast cancer. Signal Transduct Target Ther. 2023;8(1):429. 37973901 10.1038/s41392-023-01672-5 PMC10654734

[52] Dixon-Douglas J, Loi S. Immunotherapy in early-stage triple-negative breast cancer: where are we now and where are we headed? Curr Treat Options Oncol. 2023;24(8):1004–20. 37222922 10.1007/s11864-023-01087-y PMC10356641

[53] Tan Q, Yin S, Zhou D, Chi Y, Man X, Li H. Potential predictive and prognostic value of biomarkers related to immune checkpoint inhibitor therapy of triple-negative breast cancer. Front Oncol. 2022. 10.3389/fonc.2022.779786/full. 35646659 10.3389/fonc.2022.779786 PMC9134495

[54] Yang W, Qiu Z, Zhang J, Zhi X, Yang L, Qiu M, et al. Correlation between immune cell infiltration and PD-L1 expression and immune-related lncRNA determination in triple-negative breast cancer. Front Genet. 2022. 10.3389/fgene.2022.878658/full. 35432487 10.3389/fgene.2022.878658 PMC9008733

[55] Gao Y, Yang C, He N, Zhao G, Wang J, Yang Y. Integration of the tumor mutational burden and tumor heterogeneity identify an immunological subtype of melanoma with favorable survival. Front Oncol. 2020. 10.3389/fonc.2020.571545. 33194669 10.3389/fonc.2020.571545 PMC7661856

[56] Loupakis F, Depetris I, Biason P, Intini R, Prete AA, Leone F, et al. Prediction of benefit from checkpoint inhibitors in mismatch repair deficient metastatic colorectal cancer: role of tumor infiltrating lymphocytes. Oncologist. 2020;25(6):481–7. 31967692 10.1634/theoncologist.2019-0611 PMC7288636

[57] Yan N, Zhang H, Shen S, Guo S, Li X. Response to immune checkpoint inhibitor combination therapy in metastatic RET-mutated lung cancer from real-world retrospective data. BMC Cancer. 2024;24(1):178. 10.1186/s12885-024-11852-3. 38317126 10.1186/s12885-024-11852-3 PMC10845679

[58] Xue W, Wang Y, Xie Y, Yang C, Gong Z, Guan C, et al. miRNA-based signature associated with tumor mutational burden in colon adenocarcinoma. Front Oncol. 2021. 10.3389/fonc.2021.634841/full. 34262855 10.3389/fonc.2021.634841 PMC8274454

[59] Vokes NI, Liu D, Ricciuti B, Jimenez-Aguilar E, Rizvi H, Dietlein F, et al. Harmonization of tumor mutational burden quantification and association with response to immune checkpoint blockade in non-small-cell lung cancer. JCO Precis Oncol. 2019;3:1–12. 10.1200/PO.19.00171. 10.1200/PO.19.00171 PMC6907021 31832578

[60] Liu Z, Li M, Jiang Z, Wang X. A comprehensive immunologic portrait of triple-negative breast cancer. Transl Oncol. 2018;11(2):311–29. 29413765 10.1016/j.tranon.2018.01.011 PMC5884188

[61] Shi Y, Jin J, Ji W, Guan X. Therapeutic landscape in mutational triple negative breast cancer. Mol Cancer. 2018;17(1):99. 10.1186/s12943-018-0850-9. 30007403 10.1186/s12943-018-0850-9 PMC6046102

[62] Park JD, Kim KS, Choi SH, Jo GH, Choi JH, Park SW, et al. ELK3 modulates the antitumor efficacy of natural killer cells against triple negative breast cancer by regulating mitochondrial dynamics. J Immunother Cancer. 2022;10(7):e004825. 10.1136/jitc-2022-004825. 35858708 10.1136/jitc-2022-004825 PMC9305827

[63] Ribeiro R, Carvalho MJ, Goncalves J, Moreira JN. Immunotherapy in triple-negative breast cancer: Insights into tumor immune landscape and therapeutic opportunities. Front Mol Biosci. 2022;9:903065. 36060249 10.3389/fmolb.2022.903065 PMC9437219

[64] Loizides S, Constantinidou A. Triple negative breast cancer: immunogenicity, tumor microenvironment, and immunotherapy. Front Genet. 2023;13:1095839. 36712858 10.3389/fgene.2022.1095839 PMC9879323

[65] Gupta P, Thakur T, Chadda A, Irinike S, Khare S, Bal A. A pilot study on BRCA1/2 and PI3K mutations across subtypes of triple negative breast cancer in North Indian population. Appl Immunohistochem Mol Morphol. 2024;32(10):462–8. 39506289 10.1097/PAI.0000000000001231

[66] Cossu-Rocca P, Orrù S, Muroni MR, Sanges F, Sotgiu G, Ena S, et al. Analysis of *PIK3CA* mutations and activation pathways in triple negative breast cancer. PLoS ONE. 2015;10(11):e0141763. 10.1371/journal.pone.0141763. 26540293 10.1371/journal.pone.0141763 PMC4634768

[67] Gao C, Li H, Liu C, Xu X, Zhuang J, Zhou C, et al. Tumor mutation burden and immune invasion characteristics in triple negative breast cancer: genome high-throughput data analysis. Front Immunol. 2021;12:650491. 33968045 10.3389/fimmu.2021.650491 PMC8097167

[68] Lehmann BD, Bauer JA, Chen X, Sanders ME, Chakravarthy AB, Shyr Y, et al. Identification of human triple-negative breast cancer subtypes and preclinical models for selection of targeted therapies. J Clin Investig. 2011;121(7):2750–67. 21633166 10.1172/JCI45014 PMC3127435

[69] Masuda H, Baggerly KA, Wang Y, Zhang Y, Gonzalez-Angulo AM, Meric-Bernstam F, et al. Differential pathologic complete response rates after neoadjuvant chemotherapy among molecular subtypes of triple-negative breast cancer. J Clin Oncol. 2013;31(15_suppl):1005–1005.

[70] Lehmann BD, Jovanović B, Chen X, Estrada MV, Johnson KN, Shyr Y, et al. Refinement of triple-negative breast cancer molecular subtypes: implications for neoadjuvant chemotherapy selection; 2016. [cited 2023 Nov 30] 10.1371/journal.pone.0157368 PMC4911051 27310713

[71] Qayoom H, Mehraj U, Aisha S, Sofi S, Ahmad Mir M. Integrating immunotherapy with chemotherapy: a new approach to drug repurposing. In: Drug repurposing - molecular aspects and therapeutic applications [Internet]. IntechOpen; 2022. Available from: https://www.intechopen.com/chapters/78716.

[72] Das K, Paul S, Ghosh A, Gupta S, Mukherjee T, Shankar P, et al. Extracellular vesicles in triple-negative breast cancer: immune regulation, biomarkers, and immunotherapeutic potential. Cancers (Basel). 2023;15(19):4879. 37835573 10.3390/cancers15194879 PMC10571545

[73] Zhang J, Tian Q, Zhang M, Wang H, Wu L, Yang J. Immune-related biomarkers in triple-negative breast cancer. Breast Cancer. 2021;28(4):792–805. 10.1007/s12282-021-01247-8. 33837508 10.1007/s12282-021-01247-8 PMC8213542

[74] Kagihara JA, Andress M, Diamond JR. Nab-paclitaxel and atezolizumab for the treatment of PD-L1-positive, metastatic triple-negative breast cancer: review and future directions. Expert Rev Precis Med Drug Dev. 2020;5(2):59–65. 32190733 10.1080/23808993.2020.1730694 PMC7080186

[75] Zhang X, Ge X, Jiang T, Yang R, Li S. Research progress on immunotherapy in triple-negative breast cancer (review). Int J Oncol. 2022;61(2):95. 10.3892/ijo.2022.5385. 35762339 10.3892/ijo.2022.5385 PMC9256074

[76] Gou Q, Liu Z, Xie Y, Deng Y, Ma J, Li J, et al. Systematic evaluation of tumor microenvironment and construction of a machine learning model to predict prognosis and immunotherapy efficacy in triple-negative breast cancer based on data mining and sequencing validation. Front Pharmacol. 2022;13:995555. 36225561 10.3389/fphar.2022.995555 PMC9548553

[77] Bao R, Stapor D, Luke JJ. Molecular correlates and therapeutic targets in T cell-inflamed versus non-T cell-inflamed tumors across cancer types. Genome Med. 2020;12(1):90. 10.1186/s13073-020-00787-6. 33106165 10.1186/s13073-020-00787-6 PMC7590690

[78] Bonaventura P, Shekarian T, Alcazer V, Valladeau-Guilemond J, Valsesia-Wittmann S, Amigorena S, et al. Cold tumors: a therapeutic challenge for immunotherapy. Front Immunol. 2019. 10.3389/fimmu.2019.00168/full. 30800125 10.3389/fimmu.2019.00168 PMC6376112

[79] Zheng G, Lu Y, Yang Z, Chen H, Liang Q, Zhu Q, et al. Immune desert in MMR-deficient tumors predicts poor responsiveness of immune checkpoint inhibition. Front Immunol. 2023. 10.3389/fimmu.2023.1142862/full. 37187745 10.3389/fimmu.2023.1142862 PMC10175608

[80] Spranger S, Bao R, Gajewski TF. Melanoma-intrinsic β-catenin signalling prevents anti-tumour immunity. Nature. 2015;523(7559):231–5. 25970248 10.1038/nature14404

[81] Hammerl D, Martens JWM, Timmermans M, Smid M, Trapman-Jansen AM, Foekens R, et al. Spatial immunophenotypes predict response to anti-PD1 treatment and capture distinct paths of T cell evasion in triple negative breast cancer. Nat Commun. 2021;12(1):5668. 34580291 10.1038/s41467-021-25962-0 PMC8476574

[82] He Y, Jiang Z, Chen C, Wang X. Classification of triple-negative breast cancers based on immunogenomic profiling. J Exp Clin Cancer Res. 2018;37(1):327. 10.1186/s13046-018-1002-1. 30594216 10.1186/s13046-018-1002-1 PMC6310928

[83] Merino DM, McShane LM, Fabrizio D, Funari V, Chen SJ, White JR, et al. Establishing guidelines to harmonize tumor mutational burden (TMB): in silico assessment of variation in TMB quantification across diagnostic platforms: phase I of the Friends of Cancer Research TMB Harmonization Project. J Immunother Cancer. 2020;8(1):147. 10.1136/jitc-2019-000147 PMC7174078 32217756

[84] Wang X, Venet D, Lifrange F, Larsimont D, Rediti M, Stenbeck L, et al. Spatial transcriptomics reveals substantial heterogeneity in triple-negative breast cancer with potential clinical implications. Nat Commun. 2024;15(1):10232. 39592577 10.1038/s41467-024-54145-w PMC11599601

[85] Meléndez B, Van Campenhout C, Rorive S, Remmelink M, Salmon I, D’Haene N. Methods of measurement for tumor mutational burden in tumor tissue. Vol 7, Translational Lung Cancer Research. AME Publishing Company, pp 661–667; 2018. 10.21037/tlcr.2018.08.02 PMC6249625 30505710

[86] Fenizia F, Pasquale R, Abate RE, Lambiase M, Roma C, Bergantino F, et al. Challenges in bioinformatics approaches to tumor mutation burden analysis. Oncol Lett. 2021;22(1):1–7. 10.3892/ol.2021.12816. 10.3892/ol.2021.12816 PMC8161416 34084222

[87] Fenizia F, Wolstenholme N, Fairley JA, Rouleau E, Cheetham MH, Horan MP, et al. Tumor mutation burden testing: a survey of the International Quality Network for Pathology (IQN Path). Virchows Arch. 2021;479(6):1067–72. 10.1007/s00428-021-03093-7. 33856555 10.1007/s00428-021-03093-7 PMC8724102

[88] Vega DM, Yee LM, McShane LM, Williams PM, Chen L, Vilimas T, et al. Aligning tumor mutational burden (TMB) quantification across diagnostic platforms: phase II of the friends of cancer research TMB harmonization project. Ann Oncol. 2021;32(12):1626–36. 34606929 10.1016/j.annonc.2021.09.016

[89] Vilimas T. Measuring tumor mutational burden using whole-exome sequencing. In: Thurin M, Cesano A, Marincola FM, editors. Biomarkers for immunotherapy of cancer: methods and protocols. New York: Springer; 2020. p. 63–91. 10.1007/978-1-4939-9773-2_3. 10.1007/978-1-4939-9773-2_3 31502147

[90] Chalmers ZR, Connelly CF, Fabrizio D, Gay L, Ali SM, Ennis R, et al. Analysis of 100,000 human cancer genomes reveals the landscape of tumor mutational burden. Genome Med. 2017;9(1):34. 10.1186/s13073-017-0424-2. 28420421 10.1186/s13073-017-0424-2 PMC5395719

[91] Yao L, Fu Y, Mohiyuddin M, Lam HYK. ecTMB: a robust method to estimate and classify tumor mutational burden. Sci Rep. 2020;10(1):4983. 32188929 10.1038/s41598-020-61575-1 PMC7080796

[92] Sammons S, Elliott A, Barroso-Sousa R, Chumsri S, Tan AR, Sledge GW, et al. Concurrent predictors of an immune responsive tumor microenvironment within tumor mutational burden-high breast cancer. Front Oncol. 2023. 10.3389/fonc.2023.1235902/full. 37637072 10.3389/fonc.2023.1235902 PMC10457522

[93] Chen S, Tse K, Lu Y, Chen S, Tian Y, Tan KT, et al. Comprehensive genomic profiling and therapeutic implications for Taiwanese patients with treatment-naïve breast cancer. Cancer Med. 2024. 10.1002/cam4.7384. 38895905 10.1002/cam4.7384 PMC11187859

[94] Jin X, Yan J, Chen C, Chen Y, Huang WK. Integrated analysis of copy number variation, microsatellite instability, and tumor mutation burden identifies an 11-gene signature predicting survival in breast cancer. Front Cell Dev Biol. 2021. 10.3389/fcell.2021.721505/full. 34650974 10.3389/fcell.2021.721505 PMC8505672

[95] Zang Y, Dai C, Xu X, Cai X, Wang G, Wei J, et al. Comprehensive analysis of potential immunotherapy genomic biomarkers in 1000 Chinese patients with cancer. Cancer Med. 2019;8(10):4699–708. 10.1002/cam4.2381. 31270941 10.1002/cam4.2381 PMC6712454

[96] Chang YY, Kuo WH, Hung JH, Lee CY, Lee YH, Chang YC, et al. Deregulated microRNAs in triple-negative breast cancer revealed by deep sequencing. Mol Cancer. 2015;14(1):36. 10.1186/s12943-015-0301-9. 25888956 10.1186/s12943-015-0301-9 PMC4351690

[97] Voutsadakis IA. High mutation burden in ER-Positive/HER2-Negative/Luminal breast cancers. J Clin Med. 2022;11:1605. 35329928 10.3390/jcm11061605 PMC8953761

[98] Jardim DL, Goodman A, de Melo Gagliato D, Kurzrock R. The challenges of tumor mutational burden as an immunotherapy biomarker. Cancer Cell. 2021;39(2):154–73. 33125859 10.1016/j.ccell.2020.10.001 PMC7878292

[99] Lin J, Lin Y, Huang Z, Li X. Identification of prognostic biomarkers of cutaneous melanoma based on analysis of tumor mutation burden. Comput Math Methods Med. 2020;2020:1–14. 10.1155/2020/8836493 PMC7683164 33273963

[100] Ang C, Klempner SJ, Ali SM, Madison R, Ross JS, Severson EA, et al. Prevalence of established and emerging biomarkers of immune checkpoint inhibitor response in advanced hepatocellular carcinoma. Oncotarget. 2019;10(40):4018–25. 31258846 10.18632/oncotarget.26998 PMC6592287

[101] Xu Z, Xiang L, Wang R, Xiong Y, Zhou H, Gu H, et al. Bioinformatic analysis of immune significance of RYR2 mutation in breast cancer. Biomed Res Int. 2021;2021:1–12. 34888385 10.1155/2021/8072796 PMC8651385

[102] Craig DJ, Bailey MM, Noe OB, Williams KK, Stanbery L, Hamouda DM, et al. Subclonal landscape of cancer drives resistance to immune therapy. Cancer Treat Res Commun. 2022;30:100507. 35007928 10.1016/j.ctarc.2021.100507

[103] Zhang J, An L, Zhou X, Shi R, Wang H. Analysis of tumor mutation burden combined with immune infiltrates in endometrial cancer. Ann Transl Med. 2021;9(7):551–551. 33987249 10.21037/atm-20-6049 PMC8105813

[104] Bailey NG. Visualization of the effect of assay size on the error profile of tumor mutational burden measurement. Genes (Basel). 2022;13(3):432. 35327986 10.3390/genes13030432 PMC8949329

[105] Wang J, Zhang X, Li J, Ma X, Feng F, Liu L, et al. ADRB1 was identified as a potential biomarker for breast cancer by the co-analysis of tumor mutational burden and immune infiltration. Aging (Albany NY). 2021;13(1):351–63. 10.18632/aging.104204. 10.18632/aging.104204 PMC7835009 33234738

[106] Jiang T, Shi T, Zhang H, Hu J, Song Y, Wei J, et al. Tumor neoantigens: from basic research to clinical applications. J Hematol Oncol. 2019;12(1):93. 10.1186/s13045-019-0787-5. 31492199 10.1186/s13045-019-0787-5 PMC6731555

[107] Kina E, Laverdure JP, Durette C, Lanoix J, Courcelles M, Zhao Q, et al. Breast cancer immunopeptidomes contain numerous shared tumor antigens. J Clin Investig. 2024. 10.1172/JCI166740. 37906288 10.1172/JCI166740 PMC10760959

[108] Zhang X, Qi Y, Zhang Q, Liu W. Application of mass spectrometry-based MHC immunopeptidome profiling in neoantigen identification for tumor immunotherapy. Biomed Pharmacother. 2019;120:109542. 31629254 10.1016/j.biopha.2019.109542

[109] Ternette N, Olde Nordkamp MJM, Müller J, Anderson AP, Nicastri A, Hill AVS, et al. Immunopeptidomic profiling of HLA-A2-positive triple negative breast cancer identifies potential immunotherapy target antigens. Proteomics. 2018. 10.1002/pmic.201700465. 29786170 10.1002/pmic.201700465 PMC6032843

[110] Shao Y, Zhang T, Celiker B, Fujiwara K. Direct identification of T cell epitopes in cancer tissues. Ann Pancreat Cancer. 2023;6:3–3. 38107089 10.21037/apc-2023-1 PMC10722904

[111] Matsumoto S, Tsujikawa T, Tokita S, Mohamed Bedeir M, Matsuo K, Hata F, et al. HLA class II neoantigen presentation for CD4 + T cell surveillance in HLA class II-negative colorectal cancer. Oncoimmunology. 2024. 10.1080/2162402X.2024.2404665. 39508845 10.1080/2162402X.2024.2404665 PMC11542397

[112] Cabeza-Cabrerizo M, Cardoso A, Minutti CM, Pereira da Costa M, Reis e Sousa C. Dendritic cells revisited. Annu Rev Immunol. 2021;39(1):131–66. 10.1146/annurev-immunol-061020-053707. 33481643 10.1146/annurev-immunol-061020-053707

[113] Kloor M, Reuschenbach M, Pauligk C, Karbach J, Rafiyan MR, Al-Batran SE, et al. A frameshift peptide neoantigen-based vaccine for mismatch repair-deficient cancers: a phase I/IIa clinical trial. Clin Cancer Res. 2020;26(17):4503–10. 32540851 10.1158/1078-0432.CCR-19-3517

[114] Charneau J, Suzuki T, Shimomura M, Fujinami N, Mishima Y, Hiranuka K, et al. Development of antigen-prediction algorithm for personalized neoantigen vaccine using human leukocyte antigen transgenic mouse. Cancer Sci. 2022;113(4):1113–24. 10.1111/cas.15291. 35122353 10.1111/cas.15291 PMC8990807

[115] Oosting LT, Franke K, Martin MV, Kloosterman WP, Jamieson JA, Glenn LA, et al. Development of a personalized tumor neoantigen based vaccine formulation (FRAME-001) for use in a phase II trial for the treatment of advanced non-small cell lung cancer. Pharmaceutics. 2022;14(7):1515. 35890409 10.3390/pharmaceutics14071515 PMC9322189

[116] Sun Y, Xiong B, Shuai X, Li J, Wang C, Guo J, et al. Downregulation of HNRNPA1 induced neoantigen generation via regulating alternative splicing. Mol Med. 2024;30(1):85. 10.1186/s10020-024-00849-0. 38867190 10.1186/s10020-024-00849-0 PMC11167825

[117] Guo Z, Yuan Y, Chen C, Lin J, Ma Q, Liu G, et al. Durable complete response to neoantigen-loaded dendritic-cell vaccine following anti-PD-1 therapy in metastatic gastric cancer. Npj Precis Oncol. 2022;6(1):34. 35661819 10.1038/s41698-022-00279-3 PMC9166775

[118] Shukla SA, Howitt BE, Wu CJ, Konstantinopoulos PA. Predicted neoantigen load in non-hypermutated endometrial cancers: correlation with outcome and tumor-specific genomic alterations. Gynecol Oncol Rep. 2017;19:42–5. 28070553 10.1016/j.gore.2016.12.009 PMC5219603

[119] Zhu Y, Liu J. The role of neoantigens in cancer immunotherapy. Front Oncol. 2021. 10.3389/fonc.2021.682325/full. 34513673 10.3389/fonc.2021.682325 PMC8429900

[120] Blucher AS, Mills GB, Tsang YH. Precision oncology for breast cancer through clinical trials. Clin Exp Metastasis. 2022;39(1):71–8. 10.1007/s10585-021-10092-0. 33950412 10.1007/s10585-021-10092-0

[121] Wu SY, Wang H, Shao ZM, Jiang YZ. Triple-negative breast cancer: new treatment strategies in the era of precision medicine. Sci China Life Sci. 2021;64(3):372–88. 10.1007/s11427-020-1714-8. 32803712 10.1007/s11427-020-1714-8

[122] Natesh J, Penta D, Meeran SM (2022) Chapter 3. Epigenetics in precision medicine of breast cancer. In: García-Giménez JL (ed) Epigenetics in precision medicine. Academic Press, pp 43–67. (Translational Epigenetics; vol 30)

[123] Kenn M, Cacsire Castillo-Tong D, Singer CF, Karch R, Cibena M, Koelbl H, et al. Decision theory for precision therapy of breast cancer. Sci Rep. 2021;11(1):4233. 33608588 10.1038/s41598-021-82418-7 PMC7895957

[124] Jin J, Tao Z, Cao J, Li T, Hu X. DNA damage response inhibitors: an avenue for TNBC treatment. Biochim Biophys Acta Rev Cancer. 2021;1875(2):188521. 33556453 10.1016/j.bbcan.2021.188521

[125] Li Y, Zhan Z, Yin X, Fu S, Deng X. Targeted therapeutic strategies for triple-negative breast cancer. Front Oncol. 2021. 10.3389/fonc.2021.731535. 34778045 10.3389/fonc.2021.731535 PMC8581040

[126] Vagia E, Mahalingam D, Cristofanilli M. The landscape of targeted therapies in TNBC. Cancers (Basel). 2020;12(4):916. 32276534 10.3390/cancers12040916 PMC7226210

[127] Hossain F, Majumder S, David J, Miele L. Precision medicine and triple-negative breast cancer: current landscape and future directions. Cancers (Basel). 2021;13(15):3739. 34359640 10.3390/cancers13153739 PMC8345034

[128] Chadar R, Afsana, Kesharwani P. Nanotechnology-based siRNA delivery strategies for treatment of triple negative breast cancer. Int J Pharm. 2021;605:120835. 34197908 10.1016/j.ijpharm.2021.120835

[129] Garufi G, Palazzo A, Paris I, Orlandi A, Cassano A, Tortora G, et al. Neoadjuvant therapy for triple-negative breast cancer: potential predictive biomarkers of activity and efficacy of platinum chemotherapy, PARP- and immune-checkpoint-inhibitors. Expert Opin Pharmacother. 2020;21(6):687–99. 32052646 10.1080/14656566.2020.1724957

[130] Subhan MA, Parveen F, Shah H, Yalamarty SSK, Ataide JA, Torchilin VP. Recent advances with precision medicine treatment for breast cancer including triple-negative sub-type. Cancers (Basel). 2023;15(8):2204. 37190133 10.3390/cancers15082204 PMC10137302

[131] Khan S, Jandrajupalli SB, Bushara NZA, Raja RDP, Mirza S, Sharma K, et al. Targeting refractory triple-negative breast cancer with Sacituzumab Govitecan: a new era in precision medicine. Cells. 2024;13(24):2126. 39768216 10.3390/cells13242126 PMC11674573

[132] Ryu WJ, Sohn JH. Molecular targets and promising therapeutics of triple-negative breast cancer. Pharmaceuticals. 2021;14(10):1008. 34681231 10.3390/ph14101008 PMC8540846

[133] Park JH, Ahn JH, Kim SB. How shall we treat early triple-negative breast cancer (TNBC): from the current standard to upcoming immuno-molecular strategies. ESMO Open. 2018;3:e000357. 29765774 10.1136/esmoopen-2018-000357 PMC5950702

[134] Cai SL, Liu JJ, Liu YX, Yu SH, Liu X, Lin XQ, et al. Characteristics of recurrence, predictors for relapse and prognosis of rapid relapse triple-negative breast cancer. Front Oncol. 2023;16(13):1119611. 10.3389/fonc.2023.1119611 PMC9978400 36874102

[135] Garrido-Castro AC, Saura C, Barroso-Sousa R, Guo H, Ciruelos E, Bermejo B, et al. Phase 2 study of buparlisib (BKM120), a pan-class I PI3K inhibitor, in patients with metastatic triple-negative breast cancer. Breast Cancer Res. 2020;22(1):120. 33138866 10.1186/s13058-020-01354-y PMC7607628

[136] Yan C, Yang J, Saleh N, Chen SC, Ayers GD, Abramson VG, et al. Inhibition of the PI3K/mTOR pathway in breast cancer to enhance response to immune checkpoint inhibitors in breast cancer. Int J Mol Sci. 2021;22(10):5207. 34069042 10.3390/ijms22105207 PMC8156389

[137] Doha ZO, Wang X, Calistri NL, Eng J, Daniel CJ, Ternes L, et al. MYC deregulation and PTEN loss model tumor and stromal heterogeneity of aggressive triple-negative breast cancer. Nat Commun. 2023;14(1):5665. 37704631 10.1038/s41467-023-40841-6 PMC10499828

[138] Chen H, Ding Q, Khazai L, Zhao L, Damodaran S, Litton JK, et al. PTEN in triple-negative breast carcinoma: protein expression and genomic alteration in pretreatment and posttreatment specimens. Ther Adv Med Oncol. 2023. 10.1177/17588359231189422. 37547448 10.1177/17588359231189422 PMC10399250

[139] Zhang H, Jiang R, Zhu J, Sun K, Huang Y, Zhou H, et al. PI3K/AKT/mTOR signaling pathway: an important driver and therapeutic target in triple-negative breast cancer. Breast Cancer. 2024;31(4):539–51. 10.1007/s12282-024-01567-5. 38630392 10.1007/s12282-024-01567-5 PMC11194209

[140] Zhang W, Wang X, Gao S, Chen C, Xu X, Sun Q, et al. Tumor-associated macrophages correlate with phenomenon of epithelial-mesenchymal transition and contribute to poor prognosis in triple-negative breast cancer patients. J Surg Res. 2018;222:93–101. 29273380 10.1016/j.jss.2017.09.035

[141] Fu LQ, Du WL, Cai MH, Yao JY, Zhao YY, Mou XZ. The roles of tumor-associated macrophages in tumor angiogenesis and metastasis. Cell Immunol. 2020;353:104119. 32446032 10.1016/j.cellimm.2020.104119

[142] Giatromanolaki A, Gkegka AG, Pouliliou S, Biziota E, Kakolyris S, Koukourakis M. Hypoxia and anaerobic metabolism relate with immunologically cold breast cancer and poor prognosis. Breast Cancer Res Treat. 2022;194(1):13–23. 10.1007/s10549-022-06609-0. 35482128 10.1007/s10549-022-06609-0

[143] Yang L, Chu Z, Liu M, Zou Q, Li J, Liu Q, et al. Amino acid metabolism in immune cells: essential regulators of the effector functions, and promising opportunities to enhance cancer immunotherapy. J Hematol Oncol. 2023;16(1):59. 10.1186/s13045-023-01453-1. 37277776 10.1186/s13045-023-01453-1 PMC10240810

[144] Hasmim M, Xiao M, Van Moer K, Kumar A, Oniga A, Mittelbronn M, et al. SNAI1-dependent upregulation of CD73 increases extracellular adenosine release to mediate immune suppression in TNBC. Front Immunol. 2022. 10.3389/fimmu.2022.982821/full. 36159844 10.3389/fimmu.2022.982821 PMC9501677

[145] Xie X, Zhang J, Shi Z, Liu W, Hu X, Qie C, et al. The expression pattern and clinical significance of the immune checkpoint regulator VISTA in human breast cancer. Front Immunol. 2020. 10.3389/fimmu.2020.563044. 33250890 10.3389/fimmu.2020.563044 PMC7673447

[146] Racioppi L, Nelson ER, Huang W, Mukherjee D, Lawrence SA, Lento W, et al. CaMKK2 in myeloid cells is a key regulator of the immune-suppressive microenvironment in breast cancer. Nat Commun. 2019;10(1):2450. 31164648 10.1038/s41467-019-10424-5 PMC6547743

[147] Naorem LD, Muthaiyan M, Venkatesan A. Identification of dysregulated miRNAs in triple negative breast cancer: a meta-analysis approach. J Cell Physiol. 2019;234(7):11768–79. 10.1002/jcp.27839. 30488443 10.1002/jcp.27839

[148] Xu L, Che X, Wu Y, Song N, Shi S, Wang S, et al. SIRT5 as a biomarker for response to anthracycline-taxane-based neoadjuvant chemotherapy in triple-negative breast cancer. Oncol Rep. 2018. 10.3892/or.2018.6319. 29565454 10.3892/or.2018.6319

[149] Anand K, Patel T, Niravath P, Rodriguez A, Darcourt J, Belcheva A, et al. Targeting mTOR and DNA repair pathways in residual triple negative breast cancer post neoadjuvant chemotherapy. Sci Rep. 2021;11(1):82. 33420229 10.1038/s41598-020-80081-y PMC7794349

[150] Murphy C, Muscat A, Ashley D, Mukaro V, West L, Liao Y, et al. Tailored NEOadjuvant epirubicin, cyclophosphamide and Nanoparticle Albumin-Bound paclitaxel for breast cancer: the phase II NEONAB trial—clinical outcomes and molecular determinants of response. PLoS ONE. 2019;14(2):e0210891. 10.1371/journal.pone.0210891. 30763338 10.1371/journal.pone.0210891 PMC6375556

[151] Moamin MR, Allen R, Woods SL, Brown JE, Nunns H, Juncker-Jensen A, et al. Changes in the immune landscape of TNBC after neoadjuvant chemotherapy: correlation with relapse. Front Immunol. 2023. 10.3389/fimmu.2023.1291643/full. 38090569 10.3389/fimmu.2023.1291643 PMC10715438

[152] Stavrou M, Constantinidou A. Tumor associated macrophages in breast cancer progression: implications and clinical relevance. Front Immunol. 2024. 10.3389/fimmu.2024.1441820/full. 39044824 10.3389/fimmu.2024.1441820 PMC11263030

[153] Belgiovine C, Frapolli R, Liguori M, Digifico E, Colombo FS, Meroni M, et al. Inhibition of tumor-associated macrophages by trabectedin improves the antitumor adaptive immunity in response to anti-PD-1 therapy. Eur J Immunol. 2021;51(11):2677–86. 10.1002/eji.202149379. 34570376 10.1002/eji.202149379 PMC9293411

[154] Krebber MM, van Dijk CGM, Vernooij RWM, Brandt MM, Emter CA, Rau CD, et al. Matrix metalloproteinases and tissue inhibitors of metalloproteinases in extracellular matrix remodeling during left ventricular diastolic dysfunction and heart failure with preserved ejection fraction: a systematic review and meta-analysis. Int J Mol Sci. 2020;21(18):6742. 32937927 10.3390/ijms21186742 PMC7555240

[155] Asleh K, Brauer HA, Sullivan A, Lauttia S, Lindman H, Nielsen TO, et al. Predictive biomarkers for adjuvant capecitabine benefit in early-stage triple-negative breast cancer in the FinXX clinical trial. Clin Cancer Res. 2020;26(11):2603–14. 32005747 10.1158/1078-0432.CCR-19-1945

[156] Ho J, Chan H, Wong SH, Wang MHT, Yu J, Xiao Z, et al. The involvement of regulatory non-coding RNAs in sepsis: a systematic review. Crit Care. 2016;20(1):383. 10.1186/s13054-016-1555-3. 27890015 10.1186/s13054-016-1555-3 PMC5125038

[157] Keenan TE, Guerriero JL, Barroso-Sousa R, Li T, O’Meara T, Giobbie-Hurder A, et al. Molecular correlates of response to eribulin and pembrolizumab in hormone receptor-positive metastatic breast cancer. Nat Commun. 2021;12(1):5563. 34548479 10.1038/s41467-021-25769-z PMC8455578

[158] Wood MA, Weeder BR, David JK, Nellore A, Thompson RF. Burden of tumor mutations, neoepitopes, and other variants are weak predictors of cancer immunotherapy response and overall survival. Genome Med. 2020;12(1):33. 32228719 10.1186/s13073-020-00729-2 PMC7106909

[159] Zou Y, Hu X, Zheng S, Yang A, Li X, Tang H, et al. Discordance of immunotherapy response predictive biomarkers between primary lesions and paired metastases in tumours: a systematic review and meta-analysis. EBioMedicine. 2021;63:103137. 33310681 10.1016/j.ebiom.2020.103137 PMC7736926

[160] Chen Z, Wang X, Li X, Zhou Y, Chen K. Deep exploration of PARP inhibitors in breast cancer: monotherapy and combination therapy. J Int Med Res. 2021;49(2):030006052199101. 10.1177/0300060521991019 PMC8164563 33541181

[161] Chen H, Wu J, Zhang Z, Tang Y, Li X, Liu S, et al. Association between BRCA status and triple-negative breast cancer: a meta-analysis. Front Pharmacol. 2018. 10.3389/fphar.2018.00909/full. 30186165 10.3389/fphar.2018.00909 PMC6111442

[162] Chia S, Bedard PL, Hilton J, Amir E, Gelmon K, Goodwin R, et al. A phase Ib trial of durvalumab in combination with trastuzumab in HER2-positive metastatic breast cancer (CCTG IND.229). Oncologist. 2019;24(11):1439–45. 31420468 10.1634/theoncologist.2019-0321 PMC6853090

[163] Bu J, Nair A, Iida M, Jeong JW, Poellmann MJ, Mudd K, et al. An avidity-based PD-L1 antagonist using nanoparticle-antibody conjugates for enhanced immunotherapy. Nano Lett. 2020;20(7):4901–9. 32510959 10.1021/acs.nanolett.0c00953 PMC7737517

[164] Stanton SE, Disis ML. Clinical significance of tumor-infiltrating lymphocytes in breast cancer. J Immunother Cancer. 2016;4(1):59. 27777769 10.1186/s40425-016-0165-6 PMC5067916

[165] Alkaabi D, Arafat K, Sulaiman S, Al-Azawi AM, Attoub S. PD-1 independent role of PD-L1 in triple-negative breast cancer progression. Int J Mol Sci. 2023;24(7):6420. 37047395 10.3390/ijms24076420 PMC10094894

[166] Sidders B, Zhang P, Goodwin K, O’Connor G, Russell DL, Borodovsky A, et al. Adenosine signaling is prognostic for cancer outcome and has predictive utility for immunotherapeutic response. Clin Cancer Res. 2020;26(9):2176–87. 31953314 10.1158/1078-0432.CCR-19-2183

[167] Abdou Y, Attwood K, Cheng TYD, Yao S, Bandera EV, Zirpoli GR, et al. Racial differences in CD8+ T cell infiltration in breast tumors from Black and White women. Breast Cancer Res. 2020;22(1):62. 32517730 10.1186/s13058-020-01297-4 PMC7285742

[168] Ngwa VM, Edwards DN, Philip M, Chen J. Microenvironmental metabolism regulates antitumor immunity. Cancer Res. 2019;79(16):4003–8. 31362930 10.1158/0008-5472.CAN-19-0617 PMC6697577

[169] Going CC, Tailor D, Kumar V, Birk AM, Pandrala M, Rice MA, et al. Quantitative proteomic profiling reveals key pathways in the anticancer action of methoxychalcone derivatives in triple negative breast cancer. J Proteome Res. 2018;17(10):3574–85. 30200768 10.1021/acs.jproteome.8b00636 PMC8078626

[170] Guo Z, Zhu Z, Lin X, Wang S, Wen Y, Wang L, et al. Tumor microenvironment and immunotherapy for triple-negative breast cancer. Biomark Res. 2024;12(1):166. 10.1186/s40364-024-00714-6. 39741315 10.1186/s40364-024-00714-6 PMC11689763

[171] Zhan L, Zhang B, Tan Y, Yang C, Huang C, Wu Q, et al. Quantitative assessment of the relationship between RASSF1A gene promoter methylation and bladder cancer (PRISMA). Medicine (Baltimore). 2017;96(7):e6097. 28207521 10.1097/MD.0000000000006097 PMC5319510

[172] Zhang L, Long X. Association of *BRCA1* promoter methylation with sporadic breast cancers: evidence from 40 studies. Sci Rep. 2015;5(1):17869. 26643130 10.1038/srep17869 PMC4672329

[173] Nemet J, Vidan N, Sopta M. A meta-analysis reveals complex regulatory properties at Taf14-repressed genes. BMC Genomics. 2017;18(1):175. 28209126 10.1186/s12864-017-3544-6 PMC5312515

[174] Li S, Shen L, Chen K. Association between H3K4 methylation and cancer prognosis: a meta-analysis. Thorac Cancer. 2018;9(7):794–9. 29737623 10.1111/1759-7714.12647 PMC6026618

[175] Deng Q, Luo Y, Chang C, Wu H, Ding Y, Xiao R. The emerging epigenetic role of CD8+T cells in autoimmune diseases: a systematic review. Front Immunol. 2019;10:856. 31057561 10.3389/fimmu.2019.00856 PMC6482221

[176] Ibrahim ML, Zheng H, Barlow ML, Latif Y, Chen Z, Yu X, et al. Histone deacetylase inhibitors directly modulate T cell gene expression and signaling and promote development of effector-exhausted T cells in murine tumors. J Immunol. 2024;212(4):737–47. 38169329 10.4049/jimmunol.2300475 PMC10872871

[177] Terranova-Barberio M, Thomas S, Ali N, Pawlowska N, Park J, Krings G, et al. HDAC inhibition potentiates immunotherapy in triple negative breast cancer. Oncotarget. 2017;8(69):114156–72. 10.18632/oncotarget.23169. 29371976 10.18632/oncotarget.23169 PMC5768393

[178] Qin Y, Vasilatos SN, Chen L, Wu H, Cao Z, Fu Y, et al. Inhibition of histone lysine-specific demethylase 1 elicits breast tumor immunity and enhances antitumor efficacy of immune checkpoint blockade. Oncogene. 2019;38(3):390–405. 30111819 10.1038/s41388-018-0451-5 PMC6336685

[179] Gao G, Wang Z, Qu X, Zhang Z. Prognostic value of tumor-infiltrating lymphocytes in patients with triple-negative breast cancer: a systematic review and meta-analysis. BMC Cancer. 2020;20(1):179. 32131780 10.1186/s12885-020-6668-z PMC7057662

[180] Zhang H, Qin G, Yu H, Han X, Zhu S. Comprehensive genomic and immunophenotypic analysis of CD4 T cell infiltrating human triple-negative breast cancer. Cancer Immunol Immunother. 2021;70(6):1649–65. 10.1007/s00262-020-02807-1. 33301062 10.1007/s00262-020-02807-1 PMC8139937

[181] Criscitiello C, Bayar MA, Curigliano G, Symmans FW, Desmedt C, Bonnefoi H, et al. A gene signature to predict high tumor-infiltrating lymphocytes after neoadjuvant chemotherapy and outcome in patients with triple-negative breast cancer. Ann Oncol. 2018;29(1):162–9. 29077781 10.1093/annonc/mdx691

[182] Grimaldi AM, Salvatore M, Incoronato M. MiRNA-based therapeutics in breast cancer: a systematic review. Front Oncol. 2021. 10.3389/fonc.2021.668464. 34026646 10.3389/fonc.2021.668464 PMC8131824

[183] Lü L, Mao X, Shi P, He B, Xu K, Zhang S, et al. Micrornas in the prognosis of triple-negative breast cancer. Medicine (Baltimore). 2017;96(22):e7085. 28562579 10.1097/MD.0000000000007085 PMC5459744

[184] Rasmussen L, Knorr S, Antoniussen CS, Bruun JM, Ovesen PG, Fuglsang J, et al. The impact of lifestyle, diet and physical activity on epigenetic changes in the offspring—a systematic review. Nutrients. 2021;13(8):2821. 34444981 10.3390/nu13082821 PMC8398155

[185] Fabiani R, Vella N, Rosignoli P. Epigenetic modifications induced by olive oil and its phenolic compounds: a systematic review. Molecules. 2021;26(2):273. 33430487 10.3390/molecules26020273 PMC7826507

[186] Bannister S, Messina NL, Novakovic B, Curtis N. The emerging role of epigenetics in the immune response to vaccination and infection: a systematic review. Epigenetics. 2020;15(6–7):555–93. 31914857 10.1080/15592294.2020.1712814 PMC7574386

[187] Abdulrashid K, Alhussaini N, Ahmed W, Thalib L. Prevalence of BRCA mutations among hereditary breast and/or ovarian cancer patients in Arab countries: systematic review and meta-analysis. BMC Cancer. 2019. 10.1186/s12885-019-5463-1. 30898109 10.1186/s12885-019-5463-1 PMC6429759

[188] Ouyang T, Cao Y, Kan X, Chen L, Ren Y, Sun T, et al. Treatment-related serious adverse events of immune checkpoint inhibitors in clinical trials: a systematic review. Front Oncol. 2021. 10.3389/fonc.2021.621639. 34046338 10.3389/fonc.2021.621639 PMC8144509

[189] D’Andrea E, Marzuillo C, Vito C, Marco M, Pitini E. Which BRCA genetic testing programs are ready for implementation in health care? A systematic review of economic evaluations. Genet Med. 2016;18(12):1171–80. 10.1038/gim.2016.29. 27906166 10.1038/gim.2016.29 PMC5159446

[190] Ding Y, Ding K, Yang H, He X, Mo W, Ding X. Does dose-dense neoadjuvant chemotherapy have clinically significant prognostic value in breast cancer? A meta-analysis of 3724 patients. PLoS ONE. 2020;15(5):e0234058. 10.1371/journal.pone.0234058. 32470093 10.1371/journal.pone.0234058 PMC7259732

[191] Imani S, Zhang X, Hosseinifard H, Fu S, Fu J. The diagnostic role of microrna-34a in breast cancer: a systematic review and meta-analysis. Oncotarget. 2017;8(14):23177–87. 28423566 10.18632/oncotarget.15520 PMC5410295

[192] Zhu Q, Balasubramanian A, Asirvatham JR, Piyarathna B, Kaur J, Mohamed N, et al. Integrative spatial omics reveals distinct tumor-promoting multicellular niches and immunosuppressive mechanisms in African American and European American patients with TNBC. Nat Commun. 2024. 10.1101/2024.03.17.585428. 40675986 10.1038/s41467-025-61034-3 PMC12271405

[193] Liang X, Chen X, Li H, Li Y. Immune checkpoint inhibitors in first-line therapies of metastatic or early triple-negative breast cancer: a systematic review and network meta-analysis. Front Endocrinol (Lausanne). 2023. 10.3389/fendo.2023.1137464/full. 37229447 10.3389/fendo.2023.1137464 PMC10204114

[194] Mezni E, Behi K, Gonçalves A. Immunotherapy and breast cancer: an overview. Curr Opin Oncol. 2022;34(5):587–94. 10.1097/CCO.0000000000000878. 35838204 10.1097/CCO.0000000000000878

[195] Ralser DJ, Klümper N, Gevensleben H, Zarbl R, Kaiser C, Landsberg J, et al. Molecular and immune correlates of PDCD1 (PD-1), PD-L1 (CD274), and PD-L2 (PDCD1LG2) DNA methylation in triple negative breast cancer. J Immunother. 2021;44(8):319–24. 10.1097/CJI.0000000000000384. 34347720 10.1097/CJI.0000000000000384

[196] Ensenyat-Mendez M, Orozco JIJ, Llinàs-Arias P, Íñiguez-Muñoz S, Baker JL, Salomon MP, et al. Construction and validation of a gene expression classifier to predict immunotherapy response in primary triple-negative breast cancer. Commun Med. 2023;3(1):93. 37430006 10.1038/s43856-023-00311-y PMC10333210

[197] Cho B, Han Y, Lian M, Colditz GA, Weber JD, Ma C, et al. Evaluation of racial/ethnic differences in treatment and mortality among women with triple-negative breast cancer. JAMA Oncol. 2021;7(7):1016. 33983438 10.1001/jamaoncol.2021.1254 PMC8120441

[198] Ademuyiwa FO, Tao Y, Luo J, Weilbaecher K, Ma CX. Differences in the mutational landscape of triple-negative breast cancer in African Americans and Caucasians. Breast Cancer Res Treat. 2017;161(3):491–9. 10.1007/s10549-016-4062-y. 27915434 10.1007/s10549-016-4062-y PMC5243212

[199] Dietze EC, Sistrunk C, Miranda-Carboni G, O’Regan R, Seewaldt VL. Triple-negative breast cancer in African-American women: disparities versus biology. Nat Rev Cancer. 2015;15(4):248–54. 25673085 10.1038/nrc3896 PMC5470637

[200] Ogony JW, Radisky DC, Ruddy KJ, Goodison S, Wickland DP, Egan KM, et al. Immune responses and risk of triple-negative breast cancer: implications for higher rates among African American women. Cancer Prev Res. 2020;13(11):901–10. 10.1158/1940-6207.CAPR-19-0562 PMC9576802 32753376

[201] Nassar AH, Adib E, Abou Alaiwi S, El Zarif T, Groha S, Akl EW, et al. Ancestry-driven recalibration of tumor mutational burden and disparate clinical outcomes in response to immune checkpoint inhibitors. Cancer Cell. 2022;40(10):1161-1172.e5. 36179682 10.1016/j.ccell.2022.08.022 PMC9559771

[202] O’Connell J, Yun T, Moreno M, Li H, Litterman N, Kolesnikov A, et al. A population-specific reference panel for improved genotype imputation in African Americans. Commun Biol. 2021;4(1):1269. 34741098 10.1038/s42003-021-02777-9 PMC8571350

[203] Cui M, Gao X, Gu X, Guo W, Li X, Ma M, et al. BRCA2 mutations should be screened early and routinely as markers of poor prognosis: evidence from 8988 patients with prostate cancer. Oncotarget. 2017;8(25):40222–32. 10.18632/oncotarget.16712. 28410213 10.18632/oncotarget.16712 PMC5522317

[204] Negrao VM, Skoulidis F, Montesion M, Schulze K, Bara I, Shen V, et al. Oncogene-specific differences in tumor mutational burden, PD-L1 expression, and outcomes from immunotherapy in non-small cell lung cancer. J Immunother Cancer. 2021;9(8):e002891. 10.1136/jitc-2021-002891. 34376553 10.1136/jitc-2021-002891 PMC8356172

[205] Möller K, Knöll M, Bady E, Schmerder MJ, Rico SD, Kluth M, et al. PD-L1 expression and CD8 positive lymphocytes in human neoplasms: a tissue microarray study on 11,838 tumor samples. Cancer Biomark. 2023;36(2):177–91. 10.3233/CBM-220030. 36683495 10.3233/CBM-220030 PMC9986704

[206] Dunning A, Michailidou K, Kuchenbaecker K, Thompson D, French J, Beesley J, et al. Breast cancer risk variants at 6q25 display different phenotype associations and regulate ESR1, RMND1, and CCDC170. Nat Genet. 2016;48(4):374–86. 10.1038/ng.3521. 26928228 10.1038/ng.3521 PMC4938803

[207] Du Q, Che J, Jiang X, Li L, Luo X, Li Q. PD-L1 acts as a promising immune marker to predict the response to neoadjuvant chemotherapy in breast cancer patients. Clin Breast Cancer. 2020;20(1):e99–111. 10.1016/j.clbc.2019.06.014. 31521537 10.1016/j.clbc.2019.06.014

[208] Zhao D, Yu X, Huang H, Zou S, Zhu P, Lin Y, et al. Association of the SNPs in CCL2 and CXCL12 genes with the susceptibility to breast cancer: a case–control study in China. Front Oncol. 2024. 10.3389/fonc.2024.1475979/full. 39703847 10.3389/fonc.2024.1475979 PMC11655334

[209] Lehmann B, Ding Y, Viox D, Jiang M, Zheng Y, Liao W, et al. Evaluation of public cancer datasets and signatures identifies TP53 mutant signatures with robust prognostic and predictive value. BMC Cancer. 2015. 10.1186/s12885-015-1102-7. 25886164 10.1186/s12885-015-1102-7 PMC4404582

[210] Zhong Q, Peng H, Zhao X, Zhang L, Hwang W. Effects of BRCA1- and BRCA2-related mutations on ovarian and breast cancer survival: a meta-analysis. Clin Cancer Res. 2015;21(1):211–20. 25348513 10.1158/1078-0432.CCR-14-1816 PMC4286460

[211] He X, Zou D. The association of young age with local recurrence in women with early-stage breast cancer after breast-conserving therapy: a meta-analysis. Sci Rep. 2017. 10.1038/s41598-017-10729-9. 28894168 10.1038/s41598-017-10729-9 PMC5593910

[212] Wang S, Du H, Li G. Significant prognostic value of circulating tumor cells in esophageal cancer patients: a meta-analysis. Oncotarget. 2017;8(9):15815–26. 28178659 10.18632/oncotarget.15012 PMC5362525

[213] Brawley OW, Luhn P, Reese-White D, Ogbu UC, Madhavan S, Wilson G, et al. Disparities in tumor mutational burden, immunotherapy use, and outcomes based on genomic ancestry in non-small-cell lung cancer. JCO Glob Oncol. 2021;7:1537–46. 34752134 10.1200/GO.21.00309 PMC8577674

[214] Kwiatkowski K, Coe K, Bailar JC, Swanson GM. Inclusion of minorities and women in cancer clinical trials, a decade later: Have we improved? Cancer. 2013;119(16):2956–63. 23674318 10.1002/cncr.28168

[215] Fancello L, Gandini S, Pelicci P, Mazzarella L. Tumor mutational burden quantification from targeted gene panels: major advancements and challenges. J Immunother Cancer. 2019. 10.1186/s40425-019-0647-4. 31307554 10.1186/s40425-019-0647-4 PMC6631597

[216] Herrera-Pariente C, Capó-García R, Díaz-Gay M, Carballal S, Muñoz J, Llach J, et al. Identification of new genes involved in germline predisposition to early-onset gastric cancer. Int J Mol Sci. 2021;22(3):1310. 33525650 10.3390/ijms22031310 PMC7866206

[217] Budczies J, Kazdal D, Allgäuer M, Christopoulos P, Rempel E, Pfarr N, et al. Quantifying potential confounders of panel-based tumor mutational burden (TMB) measurement. Lung Cancer. 2020;142:114–9. 32143116 10.1016/j.lungcan.2020.01.019

[218] Ranade K, Higgs BW, Raja RG, Brohawn PZ, Si H, KUZIORA MA. Blood-based tumor mutation burden predicts overall survival in nsclc [Internet]. 2023. Available from: https://patents.google.com/patent/US20230145764A1/en.

[219] Tom W, Chaudhary R, Mittal V, Cyanam D, Casuga I, Wong-Ho E, et al. Abstract 1701: improvement of tumor mutation burden measurement by removal of deaminated bases in FFPE DNA. Cancer Res. 2019;79(13_Supplement):1701–1701.

[220] Hong TH, Cha H, Shim JH, Lee B, Chung J, Lee C, et al. Clinical advantage of targeted sequencing for unbiased tumor mutational burden estimation in samples with low tumor purity. J Immunother Cancer. 2020;8(2):e001199. 10.1136/jitc-2020-001199. 33077514 10.1136/jitc-2020-001199 PMC7574938

[221] Zhang Y, Wang D, Zhao Z, Peng R, Han Y, Li J, et al. Enhancing the quality of panel-based tumor mutation burden assessment: a comprehensive study of real-world and in-silico outcomes. NPJ Precis Oncol. 2024;8(1):18. 38263314 10.1038/s41698-024-00504-1 PMC10805867

[222] Lam VK, Zhang J. Blood-based tumor mutation burden: continued progress toward personalizing immunotherapy in non-small cell lung cancer. J Thorac Dis. 2019;11(6):2208–11. 31372254 10.21037/jtd.2019.05.68 PMC6626794

[223] Raiber-Moreau E, Portella G, Butler MG, Clement O, Konigshofer Y, Hadfield J. Development and validation of blood tumor mutational burden reference standards. Genes Chromosomes Cancer. 2023;62(3):121–30. 10.1002/gcc.23100. 36326821 10.1002/gcc.23100 PMC10107199

[224] He J, Kalinava N, Doshi P, Pavlick DC, Albacker LA, Ebot EM, et al. Evaluation of tissue- and plasma-derived tumor mutational burden (TMB) and genomic alterations of interest in CheckMate 848, a study of nivolumab combined with ipilimumab and nivolumab alone in patients with advanced or metastatic solid tumors with high T. J Immunother Cancer. 2023;11(11):e007339. 10.1136/jitc-2023-007339. 38035725 10.1136/jitc-2023-007339 PMC10689409

[225] Stenzinger A, Endris V, Budczies J, Merkelbach-Bruse S, Kazdal D, Dietmaier W, et al. Harmonization and standardization of panel-based tumor mutational burden measurement: real-world results and recommendations of the quality in pathology study. J Thorac Oncol. 2020;15(7):1177–89. 32119917 10.1016/j.jtho.2020.01.023

[226] Martinez-Pacheco S, O’Driscoll L. Pre-clinical in vitro models used in cancer research: results of a worldwide survey. Cancers (Basel). 2021;13(23):6033. 34885142 10.3390/cancers13236033 PMC8656628

[227] Mu P, Zhou S, Lv T, Fan X, Shen L, Wan J, et al. Newly developed 3d in vitro models to study tumor–immune interaction. J Exp Clin Cancer Res. 2023;42(1):81. 37016422 10.1186/s13046-023-02653-w PMC10074642

[228] Chiew G, Wei N, Sultania S, Lim S, Luo K. Bioengineered three-dimensional co-culture of cancer cells and endothelial cells: a model system for dual analysis of tumor growth and angiogenesis. Biotechnol Bioeng. 2017;114(8):1865–77. 28369747 10.1002/bit.26297

[229] Alonso-Nocelo M, Abuín C, López-López R, Fuente M. Development and characterization of a three-dimensional co-culture model of tumor t cell infiltration. Biofabrication. 2016;8(2):25002. 10.1088/1758-5090/8/2/025002 27078888

[230] Xu H. Biomarkers and experimental models for cancer immunology investigation. MedComm. 2023;4(6):e437. 38045830 10.1002/mco2.437 PMC10693314

[231] Lin Y, Nasir A, Camacho S, Berry D, Schmidt M, Pearson G, et al. Monitoring cancer cell invasion and t-cell cytotoxicity in 3d culture. J Vis Exp. 2020;160:10–3791. 10.3791/61392 PMC8441943 32658183

[232] Hossain F, Ucar DA, Monticone G, Ran Y, Majumder S, Larter K, et al. Sulindac sulfide as a non-immune suppressive γ-secretase modulator to target triple-negative breast cancer. Front Immunol. 2023. 10.3389/fimmu.2023.1244159/full. 37901240 10.3389/fimmu.2023.1244159 PMC10612326

[233] Liu H, Xia B, Jin M, Lou G. Organoid of ovarian cancer: genomic analysis and drug screening. Clin Transl Oncol. 2020;22(8):1240–51. 31939100 10.1007/s12094-019-02276-8 PMC7316695

[234] Zhu L. Advancements and application prospects of three-dimensional models for primary liver cancer: a comprehensive review. Front Bioeng Biotechnol. 2023;11:1343177. 38188493 10.3389/fbioe.2023.1343177 PMC10771299

[235] Stenzinger A, Allen JD, Maas J, Stewart MD, Merino DM, Wempe MM, et al. Tumor mutational burden standardization initiatives: recommendations for consistent tumor mutational burden assessment in clinical samples to guide immunotherapy treatment decisions. Genes Chromosomes Cancer. 2019;58(8):578–88. 10.1002/gcc.22733. 30664300 10.1002/gcc.22733 PMC6618007

[236] Fancello L, Gandini S, Pelicci PG, Mazzarella L. Tumor mutational burden quantification from targeted gene panels: major advancements and challenges. J Immunother Cancer. 2019;7(1):183. 10.1186/s40425-019-0647-4. 31307554 10.1186/s40425-019-0647-4 PMC6631597

[237] McNamara MG, Jacobs T, Lamarca A, Hubner RA, Valle JW, Amir E. Impact of high tumor mutational burden in solid tumors and challenges for biomarker application. Cancer Treat Rev. 2020;89:102084. 32738738 10.1016/j.ctrv.2020.102084

[238] Sung MT, Wang YH, Li CF. Open the technical black box of tumor mutational burden (TMB): factors affecting harmonization and standardization of panel-based TMB. Int J Mol Sci. 2022;23(9):5097. 35563486 10.3390/ijms23095097 PMC9103036

[239] Heeke S, Benzaquen J, Hofman V, Long-Mira E, Lespinet V, Bordone O, et al. Comparison of three sequencing panels used for the assessment of tumor mutational burden in NSCLC reveals low comparability. J Thorac Oncol. 2020;15(9):1535–40. 32450274 10.1016/j.jtho.2020.05.013

[240] Nosková H, Kýr M, Pál K, Merta T, Múdrý P, Polaskova K, et al. Assessment of tumor mutational burden in pediatric tumors by real-life whole-exome sequencing and in silico simulation of targeted gene panels: how the choice of method could affect the clinical decision? Cancers (Basel). 2020;12(1):230. 31963488 10.3390/cancers12010230 PMC7016876

[241] Huang R, Carbone D, Li G, Schrock A, Graf R, Zhang L, et al. Durable responders in advanced nsclc with elevated tmb and treated with 1l immune checkpoint inhibitor: a real-world outcomes analysis. J Immunother Cancer. 2023;11(1):e005801. 36650021 10.1136/jitc-2022-005801 PMC9853253

[242] Zheng M. Tumor mutation burden for predicting immune checkpoint blockade response: the more, the better. J Immunother Cancer. 2022;10(1):e003087. 35101940 10.1136/jitc-2021-003087 PMC8804687

[243] Si H, Kuziora M, Quinn K, Helman E, Ye J, Liu F, et al. A blood-based assay for assessment of tumor mutational burden in first-line metastatic nsclc treatment: results from the mystic study. Clin Cancer Res. 2020;27(6):1631–40. 33355200 10.1158/1078-0432.CCR-20-3771

[244] Ahmed J. Challenges and future directions in the management of tumor mutational burden-high (TMB-H) advanced solid malignancies. Cancers (Basel). 2023;15(24):5841. 38136385 10.3390/cancers15245841 PMC10741991

[245] Pian G, Shin J, Yoon S, Oh S. Prognostic reappraisal of postoperative carcinoembryonic antigen in T1–2N0 colorectal cancer. Anticancer Res. 2021;41(2):1101–10. 33517321 10.21873/anticanres.14868

[246] Tancoš V, Blichárová A. Predictive biomarkers of response to immunotherapy in triple-negative breast cancer—state of the art and future perspectives. Klin Onkol. 2023;36(1):28. 36868830 10.48095/ccko202328

[247] Mazerolles F, Rieux-Laucat F. PD-L1 is expressed on human activated naive effector CD4+ T cells. Regulation by dendritic cells and regulatory CD4+ T cells. PLoS ONE. 2021;16(11):e0260206. 10.1371/journal.pone.0260206. 34793567 10.1371/journal.pone.0260206 PMC8601581

[248] Peng Q, Qiu X, Zhang Z, Zhang S, Zhang Y, Liang Y, et al. PD-L1 on dendritic cells attenuates T cell activation and regulates response to immune checkpoint blockade. Nat Commun. 2020;11(1):4835. 32973173 10.1038/s41467-020-18570-x PMC7518441

[249] Jiménez-Reinoso A, Nehme-Álvarez D, Domínguez-Alonso C, Álvarez-Vallina L. Synthetic TILs: engineered tumor-infiltrating lymphocytes with improved therapeutic potential. Front Oncol. 2021. 10.3389/fonc.2020.593848/full. 33680923 10.3389/fonc.2020.593848 PMC7928359

[250] Xavier CB, Lopes CDH, Awni BM, Campos EF, Alves JPB, Camargo AA, et al. Interplay between tumor mutational burden and mutational profile and its effect on overall survival: a pilot study of metastatic patients treated with immune checkpoint inhibitors. Cancers (Basel). 2022;14(21):5433. 36358851 10.3390/cancers14215433 PMC9657500

[251] Balança CC, Salvioni A, Scarlata CM, Michelas M, Martinez-Gomez C, Gomez-Roca C, et al. PD-1 blockade restores helper activity of tumor-infiltrating, exhausted PD-1hiCD39+ CD4 T cells. JCI Insight. 2021. 10.1172/jci.insight.142513. 33332284 10.1172/jci.insight.142513 PMC7934837

[252] Caushi JX, Zhang J, Ji Z, Vaghasia A, Zhang B, Hsiue EHC, et al. Transcriptional programs of neoantigen-specific TIL in anti-PD-1-treated lung cancers. Nature. 2021;596(7870):126–32. 34290408 10.1038/s41586-021-03752-4 PMC8338555

[253] Harrasser M, Gohil SH, Lau H, Della Peruta M, Muczynski V, Patel D, et al. Inducible localized delivery of an anti-PD-1 scFv enhances anti-tumor activity of ROR1 CAR-T cells in TNBC. Breast Cancer Res. 2022;24(1):39. 10.1186/s13058-022-01531-1. 35659040 10.1186/s13058-022-01531-1 PMC9166313

[254] Simon S, Labarriere N. PD-1 expression on tumor-specific T cells: friend or foe for immunotherapy? Oncoimmunology. 2018;7(1):e1364828. 10.1080/2162402X.2017.1364828. 10.1080/2162402X.2017.1364828 PMC5739549 29296515

[255] Nguyen LT, Yen PH, Nie J, Liadis N, Ghazarian D, Al-Habeeb A, et al. Expansion and characterization of human melanoma tumor-infiltrating lymphocytes (TILs). PLoS ONE. 2010;5(11):e13940. 10.1371/journal.pone.0013940. 21085676 10.1371/journal.pone.0013940 PMC2978109

[256] Li Y, Liu S, Hernandez J, Vence L, Hwu P, Radvanyi L. MART-1–specific melanoma tumor-infiltrating lymphocytes maintaining CD28 expression have improved survival and expansion capability following antigenic restimulation in vitro. J Immunol. 2010;184(1):452–65. 19949105 10.4049/jimmunol.0901101

[257] Galsky M, Saci A, Szabó P, Han G, Grossfeld G, Collette S, et al. Nivolumab in patients with advanced platinum-resistant urothelial carcinoma: efficacy, safety, and biomarker analyses with extended follow-up from checkmate 275. Clin Cancer Res. 2020;26(19):5120–8. 32532789 10.1158/1078-0432.CCR-19-4162 PMC8166422

[258] Liu L, Bai X, Wang J, Tang X, Wu D, Du S, et al. Combination of TMB and CNA stratifies prognostic and predictive responses to immunotherapy across metastatic cancer. Clin Cancer Res. 2019;25(24):7413–23. 31515453 10.1158/1078-0432.CCR-19-0558

[259] Argentiero A, Solimando AG, Brunetti O, Calabrese A, Pantano F, Iuliani M, et al. Skeletal metastases of unknown primary: biological landscape and clinical overview. Cancers (Basel). 2019;11(9):1270. 31470608 10.3390/cancers11091270 PMC6770264

[260] Han C, Sasson A, Srinivasan S, Golhar R, Greenawalt D, Geese W, et al. Bioinformatic methods and bridging of assay results for reliable tumor mutational burden assessment in non-small-cell lung cancer. Mol Diagn Ther. 2019;23(4):507–20. 31250328 10.1007/s40291-019-00408-y PMC6675777

[261] Shadbad MA, Safaei S, Brunetti O, Derakhshani A, Lotfinejad P, Mokhtarzadeh A, et al. A systematic review on the therapeutic potentiality of PD-L1-inhibiting microRNAs for triple-negative breast cancer: toward single-cell sequencing-guided biomimetic delivery. Genes (Basel). 2021;12(8):1206. 34440380 10.3390/genes12081206 PMC8391239

[262] Vaxevanis C, Bachmann M, Seliger B. Immune modulatory micrornas in tumors, their clinical relevance in diagnosis and therapy. J Immunother Cancer. 2024;12(8):e009774. 10.1136/jitc-2024-009774. 39209767 10.1136/jitc-2024-009774 PMC11367391

[263] Wang M, Wang S, Desai J, Trapani JA, Neeson PJ. Therapeutic strategies to remodel immunologically cold tumors. Clin Transl Immunol. 2020. 10.1002/cti2.1226. 10.1002/cti2.1226 PMC8809427 35136604

[264] Daveri E, Vergani E, Shahaj E, Bergamaschi L, La Magra S, Dosi M, et al. microRNAs shape myeloid cell-mediated resistance to cancer immunotherapy. Front Immunol. 2020. 10.3389/fimmu.2020.01214/full. 32793185 10.3389/fimmu.2020.01214 PMC7387687

[265] Sloane RAS, White MG, Witt RG, Banerjee A, Davies MA, Han G, et al. Identification of microRNA–mRNA networks in melanoma and their association with PD-1 checkpoint blockade outcomes. Cancers (Basel). 2021;13(21):5301. 34771465 10.3390/cancers13215301 PMC8582574

[266] Wang H. MicroRNAs and apoptosis in colorectal cancer. Int J Mol Sci. 2020;21(15):5353. 32731413 10.3390/ijms21155353 PMC7432330

[267] Fang K, Tang DS, Yan CS, Ma J, Cheng L, Li Y, et al. Comprehensive analysis of necroptosis in pancreatic cancer for appealing its implications in prognosis, immunotherapy, and chemotherapy responses. Front Pharmacol. 2022. 10.3389/fphar.2022.862502/full. 35662734 10.3389/fphar.2022.862502 PMC9157651

[268] Walsh RJ, Sundar R, Lim JSJ. Immune checkpoint inhibitor combinations—current and emerging strategies. Br J Cancer. 2023;128(8):1415–7. 36747017 10.1038/s41416-023-02181-6 PMC10070427

[269] Vafaei S, Zekiy AO, Khanamir RA, Zaman BA, Ghayourvahdat A, Azimizonuzi H, et al. Combination therapy with immune checkpoint inhibitors (ICIs); a new frontier. Cancer Cell Int. 2022;22(1):2. 10.1186/s12935-021-02407-8. 34980128 10.1186/s12935-021-02407-8 PMC8725311

[270] Yin J, Gu T, Chaudhry N, Davidson NE, Huang Y. Epigenetic modulation of antitumor immunity and immunotherapy response in breast cancer: biological mechanisms and clinical implications. Front Immunol. 2024. 10.3389/fimmu.2023.1325615/full. 38268926 10.3389/fimmu.2023.1325615 PMC10806158

[271] Liu Y, Hu Y, Xue J, Li J, Yi J, Bu J, et al. Advances in immunotherapy for triple-negative breast cancer. Mol Cancer. 2023;22(1):145. 10.1186/s12943-023-01850-7. 37660039 10.1186/s12943-023-01850-7 PMC10474743

[272] Xu M, Lu J, Zhong Y, Jiang J, Shen Y, Su J, et al. Immunogenic cell death-relevant damage-associated molecular patterns and sensing receptors in triple-negative breast cancer molecular subtypes and implications for immunotherapy. Front Oncol. 2022. 10.3389/fonc.2022.870914/full. 35444934 10.3389/fonc.2022.870914 PMC9013947

[273] Wang Z, Wang B, Cao X. Epigenetic checkpoint blockade: new booster for immunotherapy. Signal Transduct Target Ther. 2021;6(1):281. 34294683 10.1038/s41392-021-00707-z PMC8298415

[274] Kan LLY, Chan BCL, Leung PC, Wong CK. Natural-product-derived adjunctive treatments to conventional therapy and their immunoregulatory activities in triple-negative breast cancer. Molecules. 2023;28(15):5804. 37570775 10.3390/molecules28155804 PMC10421415

[275] Qin J, Li Y, Zhang J, Zhang W. Stat3 as a potential therapeutic target in triple negative breast cancer: a systematic review. J Exp Clin Cancer Res. 2019;38(1):195. 31088482 10.1186/s13046-019-1206-z PMC6518732

[276] Li X, Yan S, Yang J, Wang Y, Lv C, Li S, et al. Efficacy and safety of pd-1/pd-l1 inhibitors plus chemotherapy versus pd-1/pd-l1 inhibitors in advanced non-small cell lung cancer: a network analysis of randomized controlled trials. Front Oncol. 2021;10:574752. 33585195 10.3389/fonc.2020.574752 PMC7873939

[277] Wang C, Qiao W, Jiang Y, Zhu M, Shao J, Wang T, et al. The landscape of immune checkpoint inhibitor plus chemotherapy versus immunotherapy for advanced non-small-cell lung cancer: a systematic review and meta-analysis. J Cell Physiol. 2019;235(5):4913–27. 31693178 10.1002/jcp.29371 PMC7028135

[278] Lee DW, Han SW, Bae JM, Jang H, Han H, Kim H, et al. Tumor mutation burden and prognosis in patients with colorectal cancer treated with adjuvant fluoropyrimidine and oxaliplatin. Clin Cancer Res. 2019;25(20):6141–7. 31285374 10.1158/1078-0432.CCR-19-1105

[279] Ruel LJ, Li Z, Gaudreault N, Henry C, Saavedra Armero V, Boudreau DK, et al. Tumor mutational burden by whole-genome sequencing in resected NSCLC of never smokers. Cancer Epidemiol Biomarkers Prev. 2022;31(12):2219–27. 36126278 10.1158/1055-9965.EPI-22-0630 PMC9720425

[280] Li W, Zhou K, Li M, Hu Q, Wei W, Liu L, et al. Identification of SCN7A as the key gene associated with tumor mutation burden in gastric cancer. BMC Gastroenterol. 2022;22(1):45. 10.1186/s12876-022-02112-4. 35123417 10.1186/s12876-022-02112-4 PMC8817579

[281] Liu K, Qin Z, Xu X, Li T, Ge Y, Mao H, et al. Comparative risk of renal adverse events in patients receiving immune checkpoint inhibitors: a Bayesian network meta-analysis. Front Oncol. 2021;11:662731. 34221977 10.3389/fonc.2021.662731 PMC8242344

[282] Yang W, Men P, Xue H, Jiang M, Luo Q. Risk of gastrointestinal adverse events in cancer patients treated with immune checkpoint inhibitor plus chemotherapy: a systematic review and meta-analysis. Front Oncol. 2020;10:197. 32211312 10.3389/fonc.2020.00197 PMC7076172

[283] Suazo-Zepeda E, Bokern M, Vinke P, Hiltermann T, Bock G, Sidorenkov G. Risk factors for adverse events induced by immune checkpoint inhibitors in patients with non-small-cell lung cancer: a systematic review and meta-analysis. Cancer Immunol Immunother. 2021;70(11):3069–80. 34195862 10.1007/s00262-021-02996-3 PMC8505368

[284] Shao F, Duan Y, Zhao Y, Li Y, Li J, Zhang C, et al. Parp inhibitors in breast and ovarian cancer with brca mutations: a meta-analysis of survival. Aging (Albany NY). 2021;13(6):8975–88. 33705352 10.18632/aging.202724 PMC8034970

[285] Nan Z, Guoqing W, Xiaoxu Y, Yin M, Xin H, Xue L, et al. The Predictive efficacy of tumor mutation burden (TMB) on nonsmall cell lung cancer treated by immune checkpoint inhibitors: a systematic review and meta-analysis. Biomed Res Int. 2021;2021:1–13. 33791360 10.1155/2021/1780860 PMC7984892

[286] Li S, Zhang C, Pang G, Wang P. Emerging blood-based biomarkers for predicting response to checkpoint immunotherapy in non-small-cell lung cancer. Front Immunol. 2020;11:603157. 33178229 10.3389/fimmu.2020.603157 PMC7596386

[287] Kern SE. Why your new cancer biomarker may never work: recurrent patterns and remarkable diversity in biomarker failures. Cancer Res. 2012;72(23):6097–101. 23172309 10.1158/0008-5472.CAN-12-3232 PMC3513583

[288] Knepper TC, Montesion M, Russell JS, Sokol ES, Frampton GM, Miller VA, et al. The genomic landscape of Merkel cell carcinoma and clinicogenomic biomarkers of response to immune checkpoint inhibitor therapy. Clin Cancer Res. 2019;25(19):5961–71. 31399473 10.1158/1078-0432.CCR-18-4159 PMC6774882

[289] Li Z, Feng Y, Li P, Wang S, Liu X, Xia S. CD1B is a potential prognostic biomarker associated with tumor mutation burden and promotes antitumor immunity in lung adenocarcinoma. Int J Gen Med. 2022;15:3809–26. 35418778 10.2147/IJGM.S352851 PMC9000921

[290] Chen X, Ou Z, Wang L, Zhang Z, Fan X, Liu H, et al. Association of tumor mutational burden with genomic alterations in Chinese urothelial carcinoma. Mol Carcinog. 2022;61(3):311–21. 34729830 10.1002/mc.23368

