# Supplementary material for: TMB as a predictive biomarker for ICI response in TNBC: current evidence and future directions for augmented anti-tumor responses
Source: Clin Exp Med. 2025 Nov 18;26(1):25. doi: 10.1007/s10238-025-01892-9 (PMC12628467; doi:10.1007/s10238-025-01892-9)
Supplement: Supplementary file 1 — (DOCX 65 kb) [file 10238_2025_1892_MOESM1_ESM.docx]

**Supplementary Data**

**Table 1.** Therapeutic avenues for TNBC

| Sl. No. | Avenues in TNBC treatment | Mechanism | Advantages | Setbacks | References |
| --- | --- | --- | --- | --- | --- |
| 1. | DNA damage response inhibitors (DDR inhibitor) | DDR inhibitors target TNBC cells' reliance on DDR pathways. TNBC has genetic alterations in DDR pathways, such as p53 dysfunction and BRCA1/2 mutations. Synthetic lethality is induced by exploiting these vulnerabilities with DDR inhibitors. The ATR-CHK1-WEE1 pathway and the HRR process are the most studied DDR pathways in TNBC. Combination therapy with DDR inhibitors and other DNA-damaging therapies is crucial. Effective predictive biomarkers are needed for maximum clinical utility. | The frequent genetic alterations in DDR pathway, including p53 dysfunction and BRCA1/2 mutations indicate the promising use of DDR inhibitors in treating TNBC. AZD1775 monotherapy is effective against TNBC with DDR deficiency or upregulated oncogenes. Combining DDR inhibitors with chemotherapy or drugs targeting DDR pathways is also effective in preclinical studies. | TNBC's high heterogeneity is a challenge for targeted drug development. DDR inhibitors have limited mono-therapy efficacy. Combination therapy with DNA-damaging therapies is crucial for DDR inhibitor effectiveness. Predictive biomarkers are needed for DDR inhibitor clinical utility maximization. | (1) |
| 2. | Cancer stem cells in TNBC (CSCs in TNBC) | The treatment for TNBC includes targeting and treating CSCs which are responsible for tumor growth and resistance to therapies. Advanced techniques help identify CSC clusters, biomarkers and micro environmental cells. Combining therapies with immunotherapy holds promise in developing a uniform TNBC treatment. | Cancer stem cells are crucial for TNBC tumor growth, drug resistance, and metastasis. Identifying and targeting these cells is the goal of TNBC treatment. Although single-cell analysis technologies can detect CSCs and biomarkers, having cancer stem cells in TNBC treatment does not provide any benefits. | Identifying and targeting CSCs is difficult due to their rarity and heterogeneity. CSCs can adapt and develop resistance to treatments due to their plasticity. Distinguishing CSCs from normal stem cells can lead to unintended side effects as there are no specific markers. Comprehensive molecular characterization of each TNBC case is required due to the complexity of the tumor microenvironment, which can be time-consuming and expensive. | (2) |
| 3. | Targeted Therapies in TNBC | Targeted therapies, like PARP inhibitors and immune check-point inhibitors, target TNBC molecular pathways. Synthetic lethality is induced in BRCA deficient tumors and the immune system is activated to attack cancer cells. Inhibition of signaling pathways that promote cell growth is achieved. Personalized treatment is based on biomarker expression using precision medicine and next generation sequencing. | Targeted therapies improve treatment effectiveness by focusing on specific molecular pathways in tumor progression, with promising results seen in TNBC through PARP inhibitors and immune check-point inhibitors. Molecular biomarkers guide tailored strategies, allowing for targeted therapies that minimize side effects and overcome challenges posed by tumor heterogeneity and clonal evolution, while precision medicine and next-gen sequencing open up new possibilities for novel therapeutic agents and enriched care for TNBC patients. | Targeted therapies may not be accessible to all patients. Treatment failure and disease progression can occur due to resistance. Side effects can impact the patient's quality of life. Biomarkers are necessary for targeted therapies but may not be present in all TNBC patients. Development is slow and expensive, limiting new treatment options. | (3) |
| 4. | Heat Shock Proteins (HSPs) in TNBC Treatment | HSP inhibitors have been developed to target multiple pathways in TNBC, including the PI3K/AKT/mTOR pathway, by inhibiting HSPs, which can induce apoptosis and inhibit tumor growth due to over expressed HSPs in TNBC. | HSP inhibitors can induce apoptosis and inhibit tumor growth. HSP inhibitors can overcome drug resistance in TNBC. Targeting HSPs is a promising therapeutic approach for TNBC. HSP inhibitors have potent anti-tumor activity in preclinical studies and are being evaluated in clinical trials. | HSP inhibitors may produce toxicity and off-target effects. They may not work for all TNBC patients and resistance can develop. Combining these inhibitors with other agents may be necessary for optimal results. Limited penetration into the tumor microenvironment and bioavailability can reduce effectiveness, necessitating frequent dosing. | (4) |
| 5. | Co-targeting cancer-associated fibroblasts and angiogenic vessel as a treatment for TNBC | ProAgio targets integrin αvβ3 on cancer-associated fibroblasts (CAFs) and angiogenic endothelial cells (aECs) in TNBC to induce apoptosis. By depleting CAFs, ProAgio reduces intratumoral collagen and growth factors, decreasing cancer cell proliferation and apoptotic resistance. ProAgio also eliminates tumor angiogenic vessels, reducing hypoxia and improving drug delivery, while decreased lysyl oxidase secretion potentially reduces metastasis. Studies in TNBC murine models demonstrate that ProAgio offers a survival benefit, highlighting its potential as a TNBC treatment strategy | Concurrently targeting CAFs and angiogenic vessels is crucial in the treatment of TNBC. ProAgio, which utilizes integrin αvβ3 to induce apoptosis in CAFs and aECs, reduces CAFs and leaky angiogenic vessels, leading to decreased hypoxia and enhanced drug delivery. This ultimately results in reduced cancer cell proliferation and apoptosis resistance, potentially mitigating metastasis. In TNBC murine models, ProAgio, either alone or with chemotherapy, offers a survival advantage, highlighting its therapeutic potential. | N/A | (5) |
| 6. | Emerging Therapeutics for Patients with Triple-Negative Breast Cancer | New treatments for TNBC include PARP inhibitors, immune checkpoint inhibitors, and antibody-drug conjugates. The use of different therapies in combination is being studied. The treatment of TNBC is moving towards a more personalized approach. | Emerging therapies for TNBC treatment have improved outcomes for patients, including new post-neoadjuvant chemotherapy strategies, PARP inhibitors, immune checkpoint inhibitors, and antibody-drug conjugates. Combination of different therapies is also under clinical investigation. | Immunotherapy and PARP inhibitors are currently limited by PD-L1 positivity and gBRCA mutations, respectively. However, current research seeks to expand the use of immunotherapy, PARP inhibitors, and antibody-drug conjugates (ADCs) in TNBC, exploring their use in earlier disease stages (adjuvant and neoadjuvant) and overcoming existing limitations. | (6) |
| 7. | Role of PARP in TNBC: Mechanism of Inhibition, Clinical Applications, and Resistance. | PARP plays a crucial role in DNA repair pathways, including base excision repair (BER) mechanisms, homologous recombination (HR), and nonhomologous end-joining (NEJ) deficiency-based repair mechanisms. Inhibitors of PARP have shown promising results in killing cancer cells in BRCA-mutations. In TNBC, PARP inhibitors have been used in combination with chemotherapy. The mechanism of PARP inhibition in TNBC involves blocking the repair of DNA damage, leading to the death of cancer cells. | Atezolizumab is a standard first-line treatment for advanced TNBC with PD-L1-positive tumors. PARP inhibitors are approved for BRCA-mutated advanced TNBC, and sacituzumab govitecan is FDA-approved for previously treated metastatic TNBC. The KEYNOTE-522 trial supports adding pembrolizumab to neoadjuvant platinum-containing taxane-anthracycline regimens, significantly increasing pCR rates with a trend toward EFS benefit. Novel ADCs are also emerging as promising TNBC therapies. | PARP inhibitors have shown promising results in the treatment of TNBC, particularly when used alongside chemotherapy. However, like any other cancer therapy, PARP inhibitors also have limitations. One of the limitations of PARP inhibitors is the development of resistance to the treatment. Cancer cells can develop mechanisms to repair DNA damage, making them resistant to PARP inhibitors. Another limitation is that not all TNBC patients have BRCA mutations, which are the primary targets of PARP inhibitors. Therefore, PARP inhibitors may not be effective in all TNBC patients. | (7) |
| 8. | Targeted Therapies for Triple-Negative Breast Cancer: Combating a Stubborn Disease | Targeted therapy for TNBC uses drugs that selectively target molecules or pathways involved in cancer cell growth and spread. These drugs may block overactive proteins or enzymes in cancer cells or stimulate the immune system to attack them. Targeted therapy can be used alone or with chemotherapy or radiation to improve outcomes and reduce side effects by selectively targeting cancer cells. | TNBC targeted therapy employs drugs that selectively inhibit cancer-promoting molecules or pathways, curtailing their aberrant activity. These agents may impede hyperactive proteins or enzymes in cancer cells or bolster the immune system's assault on them. Combinatorial use with chemotherapy or radiation is plausible. Objectives encompass refining treatment efficacy, minimizing adverse effects, and precise targeting of malignancies while preserving healthy counterparts. | Initially, targeted therapy drugs tend to exceed traditional chemotherapy costs, curtailing treatment access. Moreover, they focus on specific molecular pathways overactive in cancer, but resistance via alternate pathways or mutations hampers efficacy. Although side effects are milder than chemotherapy, some encounter fatigue, nausea, or diarrhea. Lastly, due to Triple-Negative Breast Cancer's heterogeneous nature, not all patients benefit from targeted therapy due to distinct molecular profiles and varied treatment responses. | (8) |
| 9. | Checkpoint inhibitor therapy for metastatic TNBC | Checkpoint inhibitor therapy targets immune checkpoint proteins to restore the immune system's ability to attack cancer cells. In TNBC, this therapy has shown promise by blocking the PD-1/PD-L1 pathway and restoring T cell response. Atezolizumab and pembrolizumab have recently been FDA-approved for combination use with chemotherapy in PD-L1-positive TNBC that is locally recurrent unresectable and metastatic. | Checkpoint inhibitor therapy aims to restore the immune system's capacity to fight cancer with fewer side effects by targeting immune checkpoint proteins. Recent FDA approvals for locally recurrent unresectable and metastatic PD-L1-positive TNBC have been achieved through the blocking of the PD-1/PD-L1 pathway and restoration of tumor-specific T cell response. Combining the therapy with other treatments can enhance effectiveness, leading to better outcomes for patients with TNBC and inducing long-term response. | Checkpoint inhibitors exhibit varied efficacy in TNBC due to tumor-specific traits like PD-L1 expression. Immune-related adverse events, e.g., organ inflammation, emerge as notable side effects demanding vigilant oversight. Financial constraints and restricted insurance coverage can impede patient accessibility to this costly therapy. Tumor resistance may develop, curtailing long-term treatment success. Despite encouraging trial outcomes, checkpoint inhibitor therapy's enduring safety and efficacy in TNBC necessitate further investigation. | (9) |
| 10. | Ferroptosis | Ferroptosis is a cell death process for TNBC treatment. GPX4 inhibitors are effective for LAR subtype, which has upregulated oxidized phosphatidylethanolamines and glutathione metabolism. Combining GPX4 inhibitors and immune checkpoint inhibitors is proposed for LAR tumors. GPX4 inhibition induces tumor ferroptosis and enhances antitumor immunity, resulting in better therapeutic efficacy. Targeting ferroptosis can improve prognosis in immunotherapy cohorts. | The LAR subtype of TNBC can be targeted for treatment with ferroptosis inducers, such as GPX4 inhibitors. This can be combined with immune checkpoint inhibitors for better therapeutic response and improved antitumor immunity. Targeting ferroptosis can also overcome resistance to immunotherapy. LAR tumors can be exploited by targeting the GSH metabolism pathway. Multiomics analysis can provide insights into personalized treatment strategies in TNBC. | Validation of GPX4 and immune checkpoint inhibitors in clinical practice is necessary to overcome limitations of using ferroptosis for TNBC treatment. Correlation analysis alone may not capture all metabolic regulators of ferroptosis in TNBC. The antitumor activity of GPX4 inhibitor monotherapy in mouse cell line-derived xenograft model may not fully represent in vivo effectiveness. Further investigation and validation are needed to ensure the proposed treatment strategy is effective. | (10) |
| 11. | Degradation of PD-L1 by D-mannose facilitates TNBC treatment | The use of D-mannose reduces PD-L1 protein levels in TNBC cells, making them more susceptible to T cell killing. In mice with TNBC tumors, D-mannose improved the efficacy of PD-1 checkpoint blockade immunotherapy and radiotherapy. D-mannose is safe and effective through oral administration in mice. Using D-mannose supplements may improve outcomes with emerging TNBC therapies. | D-mannose is a natural and safe method for treating TNBC. It reduces PD-L1 levels and boosts PD-1 immune checkpoint inhibitors. It also increases TNBC cell sensitivity to radiation treatment and can be taken orally. Its multifaceted approach is superior to synthetic drugs. The mechanism of action is innovative and could lead to more targeted treatments. D-mannose enhances TNBC therapies, but further clinical research is needed. | Dosage and delivery methods need to be determined, and responses vary due to pharmacokinetics. Long-term safety and potential side effects also require investigation. Further study is needed to determine effectiveness and to identify responsive populations. D-mannose resistance may limit the durability of responses. Regulatory approval and manufacturing challenges also need to be considered. | (11) |

**Table 2.** Methods for TMB value estimation

| Sl. No. | Method for TMB estimation | Perceptual basis of TMB calculation | Advantage | Setbacks | Reference |
| --- | --- | --- | --- | --- | --- |
| 1. | Panel-based tumor mutational burden (TMB) measurement | A panel of genes is used to analyze DNA from tumor tissue samples. FFPE tissue samples to analyze panel based NGS assays. | The capacity to employ formalin-fixed and paraffin-embedded (FFPE) tissue samples, a practical method for detecting TMB in a clinical context, and the viability of analyzing tiny biopsies.  Capability to carry out in silico germline mutation filtering.  Capability to analyze druggable targets and evaluate TMB in a single assay.  The entire workflow can be completed within a few days. | The technical variability can be problematic for tumors with lower TMB, as even small differences in mutation numbers can affect classification as “TMB high” for threshold value of 10 or 20 muts/Mb. Recent reviews have explored factors affecting psTMB measurement, but a comprehensive study is lacking. There are studies which aim to analyze all sources of TMB variability but acknowledges limited sample size and focus on NSCLC. | (12) |
| 2. | Blood-based tumor mutation burden (bTMB) biomarker measurement. | The bTMB test examines 1.1 Mb of genomic sequence through hybridization capture. bTMB detection requires sufficient ctDNA, indicated by MSAF <1%. | The bTMB method has an advantage due to its readily available source material, which is less prone to sampling bias caused by tumor heterogeneity in biopsies from single sites at a single time point. | The bTMB approach has limitations in detecting bTMB with enough ctDNA (MSAF ≥ 1%). TMB as a predictive biomarker has variations, and surrogate biomarkers like tTMB and bTMB evaluate a phenotype indirectly connected to neoantigen load. This load is distal to anti-PD-L1/PD-1 therapy. It is necessary to improve assay technology and gain better knowledge of TMB and its biological effects to identify the population that may benefit most from checkpoint inhibitors. | (13) |
| 3. | TMB measurement by identifying SNVs and removal of deaminated bases in FFPE DNA. | TMB assessment is improved by eliminating deaminated nucleotides from FFPE DNA. The Oncomine™ Tumour Mutation Load Assay is used to determine TMB in this study. This method is a customised NGS panel that identifies SNVs, insertions, deletions, and copy number abnormalities in 409 genes. | Clinical practise and research employ FFPE tissue samples because they are easy to store and transport.  FFPE samples are sometimes the only DNA source for retroactive examinations due to their lengthy storage life.  The Oncomine™ Tumour Mutation Load Assay utilised in this study is specific to FFPE DNA, making it reliable and accurate for TMB identification.  Removing deaminated nucleotides from FFPE DNA improves TMB testing precision by preventing false positives. | As a targeted NGS panel, the Oncomine™ Tumour Mutation Load Assay can only identify mutations in the 409 genes that make up the panel. This might not fully represent TMB because it does not account for all tumor-specific mutations. The research does not address other potential sources of inaccuracy in TMB measurement and only discusses the removal of deaminated bases in FFPE DNA. | (14) |
| 4. | TMB measurement by whole exome sequencing (WES) | NGS quantifies cancer mutation load. TMB was initially measured via NGS for WES of tumor and non-tumor tissue. Targeted panels have higher sensitivity and coverage, serving low cancer cell concentration. Standardization is necessary for reliable, repeatable, and clinically useful TMB measurement. | WES offers a cost-effective means of identifying coding mutations driving tumor progression, and can be used to calculate TMB. Assays like FoundationOne, which target 315 genes and 1.1 Mb of coding genome, correlate well with WES-derived TMB. High TMB scores from WES have been linked to better immunotherapy outcomes. However, standardized WES methodologies for TMB measurement are lacking, potentially limiting its widespread use. | Targeted panels only include a subset of genes; therefore, they might not detect all cancer genome mutations. Targeted panel TMB measurement accuracy may be affected by DNA sample quality because formaldehyde fixation produces crosslinks and is a frequent source of sequencing artefacts. Also unexplored are the therapeutic implications of improving sequencing coverage sensitivity. | (15) |
| 5. | Estimating and classification of TMB (ecTMB) | The ecTMB technique estimates BMR and predicts TMB by treating each gene as an independent negative binomial process. TMB is predicted using synonymous mutations and samples are classified into TMB subtypes using a Gaussian Mixture Model. The ecTMB approach improves TMB measurement accuracy and reproducibility when using targeted panel sequencing data from TCGA. | The ecTMB method offers advantages for predicting tumor mutational burden (TMB). It improves consistency by correcting biases in panel design. It considers both synonymous and non-synonymous mutations for improved accuracy. It predicts TMB using a negative binomial process for robustness. It also reveals clinically relevant TMB subtypes and shows high concordance with standard TMB measurements. TMB readings can be more accurate and reproducible because ecTMB uses a Bayesian framework to describe the background mutation rate (BMR) and predict TMB. | WES is expensive and time-consuming, not preferrable for everyday clinical use. WES data may be noisy and error-prone, affecting TMB measurements. The current TMB calculation method does not consider mutation rate variation and can lead to inaccurate estimates. The clinical significance of the current TMB approach is limited as it does not reveal functional alterations. | (16) |
| 6. | NGS of a 2-megabase 500-gene panel. | Mutations (single nucleotide variations, insertions, and deletions) per megabase determined TMB. The authors measured TMB using NGS of a 2-megabase 500-gene panel. The panel spans two megabases and cancer-related genes. The tumor-normal whole exome sequencing (WES) or in-silico-filtered tumor-only 500-gene panel quantified TMB. The authors analyzed tumor-only WES TMB using computational germline-filtering and background noise-removal. | A tailored DNA sequencing panel that covers two megabases and includes cancer-relevant genes is cheaper than full exome sequencing.  TMB assessed by the 500-gene panel and WES correlate highly, with sensitivity >90% and specificity >85%.  When paired with bioinformatic germline-filtering and background noise-removal, tumour samples can be sequenced like subject-matched normal DNA. | N/A | (17) |
| 8. | Capture-based targeted sequencing method to measure TMB | The study used pleural effusion tissues from NSCLC patients to test three panels' effectiveness in determining TMB. All five panels evaluated showed strong correlations with TMB from WES, with values ranging from 0.68 to 0.81. The study concluded that all panels can be used for TMB evaluation based on PE samples. | Capture-based targeted sequencing offers advantages for measuring tumor mutation burden (TMB), presenting a practical alternative to whole-exome sequencing (WES) with its benefits of reduced sample quantities, cost, and data management complexity. Employing this method on pleural effusion (PE) samples, this study successfully correlated TMB values with WES results, highlighting PE's potential as an alternative TMB evaluation medium. Standardizing TMB estimation through capture-based targeted sequencing establishes a foundation for future protocols and TMB's integration into clinical practice. | Inconsistencies in TMB estimation can occur due to inter-panel and inter-laboratory variations. TMB estimation using capture-based targeted sequencing is more consistent than detection of fusion events and copy number changes. Underestimation of TMB can happen due to variations in panel size and targeted genes. Deviation from TMB estimation can be caused by several factors. Further research is needed to understand the utility of pleural effusion as a sample for TMB evaluation. | (18) |
| 9. | THetA2 method to measure TMB | The THetA2 program utilizes a probabilistic model to deduce tumor composition from high-throughput DNA sequencing data. It gauges the percentage of normal cells, tumor subpopulations, and sample purity by analyzing copy number aberrations and B-allele frequencies. The system can also detect subclonal copy number aberrations and identify subclonal populations and severely altered genomes. | THetA2 is a superior algorithm for analyzing rearranged genomes with copy number aberrations in tumor subpopulations. It uses various data types and is faster than the previous version. THetA2 can identify subclonal populations in highly rearranged samples and provide accurate estimates of tumor purity and subpopulations from WGS and WXS sequencing data. | There are no THetA2 algorithm constraints. However, like any computational method, it may have limits in specific situations or data types. Always carefully analyse analysis results and consider potential errors or biases. | (19) |
| 10. | Germline substruction method to measure TMB | To quantify TMB by germline subtraction, sequence both tumor and normal (germline) samples and subtract the germline variations from the tumor variants to get somatic mutations. Somatic mutations are used to determine TMB, the number of mutations per genome megabase. This is the most accurate somatic mutation determination and TMB calculation approach. | The Germline subtraction technique measures TMB with high accuracy by excluding germline variants. It focuses on tumor-specific mutations to provide a precise estimation of somatic alterations. The method prevents sampling bias and avoids inaccurate categorization of tumors. It is particularly useful for paired tumor-normal samples to enable direct comparison and reliable measurement. Germline subtraction standardizes TMB calculation, ensuring consistency in assessment across tumor types and platforms. | Germline variant discovery requires sequencing a normal (germline) sample, which raises testing costs. The study's sample size was small; thus, filtering approaches may have stronger connections with some tumor types. The study did not account for tumor purity, which may be linked to TMB. The study concludes that multiple sequencing methods may be needed to accurately determine TMB. | (20) |

References:

1. Jin J, Tao Z, Cao J, Li T, Hu X. DNA damage response inhibitors: An avenue for TNBC treatment. Biochim Biophys Acta BBA - Rev Cancer. 2021 Apr 1;1875(2):188521.

2. O’Conor CJ, Chen T, González I, Cao D, Peng Y. Cancer stem cells in triple-negative breast cancer: a potential target and prognostic marker. Biomark Med. 2018 Jul;12(7):813–20.

3. Vagia E, Mahalingam D, Cristofanilli M. The Landscape of Targeted Therapies in TNBC. Cancers. 2020 Apr;12(4):916.

4. Tsai CH, Weng JR, Lin HW, Lu MT, Liu YC, Chu PC. Targeting Triple Negative Breast Cancer Stem Cells by Heat Shock Protein 70 Inhibitors. Cancers. 2022 Oct 7;14(19):4898.

5. Sharma M, Turaga RC, Yuan Y, Satyanarayana G, Mishra F, Bian Z, et al. Simultaneously targeting cancer-associated fibroblasts and angiogenic vessel as a treatment for TNBC. J Exp Med. 2021 Apr 5;218(4):e20200712.

6. Agostinetto E, Eiger D, Punie K, de Azambuja E. Emerging Therapeutics for Patients with Triple-Negative Breast Cancer. Curr Oncol Rep. 2021 Mar 24;23(5):57.

7. Singh DD, Parveen A, Yadav DK. Role of PARP in TNBC: Mechanism of Inhibition, Clinical Applications, and Resistance. Biomedicines. 2021 Oct 21;9(11):1512.

8. Kalimutho M, Parsons K, Mittal D, López JA, Srihari S, Khanna KK. Targeted Therapies for Triple-Negative Breast Cancer: Combating a Stubborn Disease. Trends Pharmacol Sci. 2015 Dec;36(12):822–46.

9. Heeke AL, Tan AR. Checkpoint inhibitor therapy for metastatic triple-negative breast cancer. Cancer Metastasis Rev. 2021;40(2):537–47.

10. Fan Yang, Yi Xiao, Jia-Han Ding, Xi Jin, Ding Ma, Da-Qiang Li, et al. Ferroptosis heterogeneity in triple-negative breast cancer reveals an innovative immunotherapy combination strategy. Cell Metab. 2022 Oct 1;

11. Ruonan Zhang, Yang Y, Yang RY, Wenjing Dong, Mingen Lin, Jing He, et al. D-mannose facilitates immunotherapy and radiotherapy of triple-negative breast cancer via degradation of PD-L1. Proc Natl Acad Sci U S A. 2022 Feb 18;119(8):e2114851119–e2114851119.

12. Budczies J, Kazdal D, Allgäuer M, Christopoulos P, Rempel E, Pfarr N, et al. Quantifying potential confounders of panel-based tumor mutational burden (TMB) measurement. Lung Cancer. 2020 Apr 1;142:114–9.

13. Kim ES, Velcheti V, Mekhail T, Yun C, Shagan SM, Hu S, et al. Blood-based tumor mutational burden as a biomarker for atezolizumab in non-small cell lung cancer: the phase 2 B-F1RST trial. Nat Med. 2022 May;28(5):939–45.

14. Tom W, Chaudhary R, Mittal V, Cyanam D, Casuga I, Wong-Ho E, et al. Abstract 1701: Improvement of tumor mutation burden measurement by removal of deaminated bases in FFPE DNA. Cancer Res. 2019 Jul 1;79(13_Supplement):1701.

15. Meléndez B, Campenhout CV, Rorive S, Remmelink M, Salmon I, D’Haene N. Methods of measurement for tumor mutational burden in tumor tissue. Transl Lung Cancer Res [Internet]. 2018 Dec [cited 2023 Aug 2];7(6). Available from: https://tlcr.amegroups.org/article/view/23823

16. Yao L, Fu Y, Mohiyuddin M, Lam HYK. ecTMB: a robust method to estimate and classify tumor mutational burden. Sci Rep. 2020 Mar 18;10(1):4983.

17. So AS, Kaplan S, Zhao C, Zhang S, Liu L, Le P, et al. Abstract 435: Accurate measurement of tumor mutation burden through tumor-only sequencing using a 500-gene panel. Cancer Res. 2018 Jul 1;78(13_Supplement):435.

18. Yu Y, Shen L, Ji W, Lu S. Standardization of pleural effusion-based tumor mutation burden (TMB) estimation using capture-based targeted sequencing. Ann Transl Med. 2021 Jan;9(2):140–140.

19. Oesper L, Satas G, Raphael BJ. Quantifying tumor heterogeneity in whole-genome and whole-exome sequencing data. Bioinforma Oxf Engl. 2014 Dec 15;30(24):3532–40.

20. Parikh K, Huether R, White K, Hoskinson D, Beaubier N, Dong H, et al. Tumor Mutational Burden From Tumor-Only Sequencing Compared With Germline Subtraction From Paired Tumor and Normal Specimens. JAMA Netw Open. 2020 Feb 5;3(2):e200202.
